# Panicle-SEG: a robust image segmentation method for rice panicles in the field based on deep learning and superpixel optimization

**DOI:** 10.1186/s13007-017-0254-7

**Published:** 2017-11-28

**Authors:** Xiong Xiong, Lingfeng Duan, Lingbo Liu, Haifu Tu, Peng Yang, Dan Wu, Guoxing Chen, Lizhong Xiong, Wanneng Yang, Qian Liu

**Affiliations:** 1 Britton Chance Center for Biomedical Photonics, Wuhan National Laboratory for Optoelectronics, and Key Laboratory of Ministry of Education for Biomedical Photonics, Department of Biomedical Engineering, Huazhong University of Science and Technology, Wuhan, 430074 People’s Republic of China; 2 National Key Laboratory of Crop Genetic Improvement, National Center of Plant Gene Research, Agricultural Bioinformatics Key Laboratory of Hubei Province, and College of Engineering, Huazhong Agricultural University, Wuhan, 430070 People’s Republic of China

**Keywords:** Rice (*O. sativa*) panicles, Image segmentation, Deep learning, Convolutional neural network, Superpixel

## Abstract

**Background:**

Rice panicle phenotyping is important in rice breeding, and rice panicle segmentation is the first and key step for image-based panicle phenotyping. Because of the challenge of illumination differentials, panicle shape deformations, rice accession variations, different reproductive stages and the field’s complex background, rice panicle segmentation in the field is a very large challenge.

**Results:**

In this paper, we propose a rice panicle segmentation algorithm called Panicle-SEG, which is based on simple linear iterative clustering superpixel regions generation, convolutional neural network classification and entropy rate superpixel optimization. To build the Panicle-SEG-CNN model and test the segmentation effects, 684 training images and 48 testing images were randomly selected, respectively. Six indicators, including Qseg, Sr, SSIM, Precision, Recall and F-measure, are employed to evaluate the segmentation effects, and the average segmentation results for the 48 testing samples are 0.626, 0.730, 0.891, 0.821, 0.730, and 76.73%, respectively. Compared with other segmentation approaches, including HSeg, i2 hysteresis thresholding and jointSeg, the proposed Panicle-SEG algorithm has better performance on segmentation accuracy. Meanwhile, the executing speed is also improved when combined with multithreading and CUDA parallel acceleration. Moreover, Panicle-SEG was demonstrated to be a robust segmentation algorithm, which can be expanded for different rice accessions, different field environments, different camera angles, different reproductive stages, and indoor rice images. The testing dataset and segmentation software are available online.

**Conclusions:**

In conclusion, the results demonstrate that Panicle-SEG is a robust method for panicle segmentation, and it creates a new opportunity for nondestructive yield estimation.

**Electronic supplementary material:**

The online version of this article (10.1186/s13007-017-0254-7) contains supplementary material, which is available to authorized users.

## Introduction

**Fig. 1:**
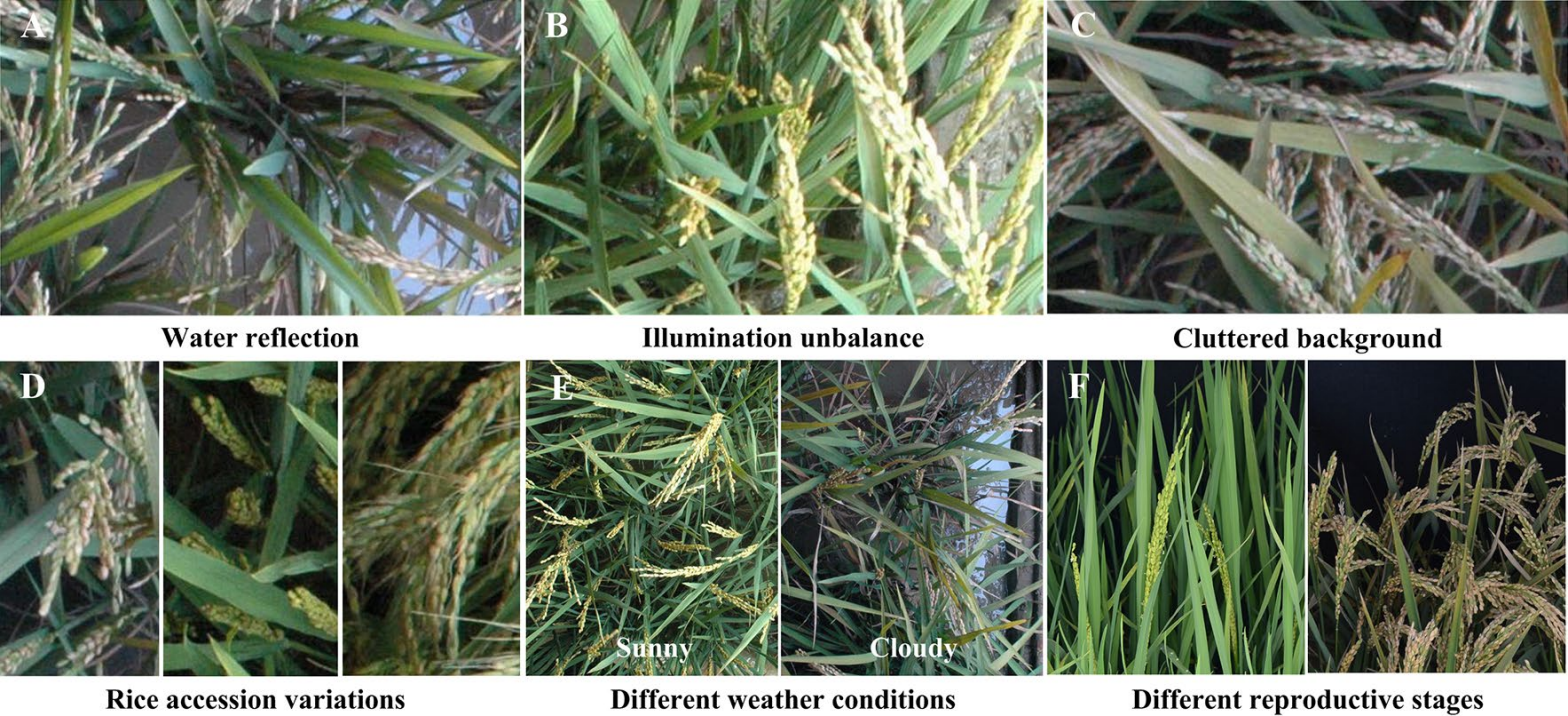
Challenges in the field based rice panicle segmentation. **A** Water reflection. **B** The illumination unbalance in the same plot. **C** Yellowish rice leaves and serious overlapping. **D** Variance in rice accession. **E** Different weather conditions. **F** Different reproductive stages

Rice (*O. sativa*) is an important primary food for a large proportion of the world’s population, especially in Asia [1–3]. Therefore, rapid screening for crops with high yield is extremely important for ensuring the safety of rice production and helping to address food shortage problems [4, 5]. The rice panicle, as an important agronomic component [6], not only is closely associated with yield [7, 8] but also plays an important role in disease detection [9], nutrition examination [10] and growth period determination [11]. Thus, accurate panicle segmentation is a key step in rice field phenotyping [12]. However, because of the complexity of the field environment (water reflections, illumination unbalance and cluttered background) (Fig. 1A–C), variations in the rice accessions (Fig. 1D), different weather conditions (Fig. 1E) and different reproductive stages (Fig. 1F), which cause differences in the colors, textures, size, and shapes in the panicle images, accurate panicle segmentation is an enormous challenge [13]. The existing segmentation methods mostly focus on two aspects. One aspect is solely based on color information. For example, Tang et al. proposed a method (HSeg) that relies on Hue plane threshold segmentation for the maize tassel [14]. Part of a corn tassel was extracted to locate the maize tassel. The disadvantage is that the same component under different illuminations will appear to have various colors. Thus, segmentation based on color information will be seriously affected by the illumination. Additionally, the color information changes with the reproductive stage. In this way, the type of method usually applies to a certain reproductive period. Except for the disadvantages described above, an excess dependence on color information will lead to the phenomenon of incomplete extraction. The other aspect is the two-step segmentation method. Among these, candidate region generation is the first step, and a general classifier is then adopted for the classification of candidate regions.

To generate candidate regions, several methods have been applied in the current research studies: (1) one of the ideas relies on threshold segmentation for different color channels. Similarly, Duan et al. applied an algorithm to extract potted rice panicles from multi-angle side-view images [12]. The hysteresis thresholding based on the i2 color plane is applied to extract the panicle candidate regions. Obviously, image segmentation of panicles in pot-grown rice, which is inspected in a chamber with a stable illumination environment, is relatively simple compared with phenotyping in field environments. (2) Another thought for candidate regions generation is utilizing a classifier, such as the support vector machine (SVM). The gray values for different color spaces, such as the RGB, HSV and LAB color planes, are calculated as the input vector of the SVM classifier. For example, Zhu et al. introduced a wheat ear detection mechanism to automatically observe the wheat heading stage [15]. The proposed method is applied to generate wheat ear candidate regions with SVM. In addition, Lu et al. developed a framework called mTASSEL-S to execute the maize tassel segmentation [2]. The color space conversion is used to make a coarse location easier with SVM. (3) Furthermore, there is the consideration that the candidate regions could rely on graph-based segmentation. Similarly, Lu et al. in 2016 proposed a method for maize tassel segmentation based on region-based color modeling [13]. The fusion of graph-based segmentation and superpixel generation achieves the division of candidate regions.

Afterward, the second step of the two-step segmentation method uses a general classifier that is based on SIFT features or other features (such as color features, morphological features and location features used by Duan et al. [12]) to classify the candidate regions. Guo et al. proposed a powerful method for automatically detecting flowering panicles of paddy rice in RGB images taken under natural field conditions [16]. Visual words, as a coding method, is developed to encode the SIFT features for each of the candidate regions. Then, the SVM classifier is used as a fine-detection method for the candidate regions. The concept adopted by Zhu et al. [15] is similar to the above approach. The difference is that another encoding algorithm (fisher vector encoding) is chosen instead of visual words. Overall, compared with segmentation that is based on only color information, the two-step segmentation is relatively robust.

To the best of our knowledge, few studies have investigated rice panicle segmentation. In this study, simple linear iterative clustering (SLIC) [17] is used to generate candidate regions, and convolutional neural network (CNN, one of the deep learning technologies) [18] is applied as a candidate region classifier. Afterward, the entropy rate superpixel (ERS) [19] algorithm is developed for segmentation result optimization. The results showed that our presented method can be expanded for the different field environments, different camera angles, different reproductive stages, and indoor rice images. Compared with several related segmentation algorithms, the proposed Panicle-SEG algorithm shows better performance regarding the segmentation accuracy.

## Results

### Image analysis pipeline of the Panicle-SEG algorithm

**Fig. 2:**
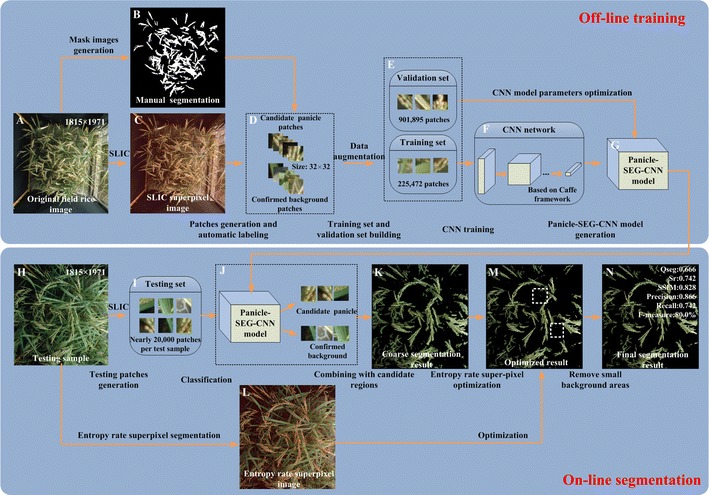
The flow diagram of the Panicle-SEG algorithm. **A** Original field rice image. **B** Mask image. **C** SLIC superpixel segmentation result. **D** Automatic labeling. **E** Training set and validation set building: The patches were augmented and divided into the training set and validation set. **F** CNN network. **G** Panicle-SEG-CNN model generation. **H** Testing sample. **I** Testing patches generation. **J** The pre-trained Panicle-SEG-CNN model generated in off-line training is utilized in testing patches classification, and the testing patches were categorized into candidate panicle and confirmed background. **K** The candidate panicle patches were merged into one image, called the coarse segmentation results. **L** Entropy rate superpixel image. **M** Optimized segmentation result. **N** The final segmentation result was obtained after removing small regions

**Fig. 3:**
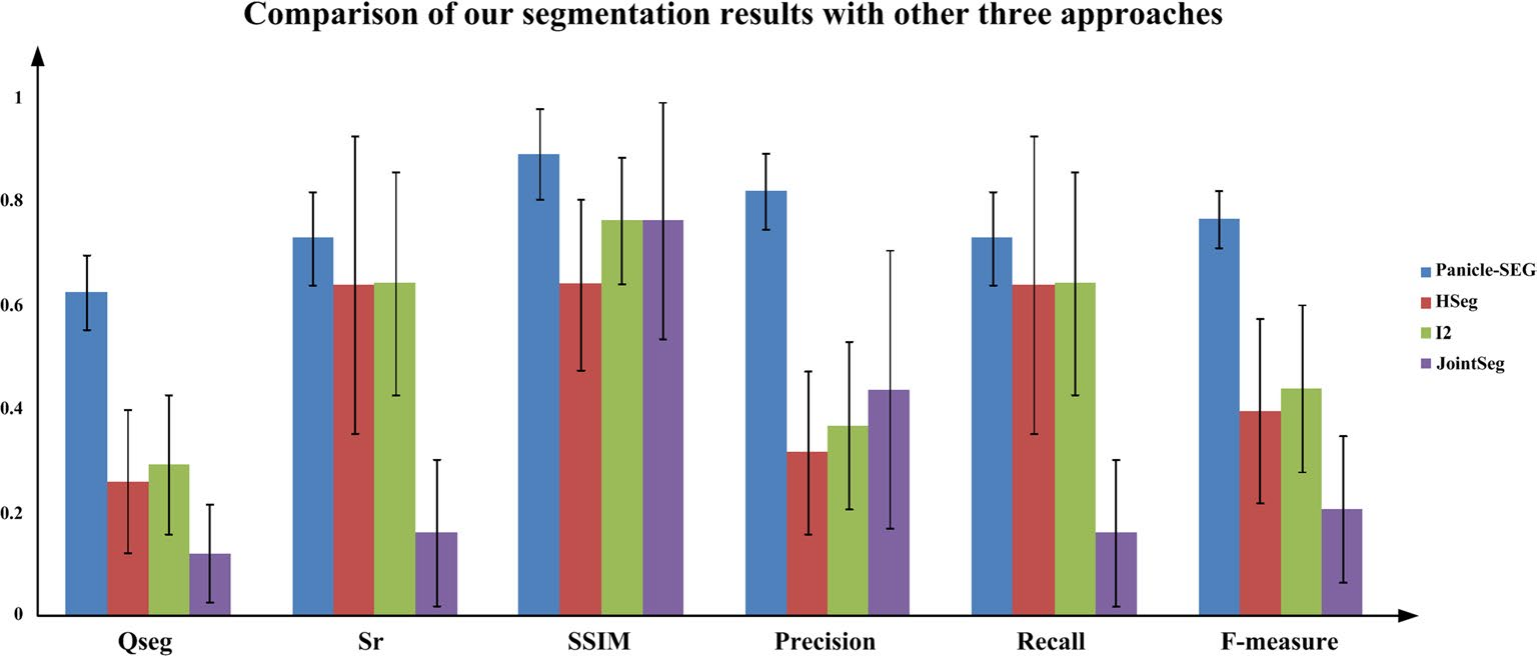
The means and standard deviations of six evaluation indicators for the testing set. Six indicators, including the Qseg, Sr, SSIM, Precision, Recall, and the F-measure (%) are adopted to evaluate the performance of the segmentation result. The color columns and black lines represent the means and standard deviations for the testing set, respectively. Additionally, the color differences of the columns show the various segmentation algorithms (Panicle-SEG, HSeg, i2 hysteresis thresholding, and jointSeg)

**Fig. 4:**
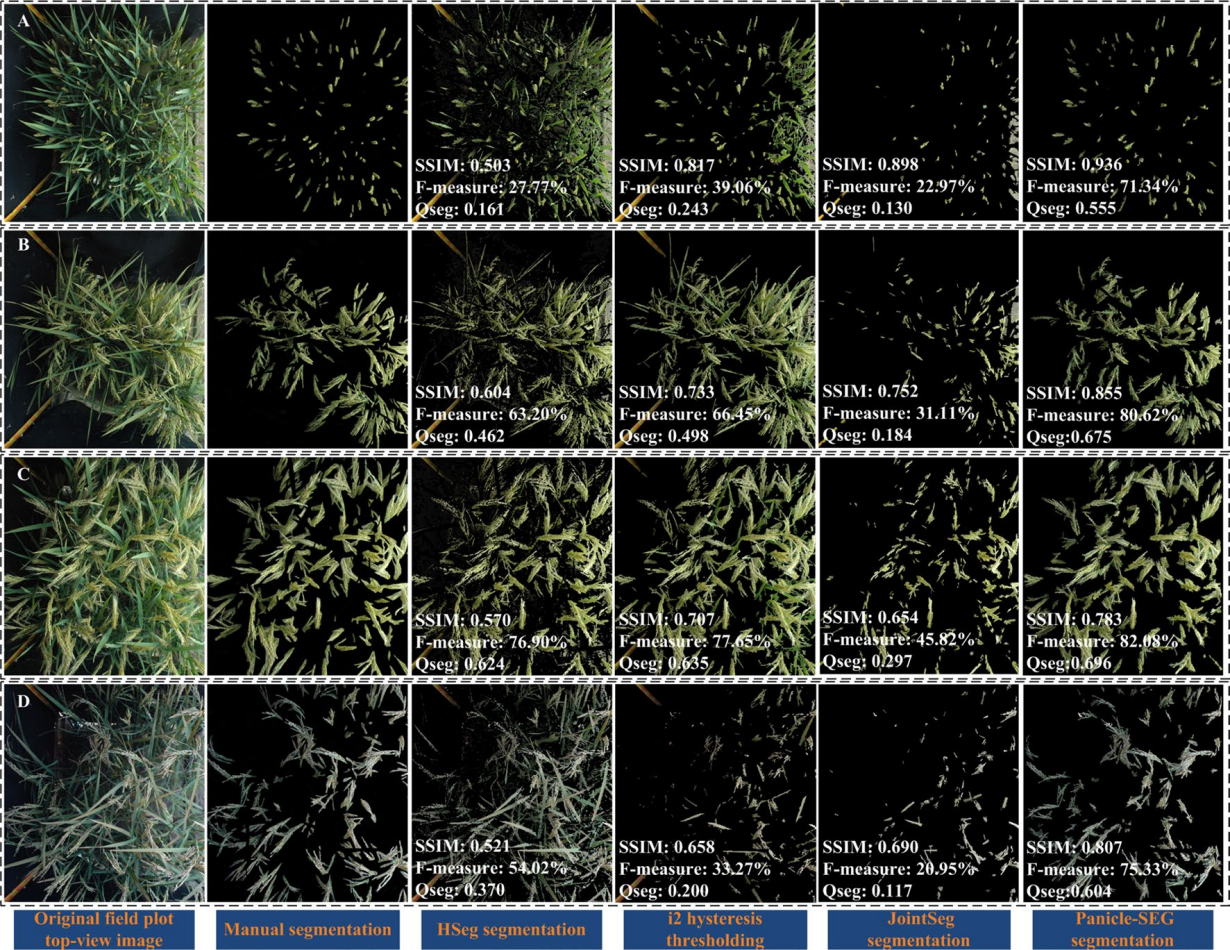
Comparison to state-of-the-art segmentation approaches. Four representative field rice images are selected to illustrate the segmentation effect. The first column reflects the original top-view rice images in the field. The second column is the manual panicle segmentation result using Photoshop software. The third column to the sixth column represents the rice panicle segmentation results using HSeg, i2 hysteresis thresholding, jointSeg, and Panicle-SEG algorithm. **A** The upright panicles are partially hidden in the rice leaf blade. **B** The bend growth panicles are basically exposed above the rice leaf blade. **C** The awn exists in the rice panicle, and the illumination is uneven in the same field plot rice image. **D** On a cloudy day, the panicle color appears to be gray

After the image acquisition, the main flow diagram of our rice panicle segmentation algorithm (Panicle-SEG algorithm) including off-line training and on-line segmentation is shown in Fig. 2. Here, 684 representative rice images, including 49 top-view field rice images, 30 overhead-view field rice images, 302 pot-grown side-view images and 303 pot-grown top-view images are selected to build the Panicle-SEG-CNN model, in which the illumination changes, weather conditions, panicle shapes, rice accessions, cluster background, reproductive stages and camera angle condition are all considered (Fig. 1). The detailed process of the proposed Panicle-SEG algorithm is described here as an example of the field top-view rice image (Fig. 2A). The off-line training contained 4 steps: (1) Generation of patches: all the images were manually segmented using Photoshop software to obtain the mask images (Fig. 2B) for the following automatic labeling. Patches were generated using SLIC superpixel segmentation (Fig. 2C); (2) Automatic labeling: the patches were automatically labeled into 2 categories: candidate panicle and confirmed background (Fig. 2D); (3) Training set and validation set building: The patches were augmented and divided into the training set (901,895 patches) and validation set (225,472 patches) (Fig. 2E); (4) CNN training and Panicle-SEG-CNN model generation (Fig. 2F, G). Then, for a testing sample (Fig. 2H), the on-line segmentation included 3 steps: (1) Generation of patches: The testing patches were generated using SLIC superpixel segmentation (Fig. 2I); (2) Coarse segmentation by using a pre-trained Panicle-SEG-CNN model: The pre-trained Panicle-SEG-CNN model generated in off-line training is utilized in testing patch classification, and the testing patches were categorized into candidate panicles and confirmed background (Fig. 2J). Then the candidate panicle patches were merged into one image, called the coarse segmentation result (Fig. 2K); (3) Entropy rate superpixel optimization: The coarse segmentation result was combined with the entropy rate superpixel image (Fig. 2L) to obtain the optimized segmentation result (Fig. 2M). The final segmentation result (Fig. 2N) was obtained after removing small background region.

### The processing efficiency of the Panicle-SEG algorithm

Using our computer system (Microsoft Windows 10 PC with a 4-core i5 CPU, 3.2 GHz per CPU core, 8 GB of memory and a NVIDA GTX 750ti video card), the segmentation process for one testing image with resolution of 1815 × 1971 pixels takes approximately 135–150 s. Utilizing multithreading (OpenMP) and CUDA parallel acceleration, the processing efficiency could be increased to approximately 70 s. Moreover, the Panicle-SEG algorithm was not restricted by the size of the input image, which means that it can address any size for the original input rice image, such as 800 × 600 pixels, etc. At the same time, with a decreased size of the input image to be processed, the time required will obviously be reduced. For example, when the resolution of the input image is 1392 × 1040 pixels, the segmentation time in the GPU mode is about 20–22 s. Furthermore, the CPU frequency, CPU cores and performance of the video card, to a large extent, will impact the time for segmentation. We pack the panicle segmentation project (Panicle-SEG) with an installer. The whole testing images and the Panicle-SEG installation file in CPU/GPU mode are available online at: http://plantphenomics.hzau.edu.cn/checkiflogin_en.action (username: UserPP; password: 20170108pp), and the detailed software implementation procedure is illustrated in Additional file 1: Video S1 and Additional file 2: Appendix S1. The detailed software implementation is as follows: (1) Install the “setup_Panicle-SEG_cpu.exe” or “setup_Panicle-SEG_gpu.exe” file. (2) Open the command line and enter to the current file path. (3) Open the “Readme.txt” and revise the parameters if needed. (4) Copy the revised content in “Readme.txt” and paste to the command line. (5) Waiting for the rice panicle segmentation (the cost time depends on your computer performance). (6) The segmentation results are shown in “segmentation_results” file.

### Performance evaluation of the testing set using six indicators

In this study, after 684 representative rice images were used to train the Panicle-SEG-CNN model, another 48 testing images, including 24 field-based top-view images, 12 field-based overhead-view images (heading stage, filling stage and mature stage), 12 indoor images (7 side-view and 5 top-view) were selected to test the segmentation algorithm. To evaluate the performance of the segmentation, six indicators, including the Qseg, Sr [20], Structural Similarity Index (SSIM) [21], Precision, Recall, and the F-measure [22] are adopted. Among them, the range of Qseg is from 0 to 1. Namely, the higher the value (approach to 1), the more accurate the segmentation is. Conversely, the closer the value is to 0, the worse the consistency is. So, the value of Qseg reflects the consistency of all the image pixels, including panicle foreground part and background part. And the value of Sr represents the consistency of only panicle part. From the perspective of an image, it reflects the completeness of the segmentation results. The computational formula for Qseg and Sr are provided in Eqs. 1, 2. The SSIM is applied to describe the degree of similarity between the segmentation images and the ground truth images. The SSIM model analyzes the structure of the image information from three aspects, including the brightness, structure similarity and contrast. The range of the SSIM is from 0 to 1, and the higher the value is, the more similar the two images are. Precision and Recall are the most basic indicators to reveal the final segmentation results. Precision illustrates the accuracy of the segmentation algorithm, and Recall represents the completeness of the segmented rice panicles. The computational formulas for Precision and Recall are provided in Eqs. 3, 4. In practice, Precision and Recall interact with each other. When Precision is high, Recall will be low. Sometimes, we need to balance these two indicators. To accomplish this goal, the F-measure is proposed. The computational formula is shown in Eq. 5. The higher the value of the F-measure is, the more perfect the rice panicle segmentation will be.

1 $$Q{\text{seg}} = \frac{{\sum\limits_{i = 0}^{n} {\sum\limits_{j = 0}^{m} {(\text{A} (v)_{i,j} \cap \text{B} (v)_{i,j} )} } }}{{\sum\limits_{i = 0}^{n} {\sum\limits_{j = 0}^{m} {(\text{A} (v)_{i,j} \cup \text{B} (v)_{i,j} )} } }}$$

2 $$Sr = \frac{{\sum\limits_{i = 0}^{n} {\sum\limits_{j = 0}^{m} {(\text{A} (v)_{i,j} \cap \text{B} (v)_{i,j} )} } }}{{\sum\limits_{i = 0}^{n} {\sum\limits_{j = 0}^{m} {\text{B} (v)_{i,j} } } }}$$

3 $$Precision = \frac{TP}{TP + FP}$$

4 $$Recall = \frac{TP}{TP + FN}$$

5 $$F = \frac{2 \times Precision \times Recall}{Precision + Recall} \times 1 0 0 {\text{ (\% )}}$$

where, A in Eqs. 1, 2 means the panicle pixels (v = 255) or background pixels (v = 0) segmented by our Panicle-SEG, and B in Eqs. 1, 2 represents a reference set of manually segmented panicle pixels (v = 255) or background pixels (v = 0). The value of m and n reflects the image row and column and i, j are the pixel coordinate of the images. In Eqs. 3–5, the *TP*, *TN*, *FP*, and *FN* represent the numbers of true positives, true negatives, false positives, and false negatives, respectively. True positives (*TP*) are when the predicted results and the corresponding ground truth are both rice panicle pixels. True negatives (*TN*) represent that the predicted results and the corresponding ground truth are both background pixels. False positives (*FP*) were determined as those pixels that were classified as rice panicle pixels, but the ground truth of those pixels are background. The False negatives (*FN*) are those pixels that belong to the ground truth, but they are not correctly discriminated.

For the Panicle-SEG segmentation algorithm, the mean values of the Qseg, Sr, SSIM, Precision, Recall and the F-measure (%) were 0.626, 0.730, 0.891, 0.821, 0.730, and 76.73%, respectively. The standard deviations of the Qseg, Sr, SSIM, Precision, Recall, and the F-measure (%) for 48 testing samples were 0.072, 0.090, 0.088, 0.074, 0.090, and 5.46% respectively. The performance and the final rice panicle segmented results for 48 testing images are listed in Additional file 3: Table S1.

### Comparison of Panicle-SEG segmentation results with the other three approaches

To verify the superiority of the Panicle-SEG algorithm, the other three well-established algorithms, including HSeg [14], i2 hysteresis thresholding [12], and jointSeg [13], were used. The means and standard deviations of the six evaluation indicators for the 48 testing images were estimated (Fig. 3). In Fig. 3, the color columns and black lines represent the means and standard deviations, respectively. Additionally, the color difference in the columns shows the various segmentation algorithms. Except for the Panicle-SEG algorithm, the other three contrast algorithm’s standard deviations are relatively large, which reflects their weak adaptability to different field testing images. In addition, for the Panicle-SEG algorithm, the average of Qseg is about 0.626, which is significantly higher than other three contrast algorithms. So, the proposed algorithm has better consistency of both panicle foreground part and background part. And the mean value of SSIM for the Panicle-SEG algorithm is higher than that of the other three contrast algorithms. Moreover, the F-measure is a comprehensive indicator, and it accounts for Precision and Recall; it can achieve 0.767 using our Panicle-SEG algorithm compared with 0.398, 0.441, and 0.209 for HSeg, i2 hysteresis thresholding, and jointSeg, respectively. This phenomenon shows that the Panicle-SEG algorithm could accurately segment rice panicles and guarantee the integrity of panicle segmentation. The comparison results of the testing set for the HSeg, i2 hysteresis thresholding, and jointSeg algorithms are shown in Additional file 4: Table S2.

Four representative testing images were selected to compare the segmented results obtained from different approaches (Fig. 4). In the third column of Fig. 4, the HSeg segmentation results are established. Large background pixels existed, and the average of SSIM is only approximately 0.5. In addition, i2 hysteresis thresholding is a novel idea that was originally proposed for indoor potted side-view rice images segmentation. The color information in the i2 plane and artificial neural network (ANN) modeling were considered for panicle segmentation. The fourth column in Fig. 4 shows the segmentation results. Additionally, jointSeg was originally proposed for maize tassel segmentation in the field. Thus, we retrain the model using 684 training images with default parameters for a fair comparison, and the fifth column in Fig. 4 shows the result. Compared with the HSeg and jointSeg algorithms, the algorithm based on i2 hysteresis thresholding is good at edge-preserving, which means that the edge of rice panicle can be well separated and kept. And the dividing lines fit the edge of the rice panicle well. Moreover, the background using i2 hysteresis thresholding is obviously cleaner. However, because of the dependence on the panicle color, it can hardly resolve white rice panicles caused by cloudy weather. The last column in Fig. 4 is our Panicle-SEG segmentation result. In such a complex field environment, the method we proposed still has a stronger ability for panicle segmentation accuracy. The edges and the structural integrity of the rice panicles maintain are well maintained. Above all, in our previous work, the i2 method [12], which relies on the color and position information, is proposed to extract panicle region. While, the proposed Panicle-SEG algorithm is independent of the color and position information (panicles usually locate at the upper part of the rice plant), which gives it stronger robustness.

## Discussion

### Segmentation results under different rice accessions or illumination environments

**Fig. 5:**
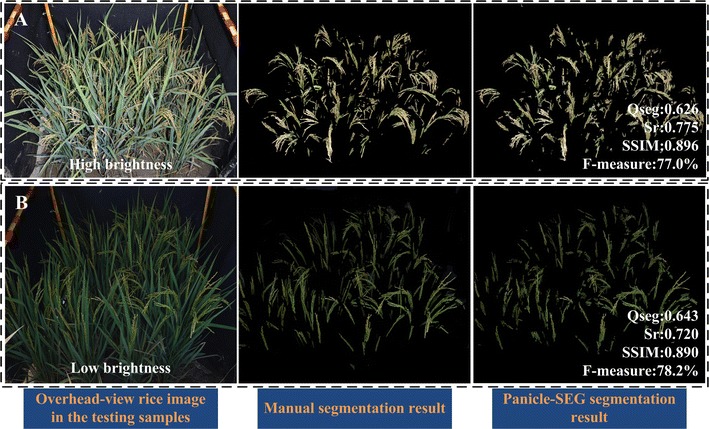
Two field rice images having different illumination. **A** High brightness. **B** Low brightness

Because of the differences in the rice accessions, the panicles’ textures and shapes could be quite different. In Fig. 4A, the upright panicles are partially hidden in the rice leaf blades. For Fig. 4B, the bend growth panicles are basically exposed above the rice leaf blades. At the same time, the panicle awn exists in Fig. 4C. The last column shows the rice panicle segmentation result using the Panicle-SEG algorithm. In each of these three scenarios, compared with the other three segmentation algorithms, the Panicle-SEG algorithm has a stronger ability to perform rice panicle segmentation, and panicle integrity is well maintained. Thus, the proposed Panicle-SEG algorithm is well suited to panicle segmentation with varying rice accessions (totally, 71 rice accessions are used for training and testing processing). Moreover, the weather, as an important field factor, influences the performance of the panicle segmentation result. In Fig. 4C, the illumination intensity in the right image part has a significant difference from that of the left image part, which will cause the situation that pixels could appear in different colors. The highlighted panicle parts look pale. On the other hand, caused by a cloudy day, the panicle color in Fig. 4D appears gray. This type of issue can still be addressed by the proposed Panicle-SEG algorithm. Additionally, the illumination differences (Fig. 5) and illumination unevenness (Additional file 5: Figure S1) are common phenomena in the field environment. Surely, the brightness in Fig. 5A is significantly brighter than Fig. 5B. But the value of SSIM for the two segmented images can reach more than 0.890, which reflects that the segmented images and the manual segmentation results have a high degree of similarity. And, the average of Qseg of these two brightness conditions is over 0.62. So, the proposed algorithm has better consistency of both panicle foreground part and background part even in different illumination conditions. Furthermore, the difference in illumination is not only in different field plot images, but also in different areas of the same image like Additional file 5: Figure S1. In this way, the proposed Panicle-SEG algorithm can still solve this problem well. So, both highlighted regions and gray panicles can be well segmented. This algorithm does not rely on the weather, illumination, shape deformation, and rice accessions, which illustrates strong robustness and stability.

### Segmentation results under different camera angles and different reproductive stages

**Fig. 6:**
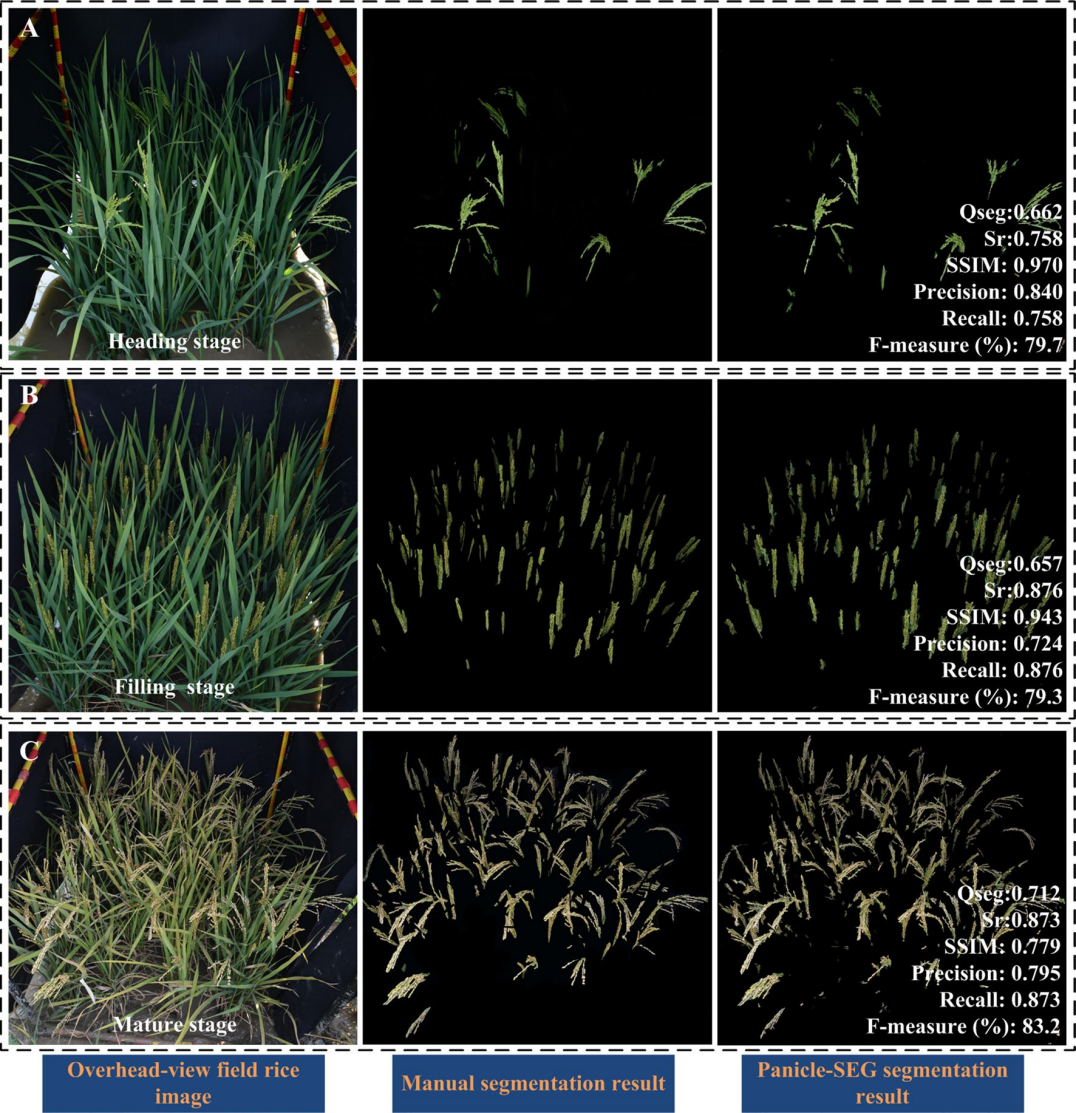
Panicle segmentation results under overhead-view camera angle with different reproductive stages. **A** Heading stage. **B** Filling stage. **C** Mature stage

In the field environment, the overhead-view is also commonly used for field rice image acquisition. Figure 6A–C show three overhead-view field rice images with different reproductive stages (heading stage, filling stage and mature stage). The second column in Fig. 6A–C represents the manual segmentation results using PhotoShop software. And the last column reflects the corresponding Panicle-SEG segmentation result. For heading stage in Fig. 6A, the Qseg, Sr, SSIM, Precision, Recall, and the F-measure (%) can achieve 0.662, 0.758, 0.970, 0.840, 0.758, and 79.7%, respectively. For filling stage in Fig. 6B, the Qseg, Sr, SSIM, Precision, Recall, and the F-measure (%) are 0.657, 0.876, 0.943, 0.724, 0.876 and 79.3%, respectively. And for mature stage in Fig. 6C, the Qseg, Sr, SSIM, Precision, Recall, and the F-measure (%) are 0.712, 0.873, 0.779, 0.795, 0.873 and 83.2%, respectively. In this way, the proposed Panicle-SEG segmentation algorithm can not only adapt to different camera angles, but also has good segmentation result for different growth periods. This allows us to use this algorithm to perform more work such as growth period detection, etc.

### Segmentation results under an indoor inspection environment

**Fig. 7:**
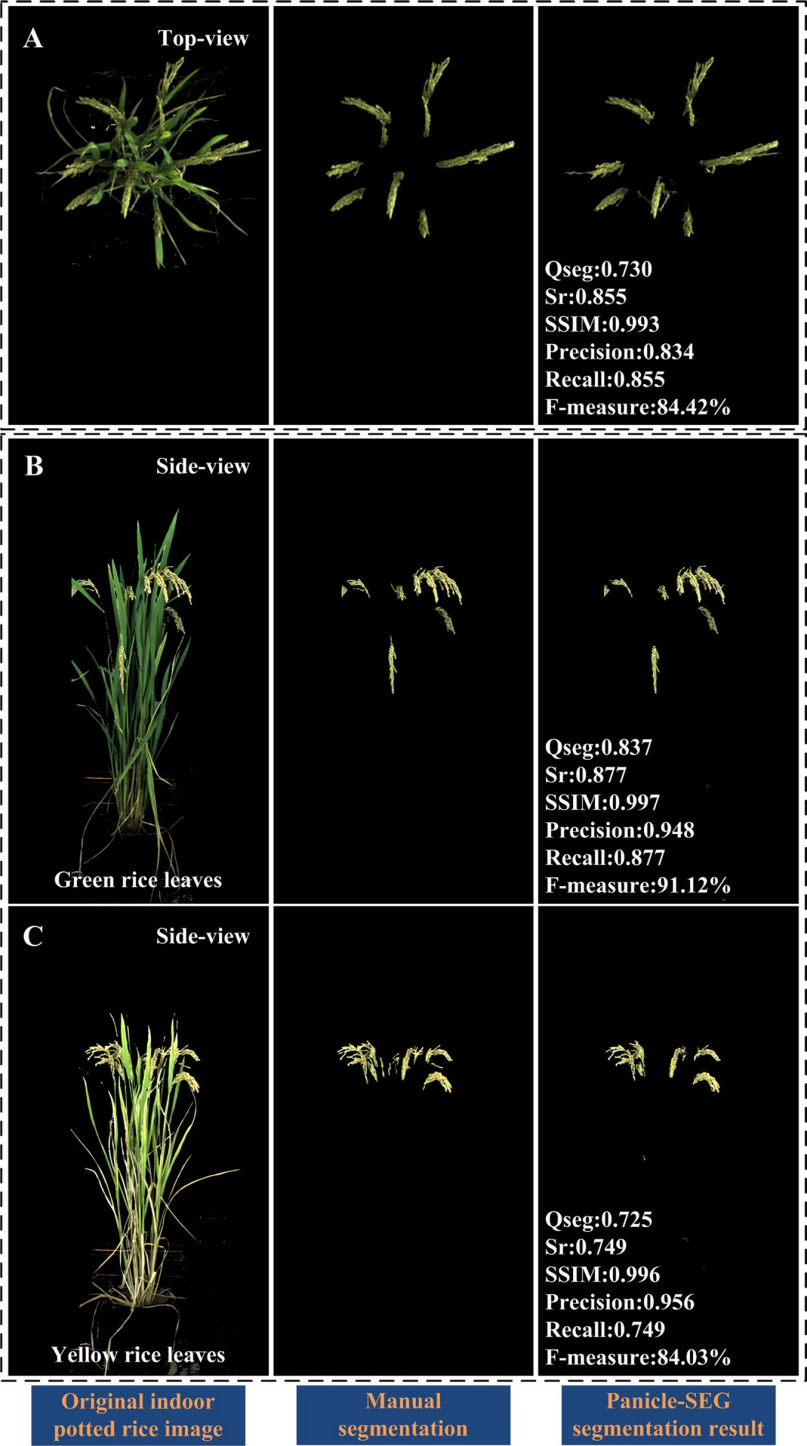
Panicle segmentation results for indoor pot-grown rice images. **A** Indoor top-view rice images. **B** Indoor side-view rice image with green leaves. **C** Indoor side-view rice image with yellow leaves

As a highly-robust panicle segmentation algorithm, the indoor potted rice images acquired by the RGB camera from top-view and side-view are also suitable for panicle segmentation using Panicle-SEG. The original indoor rice images, manually segmented images, and Panicle-SEG segmented results are shown in the left column, center column, and right column of Fig. 7, respectively. As illustrated in Fig. 7, the F-measure (%) is above 84%, the Qseg is above 0.7 and the SSIM values are all above 0.99. Thus, the proposed Panicle-SEG algorithm also has strong flexibility for indoor top-view rice images (Fig. 7A), indoor side-view rice images with green leaves (Fig. 7B), and indoor side-view rice images with yellow leaves (Fig. 7C).

## Conclusion

In this study, we establish a robust and open image segmentation software for segmenting rice panicle based on deep learning and superpixel optimization. Compared with other approaches, the Panicle-SEG algorithm has better performance on segmentation accuracy. Moreover, the Panicle-SEG can be expanded for different field environments, different camera angles, different reproductive stages, and even indoor rice images. Surly, accurate panicle segmentation is the first step and prerequisite for extracting image-based rice panicle traits. After the segmentation, many traits can be obtained for breeding and phenotyping, such as: Growth period detection (like heading period detection) [15], panicle development (area change, color change, maturity test) [23], yield estimation [24] and so on. This study provided a robust image segmentation method for rice panicles in the field, which would potentially facilitate rice breeding or rice phenotyping in future.

## Methods

### Experimental materials and field-based image acquisition

In this study, the experimental paddy field with a total area of 1200 m^2^ is located in Wuhan, Hubei province, China (30.5N, 114.3E). Rice (O. sativa) seeds were sown and germinated during the summer of 2016. The field plot farming method was explained as followed. Each field plot (90 × 90 cm^2^) has 20 rice plants of the same variety, which are planted in five rows and four columns. Considered the edge effect, a guard row of rice plants was planted on the boundary between two adjacent plots. In total, 71 rice accessions were used for training and testing processing in this work. For each plot, two images (top-view and overhead-view, respectively) were extracted. The imaging bracket (Additional file 6: Figure S2) was used to obtain rice plot images. Two cameras (Nikon D40 camera hosting a 23.7 × 15.6 mm CCD matrix, 35 mm focal length lens, 3008 × 2000 pixels and Nikon D7100 camera hosting a 23.5 × 15.6 mm CMOS matrix, 17 mm focal length lens, 4000 × 6000 pixels), including top-view and overhead-view camera were mounted at the top and the side of the imaging bracket, respectively. Wireless shutter was used to trigger the cameras to take images when the imaging bracket moving manually in the paddy field.

### Generation of patches

For classification problems, the traditional machine learning algorithms, such as ANN, must acquire hand-crafted features. However, these hand-crafted features are not guaranteed to provide the subsequent learning algorithm with the optimal description of the data. At the same time, it is difficult to extract satisfactory features for complicated situations. CNN is very similar to ANN (they are composed of neurons that have learnable weights and biases), except for the difference that the inputs of the CNN are images, which means that the CNN has the ability to learn features independently instead of extracting features manually. Since the input image sizes of the CNN should be consistent, the first step is to generate training patches with the same size.

**Fig. 8:**
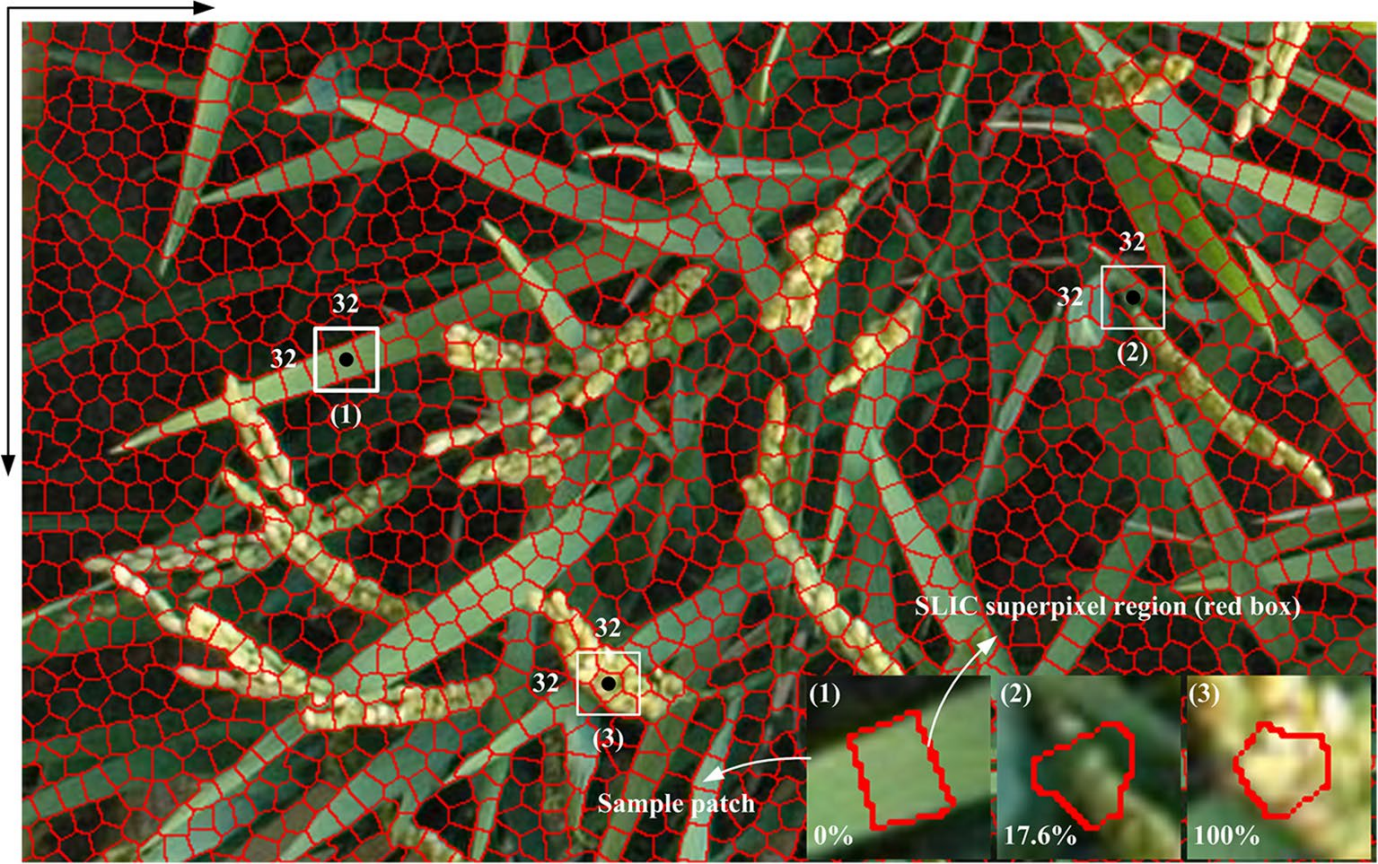
Patch generation approaches based on SLIC. The black point represents the weighted center of the current SLIC superpixel region (irregularly shaped red regions). The region centered on the black point and enclosed by a white box (32 × 32 pixels) is the training patch on the corresponding SLIC superpixel region. (1), (2), and (3) are the zoomed-in versions of the white box training patches, and the percentage is the ratio between the foreground panicle regions to the corresponding SLIC superpixel region in each patch

The pixel-level segmentation approaches have achieved a moderate degree of success. At the same time, ignoring the neighborhood information will have a serious impact on the edge-preserving ability of the segmentation algorithm. Thus, the idea of processing the image patches with similar characteristics instead of single pixels has contributed to overcoming the influence of noise, accelerating the processing speed, and improving the panicle edge-preserving ability. Moreover, the input of the CNN requires a unified size of the images. To achieve this goal, SLIC was applied to extract superpixel image patches. The clustering algorithm with color information in the LAB color space and position coordinate were adopted to generate superpixel regions. At the same time, the superpixel regions with fundamental coincident size and shape also meet the requirements of generating uniform patches. SLIC algorithm has two parameters: K and M. K is the number of superpixel, and M is superpixel compact degree. In our paper, the compact coefficient (M) is set to 10 and remains unchanged for all the samples (training set and testing set). In the training process, the number of superpixel (K) is set to 20,000 for the training images with a resolution of 1815 × 1971 pixels. For different size of input images in the testing process, we expect the area of each superpixel regions to be basically consistent. So, the fixed proportional relation (1815 × 1971/20,000) was used to calculate the number of superpixel (K) for a new testing sample. The red irregular polygons in Fig. 8 represent SLIC superpixel regions. Compared to the other superpixel generation algorithm, the computing speed is faster than the others, and the algorithm has a good ability for edge preservation. However, the shape of these SLIC superpixel regions is irregular, and thus, they cannot be directly used as CNN input. Therefore, a small window called a patch (32 × 32 pixels), which is centered on the weighted centre of the current SLIC superpixel region, is given to the CNN. Detailed processing for patches generation using SLIC method has been described in Additional file 7: Appendix S2.

### Automatic labeling

CNN offline training is a supervised process, which means that the input patches and their corresponding labels are both needed. Due to the limitation of having enormous numbers of samples, it is unrealistic to label each patch manually. Thus, a rapid and accurate labeling method was proposed in this work. Two images are needed. The first image is an original field rice image (Fig. 2A), and the second image is the corresponding mask image with a high degree of segmentation accuracy (Fig. 2B), which is obtained manually using PhotoShop software. The white part in the mask image represents the foreground area of the rice panicle. Firstly, SLIC superpixel regions were generated as discussed in the previous section, such as the red irregular polygons in Fig. 8. The black point represents the weighted center of the SLIC superpixel region. The region centered on the black point and enclosed by a white box (32 × 32 pixels) is the sample patch on the corresponding SLIC superpixel region. The boxes (1), (2), and (3) in Fig. 8 are the zoom-in versions of the white box training patches, and the percentages in each patch represent the ratio between the foreground panicle areas (the white part in the mask image) to the corresponding areas of the SLIC superpixel region. The sample patch is labeled category zero (confirmed background) if the percentage of the current patch is equal to zero; otherwise, it is labeled category one (candidate for panicle). The advantage of this labeling method is that it guarantees that all of the categories zero samples do not contain foreground panicle pixels, which can improve the classification accuracy of the CNN. However, as long as the patch has foreground panicle pixels, regardless of how many it has, the labeling method will still tag it to a candidate panicle patch, which means that the coarse segmentation result is likely to contain background pixels. Thus, the optimization processing is needed.

### Training set and validation set building

Given the patch labeling proposals, the next question is how to build a training set and validation set. Here, 684 representative rice images are selected as the total sample set, in which the water reflection, illumination unbalance, cluttered background, rice accession, weather conditions, and reproductive stage are all considered (Fig. 1). For the progress of the sample patch generation, the number of category zero (negative) samples is far larger than the number of category one (positive) samples. Thus, sample balance is necessary. All of the positive samples are chosen as a sample set. For the negative samples, the Gaussian mixture model (GMM) [25] is applied for unsupervised clustering. The mean value and standard deviation of each of the training patches in RGB color space are extracted as the input vector of GMM. In total, 9 categories are obtained, and equivalent samples are selected randomly from each category to join the sample set. Considering the complications of the field-based environment, the next step is to augment the dataset. Thus, to simulate the illumination change, the intensity component of the image in HSV color space is adjusted. At the same time, the wind influence and image defocus phenomenon is also common in the field-based imaging environment, which will cause image blur condition. Gaussian blur with 3 × 3 smoothing Gaussian kernel is adopted to simulate this situation. These augmented images are all appended to the sample set, from which twenty percent of the sample set is randomly selected as the validation set (225,472 patches), and the remaining eighty percent is selected as the training set (901,895 patches) (Additional file 8: Figure S3). Detailed processing and parameters for the samples set building and data augmentation have been described in Additional file 7: Appendix S2.

### CNN training and Panicle-SEG-CNN model generation

**Fig. 9:**
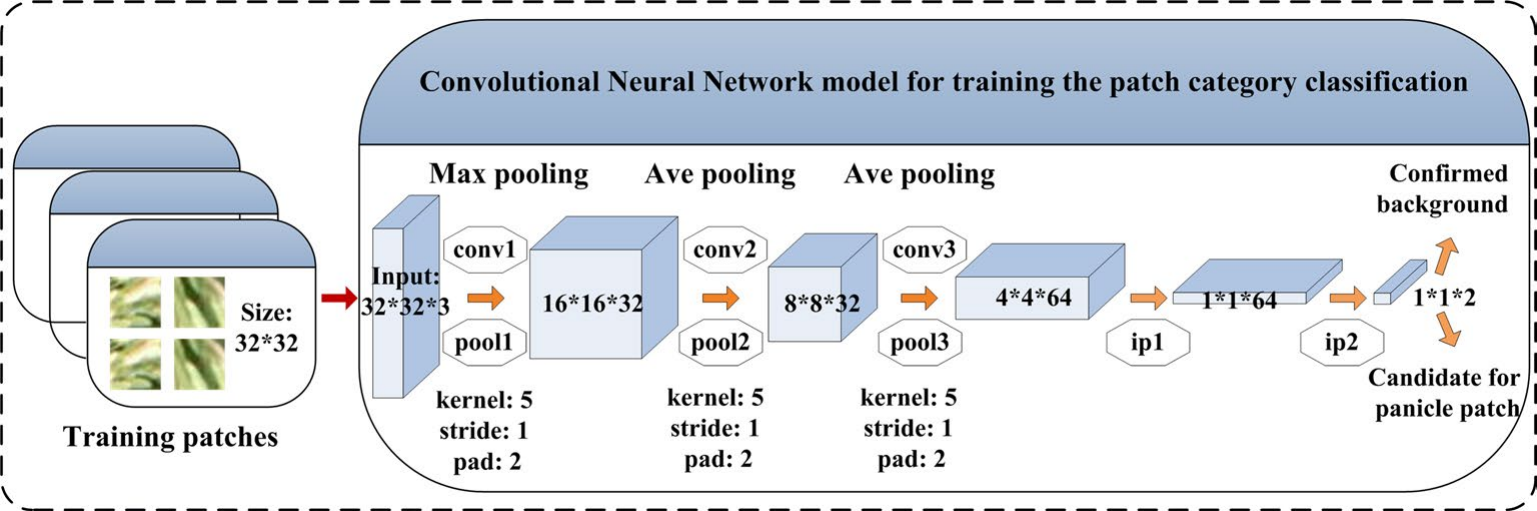
Convolutional neural network model for training the patch category classification. The primary architecture of the network is composed of 5 layers (3 convolution layers and 2 fully connected layers)

Caffe, an open source deep learning framework, was used in this work [26]. The detailed software implementation strategy using Caffe is discussed at Additional file 7: Appendix S2. Considering that the input size of the color patches is 32 × 32 pixels, which is similar to the cifar10 dataset, a CNN network developed for the cifar10 dataset was applied for our rice patch classification. Figure 9 shows the architecture of the CNN network, which is composed of 5 layers (3 convolution layers and 2 fully connected layers). The detailed hyper-parameters for each layer in our CNN network can refer to Additional file 9: Figure S4. In this way, the CNN transforms the original patch layer by layer from the original pixel values to the final class scores.

When the training set is ready, the first step is data preprocessing. One necessary process is to subtract the mean value over the training set from each pixel. Then, the CNN is trained by iteratively passing the training images and adjusting the network weights and biases based on the classification accuracy in the validation set. Additionally, the entire update process is based on the stochastic gradient descent (SGD) with momentum algorithm. The start of the base learning rate is 0.001. Then, we decreased the learning rate by a factor of 0.3 for 20,000 iterations, 100,000 iterations, 160,000 iterations, 240,000 iterations and 320,000 iterations. At the same time, we set the start value of the momentum to 0.9, which remained unchanged for all the training processing. And the maximum number of iterations is set to 600,000. The final training model (Panicle-SEG-CNN) is saved to disk according to the Google Protocol Buffers, which can be called by using the Caffe C++ interface in the testing process. Detailed training processing has been described in Additional file 7: Appendix S2.

### Coarse segmentation by using the pre-trained Panicle-SEG-CNN model

For a testing rice image, the first operation is the testing patches generation. The SLIC superpixel algorithm is applied to generate the testing patches. Then, we ran each test patch through the pre-trained Panicle-SEG-CNN model, to obtain the category of each patch. The SLIC superpixel region, which corresponds to the positive testing patch, will be retained as the candidate rice panicle region. In turn, the SLIC superpixel region, which corresponds to the negative testing patch, will be removed as a confirmed background. The coarse segmentation result will be joined together by combining the candidate panicle patches. As discussed in the Automatic labeling section, the coarse segmentation result is likely to contain some background pixels. At the same time, the CNN classification cannot ensure that all of the testing patches were classified correctly. Thus, the optimization algorithm is needed.

### Entropy rate superpixel optimization

**Fig. 10:**
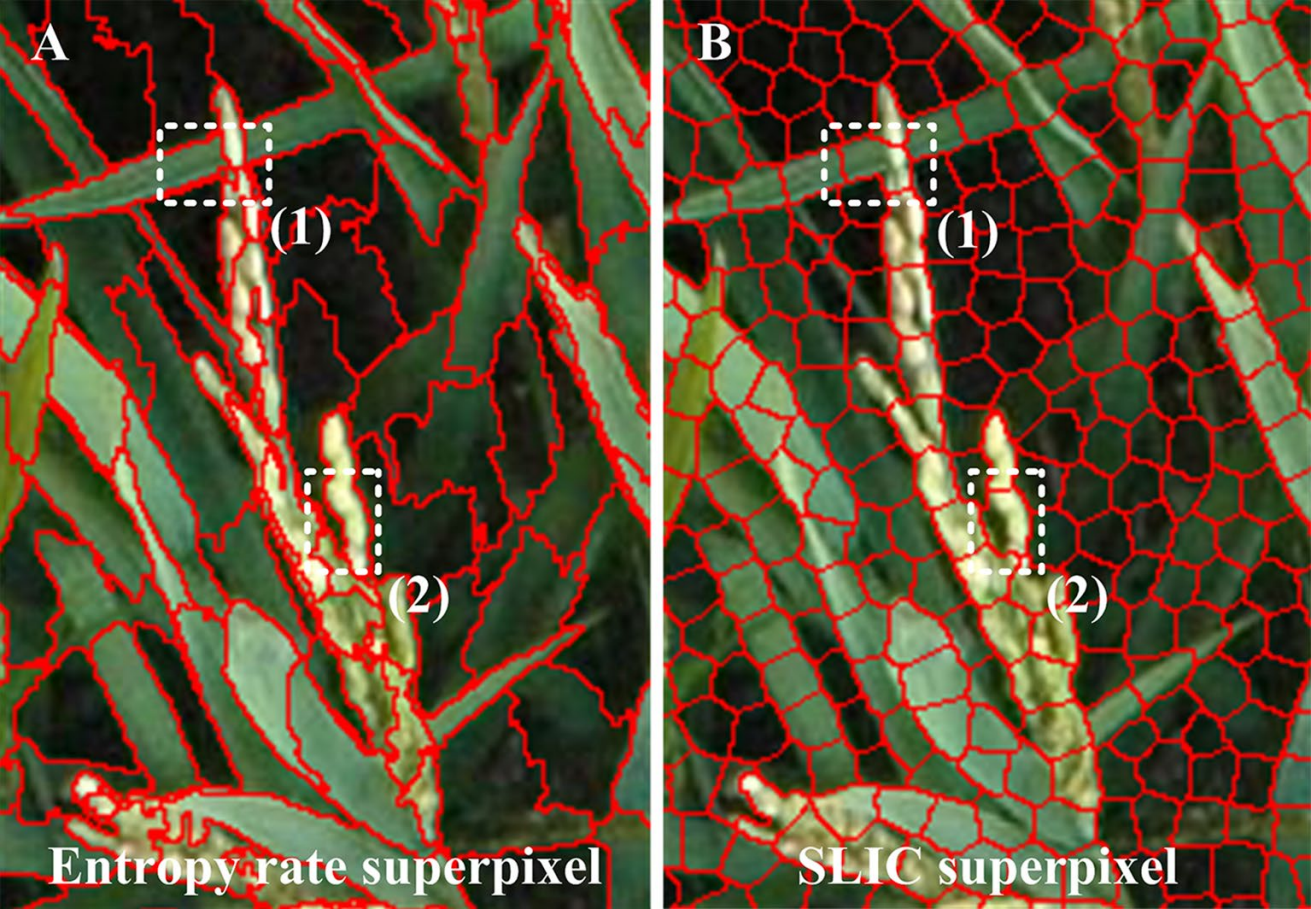
The principle of entropy rate superpixel optimization. **A** Entropy rate superpixel image. The typical characteristic of the entropy rate superpixel image is having larger background regions and relatively small foreground panicle regions, and the algorithm has stronger abilities in edge preservation. **B** SLIC superpixel image. The edge of the SLIC superpixel region is relatively regulated. Sometimes, the foreground pixels and background pixels can exist in the same SLIC superpixel region, such as in the white dotted boxes (1) and (2) dpi

Entropy rate superpixel segmentation is another superpixel segmentation algorithm. Figure 10A shows the entropy rate superpixel image, which has 5000 superpixel regions. Figure 10B reflects the corresponding SLIC superpixel image, which has 20,000 superpixel regions. Both algorithms have the ability to accomplish boundary adherence. At the same time, the entropy rate superpixel algorithm with fewer superpixel regions can achieve a beneficial effect on the edge preservation. Meantime, the entropy rate superpixel image has larger background regions and relatively smaller foreground panicle regions, which provides an advantage for segmentation optimization. So, a relatively small entropy rate superpixel region, around 5000 (for image with resolution of 1815 × 1971 pixels in training processing), is a good option after experimental analysis (can refer to Additional file 7: Appendix S2). For different size of input images in the testing process, the fixed proportional relation (1815 × 1971/5000) was used to calculate the number of entropy rate superpixel region for a new testing sample. The process for entropy rate superpixel optimization is as follows: First, we assume that the coarse segmentation is the correct segmentation result. In this place, all of the pixels in the candidate panicle regions at the coarse segmentation result were marked as a gray value of 255 (panicle pixels), and the pixels in the confirmed background regions were marked as a gray value of 0 (background pixels). Then, the number of panicle pixels and background pixels within each entropy rate superpixel region are calculated. The entropy rate superpixel region was considered to be the final rice panicle region if the ratio of the panicle pixel number to the total pixel number in each entropy rate superpixel region is greater than the optimization parameter. Here, a satisfactory result can be obtained by using the optimization parameter 0.9 in this paper. The detailed optimization parameter discussion can refer to Additional file 7: Appendix S2. Subsequently, small regions with an area smaller than a predefined area threshold (500 pixels in this study) were removed.

The proposed entropy rate superpixel segmentation, as a highly efficient and strongly feasible optimization algorithm, has unique advantages in the following respects. First, errors could occur in the CNN classification in the coarse segmentation result. Some background SLIC superpixel regions could be classified to candidate panicle regions. Commonly, relative to the large entropy rate superpixel background region, the area of error of the SLIC superpixel region accounts for a relatively small proportion. Thus, after the optimization, the segmentation noise caused by error classification can be significantly suppressed. Second, because the edges of the SLIC superpixel regions are relatively well-regulated, the foreground pixels and background pixels could exist in the same SLIC superpixel region, similar to the white dotted boxes (1) and (2) in Fig. 10. For example, suppose that these regions are classified into candidate panicle regions, which will lead to poor edge segmentation results in the coarse result. In contrast, entropy rate superpixel segmentation has a stronger ability for edge preservation, which will retain the accuracy and the completeness of the edge extraction after the optimization algorithm.

In ERS optimization, there are three parameters (the number of entropy rate superpixel regions, balancing parameter, and optimization parameter) that need to be predefined. Detailed parameters selection can refer to Additional file 7: Appendix S2.

### Speed up the segmentation project

In this study, the whole segmentation project is developed in C++ using the OpenCV library [27]. Additionally, the Caffe framework for CNN classification was encapsulated in the project by calling the C++ interface. The OpenMP, as an application programming interface that supports multi-platform shared memory in C++, was applied to increase the speed of the project. The performance of the acceleration depends on the CPU frequency and the number of CPU cores. At the same time, Caffe’s integration with CUDA and the cuDNN library accelerates Caffe processing models. CUDA is a parallel computing platform that was created by NVIDIA, and the cuDNN library was developed for deep learning with GPU acceleration. The uses of high-performance GPUs have changed the traditional opinions about acceleration and greatly shorten the processing time of the segmentation algorithm. Here, the parallel computing using GPU is adopted in the CNN classification. In this way, the time for the rice panicle segmentation can achieve approximately 70 s per image, with a resolution of 1815 × 1971 pixels. Of course, the current segmentation project has the potential to be faster in the future. For example, the GPU acceleration can be applied in the process of entropy rate superpixel segmentation.

## Additional files

**Additional file 1: Video S1.** The instruction of the rice panicle segmentation software.mp4.

**Additional file 2: Appendix S1.** The instruction of the Panicle-SEG segmentation software.

**Additional file 3: Table S1.** The evaluation criterion for the 48 testing rice samples.

**Additional file 4: Table S2.** The evaluation criterion for 48 testing rice samples using four different segmentation algorithms.

**Additional file 5: Figure S1.** Different regions in the field plot image have different illumination condition.

**Additional file 6: Figure S2.** Imaging bracket with two cameras (top-view and overhead-view).

**Additional file 7: Appendix S2.** Panicle-SEG technical documentation.

**Additional file 8. Figure S3.** Data augmentation and sample set building.

**Additional file 9: Figure S4.** The CNN architectures applied in Panicle-SEG algorithm.

## Abbreviations

CNN: convolutional neural network
ERS: entropy rate superpixel
SIFT: scale-invariant feature transform
SVM: support vector machine
CUDA: compute unified device architecture
SLIC: simple linear iterative clustering
CPU: central processing unit
GPU: graphics processing unit
SSIM: Structural Similarity Index
TP: true positives
TN: true negatives
FP: false positives
FN: false negatives
ANN: artificial neural network
GMM: Gaussian mixture model
SGD: stochastic gradient descent
Caffe: convolutional architecture for fast feature embedding
cuDNN: NVIDIA CUDA Deep Neural Network library
