# Supplementary material for: Panicle-SEG: a robust image segmentation method for rice panicles in the field based on deep learning and superpixel optimization
Source: Plant Methods. 2017 Nov 28;13:104. doi: 10.1186/s13007-017-0254-7 (PMC5704426; doi:10.1186/s13007-017-0254-7)
Supplement: Supplementary file 2 — Additional file 2: Appendix S1. The instruction of the Panicle-SEG segmentation software. [file 13007_2017_254_MOESM2_ESM.docx]

**The instruction of the Panicle-SEG segmentation software**

The proposed project name is "Panicle-SEG", which is designed for rice panicle segmentation. Since there is no interface, the whole project needs to run in command line mode. And the software has been tested on 64-bit Windows 7 system and 64-bit Windows 10 system in the CPU and GPU mode. If you want to download the testing samples and installable file, you can click the web link below:

*http://plantphenomics.hzau.edu.cn/checkiflogin_en.action* (username: UserPP; password: 20170108pp)

This software can only be used for academic purpose. For any problem on this software, please contact with author, the email address is: *971123916@qq.com*.

How to use the software can refer to the **Additional File 1: Video S1**. And the detailed software implementation procedure as follows: (1) Download the new version of Panicle-SEG installable file, you can choose CPU or GPU mode. (2) Install the "setup_Panicle-SEG_cpu.exe" if you choose the CPU mode or "setup_Panicle-SEG_gpu.exe" file if you choose the GPU mode. (3) Open the command line in your computer and enter to the current file path. (4) Open the "Readme.txt" and revise the parameters if needed. The detailed explanations for the parameters were shown below. (5) Copy the revised content in "Readme.txt" and paste to the command line. (6) Waiting for rice panicle segmentation (the cost time depends on your computer performance). (6) The segmentation result is saved in the "segmentation_results" file. In step (4), an example format for Panicle-SEG software in command line:

***Panicle-SEG images_test/top_view_field.jpg 4 deploy1.prototxt cifar10_quick_iter_600000.caffemodel mean_leveldb_32.binaryproto label_filename.txt 0.5 0.9 500 segmentation_results/segmentation_top.jpg***

The detailed explanation for command line parameters as follows:

(1) Executable file name: ***Panicle-SEG*** (retain the default settings).

(2) Input rice image path and name: ***images_test/***.jpg*** (Defaults to current path if not specified).

(3) The number of CPU cores: ***4*** (revise the parameters if needed).

(4) The default CNN configuration files: (retain the default settings):

***deploy1.prototxt, cifar10_quick_iter_400000.caffemodel, mean_leveldb_32.binaryproto, label_filename.txt***

(5) Balancing parameter (also called lambda): ***0.5*** (revise the parameters if needed and the range of it is from 0 to 1). The detailed description of the balancing parameter can refer to Additional File 7: Appendix S2.

(6) Optimization coefficient: ***0.9*** (revise the parameters if needed and the range of it is from 0 to 1). The detailed description of the balancing parameter can refer to Additional File 7: Appendix S2.

(7) The area threshold for removing small background regions: ***500*** pixel^2^ (revise the parameters if needed)

(8) The save path of the final segmentation result: ***segmentation_results/***.jpg*** (Defaults to current path if not specified)

We test some different size of testing images in the GPU mode, the results are shown below: (1) the time for an overhead view field image with resolution of 3904×3693 pixels is about 8 minutes. (2) The time for a top view field image with resolution of 1815×1971 pixels is about 70 seconds. (3) The time for a side view indoor image with resolution of 750×1580 pixels is about 18 seconds. (4) The time for a top view indoor image with resolution of 462×398 pixels is about 2.2 seconds. The evaluation results reflect that the Panicle-SEG algorithm can address any size for the original input rice image. At the same time, with a decreased size of the input image to be processed, the time required will obviously be reduced.
