# Supplementary material for: Panicle-SEG: a robust image segmentation method for rice panicles in the field based on deep learning and superpixel optimization
Source: Plant Methods. 2017 Nov 28;13:104. doi: 10.1186/s13007-017-0254-7 (PMC5704426; doi:10.1186/s13007-017-0254-7)
Supplement: Supplementary file 3 — Additional file 3: Table S1. The evaluation criterion for the 48 testing rice samples. [file 13007_2017_254_MOESM3_ESM.docx]

**Table S1. The evaluation criterion for the 48 testing rice samples.**

| **Number** | **Qseg** | **Sr** | **SSIM** | **Precision** | **Recall** | **F-measure** |
| --- | --- | --- | --- | --- | --- | --- |
| **1** | 0.563 | 0.655 | 0.853 | 0.800 | 0.655 | 0.721 |
| **2** | 0.589 | 0.706 | 0.821 | 0.780 | 0.706 | 0.741 |
| **3** | 0.666 | 0.742 | 0.828 | 0.866 | 0.742 | 0.800 |
| **4** | 0.489 | 0.661 | 0.904 | 0.652 | 0.661 | 0.657 |
| **5** | 0.540 | 0.641 | 0.855 | 0.775 | 0.641 | 0.702 |
| **6** | 0.587 | 0.726 | 0.859 | 0.754 | 0.726 | 0.740 |
| **7** | 0.611 | 0.707 | 0.847 | 0.819 | 0.707 | 0.759 |
| **8** | 0.589 | 0.721 | 0.838 | 0.763 | 0.721 | 0.741 |
| **9** | 0.601 | 0.694 | 0.860 | 0.817 | 0.694 | 0.751 |
| **10** | 0.696 | 0.751 | 0.783 | 0.905 | 0.751 | 0.821 |
| **11** | 0.485 | 0.559 | 0.879 | 0.785 | 0.559 | 0.653 |
| **12** | 0.618 | 0.687 | 0.844 | 0.860 | 0.687 | 0.764 |
| **13** | 0.595 | 0.706 | 0.861 | 0.791 | 0.706 | 0.746 |
| **14** | 0.631 | 0.743 | 0.882 | 0.808 | 0.743 | 0.774 |
| **15** | 0.580 | 0.640 | 0.756 | 0.862 | 0.640 | 0.734 |
| **16** | 0.489 | 0.535 | 0.819 | 0.850 | 0.535 | 0.657 |
| **17** | 0.551 | 0.587 | 0.820 | 0.901 | 0.587 | 0.711 |
| **18** | 0.604 | 0.645 | 0.807 | 0.905 | 0.645 | 0.753 |
| **19** | 0.676 | 0.746 | 0.887 | 0.877 | 0.746 | 0.806 |
| **20** | 0.693 | 0.748 | 0.849 | 0.903 | 0.748 | 0.819 |
| **21** | 0.555 | 0.612 | 0.936 | 0.855 | 0.612 | 0.713 |
| **22** | 0.675 | 0.756 | 0.855 | 0.863 | 0.756 | 0.806 |
| **23** | 0.526 | 0.681 | 0.925 | 0.699 | 0.681 | 0.690 |
| **24** | 0.482 | 0.548 | 0.899 | 0.800 | 0.548 | 0.651 |
| **25** | 0.644 | 0.874 | 0.858 | 0.735 | 0.837 | 0.783 |
| **26** | 0.635 | 0.814 | 0.864 | 0.742 | 0.814 | 0.777 |
| **27** | 0.640 | 0.731 | 0.894 | 0.836 | 0.731 | 0.780 |
| **28** | 0.712 | 0.873 | 0.779 | 0.795 | 0.873 | 0.832 |
| **29** | 0.648 | 0.763 | 0.520 | 0.811 | 0.763 | 0.786 |
| **30** | 0.657 | 0.876 | 0.943 | 0.724 | 0.876 | 0.793 |
| **31** | 0.626 | 0.775 | 0.896 | 0.765 | 0.775 | 0.770 |
| **32** | 0.643 | 0.720 | 0.889 | 0.857 | 0.720 | 0.782 |
| **33** | 0.601 | 0.696 | 0.976 | 0.814 | 0.696 | 0.750 |
| **34** | 0.662 | 0.758 | 0.970 | 0.840 | 0.758 | 0.797 |
| **35** | 0.690 | 0.809 | 0.934 | 0.825 | 0.809 | 0.817 |
| **36** | 0.675 | 0.803 | 0.966 | 0.809 | 0.803 | 0.806 |
| **37** | 0.583 | 0.641 | 0.990 | 0.864 | 0.641 | 0.736 |
| **38** | 0.713 | 0.796 | 0.989 | 0.872 | 0.796 | 0.832 |
| **39** | 0.729 | 0.821 | 0.986 | 0.866 | 0.821 | 0.843 |
| **40** | 0.633 | 0.673 | 0.986 | 0.914 | 0.673 | 0.775 |
| **41** | 0.635 | 0.647 | 0.986 | 0.971 | 0.647 | 0.777 |
| **42** | 0.723 | 0.750 | 0.979 | 0.952 | 0.750 | 0.839 |
| **43** | 0.840 | 0.878 | 0.986 | 0.950 | 0.878 | 0.913 |
| **44** | 0.619 | 0.797 | 0.963 | 0.735 | 0.797 | 0.765 |
| **45** | 0.603 | 0.894 | 0.992 | 0.650 | 0.894 | 0.752 |
| **46** | 0.681 | 0.763 | 0.994 | 0.863 | 0.763 | 0.810 |
| **47** | 0.614 | 0.852 | 0.988 | 0.687 | 0.852 | 0.761 |
| **48** | 0.730 | 0.855 | 0.993 | 0.834 | 0.855 | 0.844 |
| **Mean** | **0.626** | **0.730** | **0.891** | **0.821** | **0.730** | **0.7673** |
| **Std^a^** | **0.072** | **0.090** | **0.088** | **0.074** | **0.090** | **0.0546** |

a. The standard deviation value
