# Supplementary material for: Panicle-SEG: a robust image segmentation method for rice panicles in the field based on deep learning and superpixel optimization
Source: Plant Methods. 2017 Nov 28;13:104. doi: 10.1186/s13007-017-0254-7 (PMC5704426; doi:10.1186/s13007-017-0254-7)
Supplement: Supplementary file 4 — Additional file 4: Table S2. The evaluation criterion for 48 testing rice samples using four different segmentation algorithms. [file 13007_2017_254_MOESM4_ESM.docx]

**Table S2. The evaluation criterion for 48 testing rice samples using four different segmentation algorithms**

| **Number** | **Qseg** | | | | **Sr** | | | | **SSIM** | | | | **Precision** | | | | **Recall** | | | | **F-measure** | | | |
| --- | --- | --- | --- | --- | --- | --- | --- | --- | --- | --- | --- | --- | --- | --- | --- | --- | --- | --- | --- | --- | --- | --- | --- | --- |
|  | **Panicle-SEG** | **HSeg** | **I2** | **JointSeg** | **Panicle-SEG** | **HSeg** | **I2** | **JointSeg** | **Panicle-SEG** | **HSeg** | **I2** | **JointSeg** | **Panicle-SEG** | **HSeg** | **I2** | **JointSeg** | **Panicle-SEG** | **HSeg** | **I2** | **JointSeg** | **Panicle-SEG** | **HSeg** | **I2** | **JointSeg** |
| **1** | 0.563 | 0.313 | 0.265 | 0.130 | 0.655 | 0.848 | 0.380 | 0.144 | 0.853 | 0.513 | 0.763 | 0.789 | 0.800 | 0.332 | 0.467 | 0.570 | 0.655 | 0.848 | 0.380 | 0.144 | 0.721 | 0.477 | 0.419 | 0.230 |
| **2** | 0.589 | 0.313 | 0.388 | 0.061 | 0.706 | 0.607 | 0.749 | 0.073 | 0.821 | 0.483 | 0.680 | 0.702 | 0.780 | 0.392 | 0.445 | 0.272 | 0.706 | 0.607 | 0.749 | 0.073 | 0.741 | 0.477 | 0.559 | 0.116 |
| **3** | 0.666 | 0.444 | 0.442 | 0.098 | 0.742 | 0.804 | 0.797 | 0.100 | 0.828 | 0.497 | 0.637 | 0.701 | 0.866 | 0.497 | 0.497 | 0.831 | 0.742 | 0.804 | 0.797 | 0.100 | 0.800 | 0.615 | 0.613 | 0.179 |
| **4** | 0.489 | 0.149 | 0.155 | 0.068 | 0.661 | 0.855 | 0.875 | 0.136 | 0.904 | 0.526 | 0.640 | 0.830 | 0.652 | 0.153 | 0.158 | 0.120 | 0.661 | 0.855 | 0.875 | 0.136 | 0.657 | 0.260 | 0.268 | 0.127 |
| **5** | 0.540 | 0.266 | 0.242 | 0.077 | 0.641 | 0.744 | 0.616 | 0.098 | 0.855 | 0.477 | 0.643 | 0.766 | 0.775 | 0.292 | 0.285 | 0.269 | 0.641 | 0.744 | 0.616 | 0.098 | 0.702 | 0.420 | 0.390 | 0.143 |
| **6** | 0.587 | 0.326 | 0.286 | 0.178 | 0.726 | 0.785 | 0.478 | 0.222 | 0.859 | 0.567 | 0.764 | 0.793 | 0.754 | 0.358 | 0.415 | 0.471 | 0.726 | 0.785 | 0.478 | 0.222 | 0.740 | 0.491 | 0.445 | 0.302 |
| **7** | 0.611 | 0.226 | 0.292 | 0.075 | 0.707 | 0.558 | 0.778 | 0.089 | 0.847 | 0.418 | 0.558 | 0.735 | 0.819 | 0.275 | 0.318 | 0.319 | 0.707 | 0.558 | 0.778 | 0.089 | 0.759 | 0.368 | 0.452 | 0.139 |
| **8** | 0.589 | 0.329 | 0.359 | 0.221 | 0.721 | 0.793 | 0.862 | 0.271 | 0.838 | 0.523 | 0.635 | 0.761 | 0.763 | 0.360 | 0.380 | 0.544 | 0.721 | 0.793 | 0.862 | 0.271 | 0.741 | 0.495 | 0.528 | 0.361 |
| **9** | 0.601 | 0.361 | 0.373 | 0.276 | 0.694 | 0.872 | 0.867 | 0.302 | 0.860 | 0.526 | 0.678 | 0.811 | 0.817 | 0.381 | 0.396 | 0.762 | 0.694 | 0.872 | 0.867 | 0.302 | 0.751 | 0.531 | 0.544 | 0.432 |
| **10** | 0.696 | 0.624 | 0.635 | 0.297 | 0.751 | 0.746 | 0.803 | 0.300 | 0.783 | 0.570 | 0.707 | 0.654 | 0.905 | 0.793 | 0.751 | 0.966 | 0.751 | 0.746 | 0.803 | 0.300 | 0.821 | 0.769 | 0.776 | 0.458 |
| **11** | 0.485 | 0.230 | 0.248 | 0.124 | 0.559 | 0.735 | 0.533 | 0.156 | 0.879 | 0.569 | 0.755 | 0.819 | 0.785 | 0.251 | 0.317 | 0.374 | 0.559 | 0.735 | 0.533 | 0.156 | 0.653 | 0.374 | 0.397 | 0.220 |
| **12** | 0.618 | 0.289 | 0.348 | 0.114 | 0.687 | 0.608 | 0.785 | 0.128 | 0.844 | 0.449 | 0.608 | 0.731 | 0.860 | 0.356 | 0.385 | 0.517 | 0.687 | 0.608 | 0.785 | 0.128 | 0.764 | 0.449 | 0.517 | 0.205 |
| **13** | 0.595 | 0.372 | 0.420 | 0.135 | 0.706 | 0.826 | 0.782 | 0.159 | 0.861 | 0.571 | 0.754 | 0.781 | 0.791 | 0.403 | 0.475 | 0.466 | 0.706 | 0.826 | 0.782 | 0.159 | 0.746 | 0.542 | 0.591 | 0.237 |
| **14** | 0.631 | 0.323 | 0.480 | 0.174 | 0.743 | 0.896 | 0.737 | 0.201 | 0.882 | 0.524 | 0.811 | 0.810 | 0.808 | 0.336 | 0.580 | 0.558 | 0.743 | 0.896 | 0.737 | 0.201 | 0.774 | 0.489 | 0.649 | 0.296 |
| **15** | 0.580 | 0.432 | 0.466 | 0.183 | 0.640 | 0.623 | 0.764 | 0.203 | 0.756 | 0.472 | 0.621 | 0.627 | 0.862 | 0.585 | 0.545 | 0.651 | 0.640 | 0.623 | 0.764 | 0.203 | 0.734 | 0.603 | 0.636 | 0.310 |
| **16** | 0.489 | 0.299 | 0.291 | 0.135 | 0.535 | 0.725 | 0.786 | 0.150 | 0.819 | 0.443 | 0.535 | 0.746 | 0.850 | 0.337 | 0.316 | 0.570 | 0.535 | 0.725 | 0.786 | 0.150 | 0.657 | 0.460 | 0.450 | 0.237 |
| **17** | 0.551 | 0.343 | 0.394 | 0.111 | 0.587 | 0.628 | 0.747 | 0.122 | 0.820 | 0.447 | 0.648 | 0.714 | 0.901 | 0.431 | 0.454 | 0.550 | 0.587 | 0.628 | 0.747 | 0.122 | 0.711 | 0.511 | 0.565 | 0.199 |
| **18** | 0.604 | 0.370 | 0.200 | 0.117 | 0.645 | 0.688 | 0.237 | 0.122 | 0.807 | 0.521 | 0.658 | 0.690 | 0.905 | 0.445 | 0.559 | 0.759 | 0.645 | 0.688 | 0.237 | 0.122 | 0.753 | 0.540 | 0.333 | 0.209 |
| **19** | 0.676 | 0.354 | 0.426 | 0.208 | 0.746 | 0.810 | 0.813 | 0.262 | 0.887 | 0.593 | 0.749 | 0.783 | 0.877 | 0.386 | 0.472 | 0.504 | 0.746 | 0.810 | 0.813 | 0.262 | 0.806 | 0.523 | 0.597 | 0.345 |
| **20** | 0.693 | 0.427 | 0.491 | 0.276 | 0.748 | 0.750 | 0.823 | 0.304 | 0.849 | 0.559 | 0.691 | 0.732 | 0.903 | 0.498 | 0.550 | 0.750 | 0.748 | 0.750 | 0.823 | 0.304 | 0.819 | 0.599 | 0.659 | 0.433 |
| **21** | 0.555 | 0.161 | 0.243 | 0.130 | 0.612 | 0.946 | 0.727 | 0.172 | 0.936 | 0.503 | 0.817 | 0.898 | 0.855 | 0.163 | 0.267 | 0.346 | 0.612 | 0.946 | 0.727 | 0.172 | 0.713 | 0.277 | 0.391 | 0.230 |
| **22** | 0.675 | 0.462 | 0.498 | 0.184 | 0.756 | 0.767 | 0.851 | 0.197 | 0.855 | 0.604 | 0.733 | 0.752 | 0.863 | 0.537 | 0.545 | 0.738 | 0.756 | 0.767 | 0.851 | 0.197 | 0.806 | 0.632 | 0.665 | 0.311 |
| **23** | 0.526 | 0.163 | 0.160 | 0.139 | 0.681 | 0.864 | 0.639 | 0.307 | 0.925 | 0.549 | 0.743 | 0.834 | 0.699 | 0.167 | 0.176 | 0.202 | 0.681 | 0.864 | 0.639 | 0.307 | 0.690 | 0.280 | 0.276 | 0.244 |
| **24** | 0.482 | 0.168 | 0.106 | 0.049 | 0.548 | 0.635 | 0.201 | 0.078 | 0.899 | 0.584 | 0.799 | 0.816 | 0.800 | 0.186 | 0.182 | 0.115 | 0.548 | 0.635 | 0.201 | 0.078 | 0.651 | 0.287 | 0.191 | 0.093 |
| **25** | 0.644 | 0.360 | 0.160 | 0.110 | 0.874 | 0.903 | 0.212 | 0.121 | 0.858 | 0.596 | 0.743 | 0.766 | 0.735 | 0.375 | 0.393 | 0.556 | 0.837 | 0.903 | 0.212 | 0.121 | 0.783 | 0.530 | 0.276 | 0.198 |
| **26** | 0.635 | 0.290 | 0.215 | 0.193 | 0.814 | 0.681 | 0.405 | 0.264 | 0.864 | 0.507 | 0.665 | 0.735 | 0.742 | 0.336 | 0.315 | 0.419 | 0.814 | 0.681 | 0.405 | 0.264 | 0.777 | 0.450 | 0.355 | 0.324 |
| **27** | 0.640 | 0.280 | 0.102 | 0.163 | 0.731 | 0.730 | 0.172 | 0.196 | 0.894 | 0.592 | 0.729 | 0.809 | 0.836 | 0.313 | 0.200 | 0.491 | 0.731 | 0.730 | 0.172 | 0.196 | 0.780 | 0.438 | 0.185 | 0.280 |
| **28** | 0.712 | 0.270 | 0.281 | 0.178 | 0.873 | 0.906 | 0.614 | 0.215 | 0.779 | 0.498 | 0.629 | 0.686 | 0.795 | 0.278 | 0.341 | 0.507 | 0.873 | 0.906 | 0.614 | 0.215 | 0.832 | 0.425 | 0.439 | 0.302 |
| **29** | 0.648 | 0.275 | 0.223 | 0.008 | 0.763 | 0.866 | 0.402 | 0.008 | 0.520 | 0.411 | 0.447 | 0.454 | 0.811 | 0.287 | 0.334 | 0.567 | 0.763 | 0.866 | 0.402 | 0.008 | 0.786 | 0.431 | 0.365 | 0.016 |
| **30** | 0.657 | 0.402 | 0.449 | 0.000 | 0.876 | 0.916 | 0.629 | 0.000 | 0.943 | 0.822 | 0.923 | 0.000 | 0.724 | 0.417 | 0.612 | 0.000 | 0.876 | 0.916 | 0.629 | 0.000 | 0.793 | 0.573 | 0.620 | 0.000 |
| **31** | 0.626 | 0.332 | 0.306 | 0.241 | 0.775 | 0.913 | 0.428 | 0.292 | 0.896 | 0.653 | 0.836 | 0.843 | 0.765 | 0.343 | 0.518 | 0.582 | 0.775 | 0.913 | 0.428 | 0.292 | 0.770 | 0.500 | 0.470 | 0.390 |
| **32** | 0.643 | 0.547 | 0.364 | 0.000 | 0.720 | 0.925 | 0.428 | 0.000 | 0.889 | 0.773 | 0.841 | 0.000 | 0.857 | 0.573 | 0.709 | 0.000 | 0.720 | 0.925 | 0.428 | 0.000 | 0.782 | 0.707 | 0.534 | 0.000 |
| **33** | 0.601 | 0.100 | 0.083 | 0.001 | 0.696 | 0.869 | 0.214 | 0.002 | 0.976 | 0.835 | 0.950 | 0.963 | 0.814 | 0.101 | 0.119 | 0.004 | 0.696 | 0.869 | 0.214 | 0.002 | 0.750 | 0.181 | 0.153 | 0.003 |
| **34** | 0.662 | 0.107 | 0.226 | 0.000 | 0.758 | 0.932 | 0.469 | 0.000 | 0.970 | 0.730 | 0.936 | 0.000 | 0.840 | 0.107 | 0.303 | 0.000 | 0.758 | 0.932 | 0.469 | 0.000 | 0.797 | 0.193 | 0.368 | 0.000 |
| **35** | 0.690 | 0.258 | 0.277 | 0.033 | 0.809 | 0.609 | 0.760 | 0.035 | 0.934 | 0.805 | 0.820 | 0.892 | 0.825 | 0.309 | 0.303 | 0.375 | 0.809 | 0.609 | 0.760 | 0.035 | 0.817 | 0.410 | 0.433 | 0.065 |
| **36** | 0.675 | 0.152 | 0.137 | 0.004 | 0.803 | 0.842 | 0.254 | 0.005 | 0.966 | 0.809 | 0.936 | 0.950 | 0.809 | 0.157 | 0.229 | 0.040 | 0.803 | 0.842 | 0.254 | 0.005 | 0.806 | 0.264 | 0.241 | 0.008 |
| **37** | 0.583 | 0.014 | 0.095 | 0.031 | 0.641 | 0.035 | 0.647 | 0.075 | 0.990 | 0.926 | 0.890 | 0.952 | 0.864 | 0.023 | 0.100 | 0.049 | 0.641 | 0.035 | 0.647 | 0.075 | 0.736 | 0.027 | 0.173 | 0.060 |
| **38** | 0.713 | 0.043 | 0.127 | 0.098 | 0.796 | 0.085 | 0.785 | 0.186 | 0.989 | 0.919 | 0.867 | 0.953 | 0.872 | 0.081 | 0.131 | 0.171 | 0.796 | 0.085 | 0.785 | 0.186 | 0.832 | 0.083 | 0.225 | 0.178 |
| **39** | 0.729 | 0.038 | 0.142 | 0.125 | 0.821 | 0.064 | 0.742 | 0.189 | 0.986 | 0.917 | 0.849 | 0.954 | 0.866 | 0.086 | 0.149 | 0.268 | 0.821 | 0.064 | 0.742 | 0.189 | 0.843 | 0.073 | 0.249 | 0.221 |
| **40** | 0.633 | 0.046 | 0.151 | 0.167 | 0.673 | 0.080 | 0.759 | 0.272 | 0.986 | 0.930 | 0.880 | 0.957 | 0.914 | 0.097 | 0.159 | 0.301 | 0.673 | 0.080 | 0.759 | 0.272 | 0.775 | 0.087 | 0.263 | 0.286 |
| **41** | 0.635 | 0.056 | 0.159 | 0.013 | 0.647 | 0.117 | 0.657 | 0.016 | 0.986 | 0.915 | 0.892 | 0.966 | 0.971 | 0.098 | 0.173 | 0.071 | 0.647 | 0.117 | 0.657 | 0.016 | 0.777 | 0.106 | 0.274 | 0.026 |
| **42** | 0.723 | 0.076 | 0.137 | 0.191 | 0.750 | 0.143 | 0.831 | 0.744 | 0.979 | 0.851 | 0.793 | 0.854 | 0.952 | 0.140 | 0.141 | 0.205 | 0.750 | 0.143 | 0.831 | 0.744 | 0.839 | 0.141 | 0.241 | 0.321 |
| **43** | 0.840 | 0.335 | 0.370 | 0.476 | 0.878 | 0.776 | 0.762 | 0.559 | 0.986 | 0.890 | 0.936 | 0.972 | 0.950 | 0.371 | 0.418 | 0.761 | 0.878 | 0.776 | 0.762 | 0.559 | 0.913 | 0.502 | 0.540 | 0.645 |
| **44** | 0.619 | 0.081 | 0.345 | 0.068 | 0.797 | 0.116 | 0.826 | 0.069 | 0.963 | 0.803 | 0.893 | 0.950 | 0.735 | 0.214 | 0.372 | 0.829 | 0.797 | 0.116 | 0.826 | 0.069 | 0.765 | 0.151 | 0.513 | 0.128 |
| **45** | 0.603 | 0.172 | 0.358 | 0.053 | 0.894 | 0.247 | 0.804 | 0.053 | 0.992 | 0.796 | 0.913 | 0.955 | 0.650 | 0.361 | 0.392 | 0.955 | 0.894 | 0.247 | 0.804 | 0.053 | 0.752 | 0.293 | 0.527 | 0.100 |
| **46** | 0.681 | 0.159 | 0.356 | 0.113 | 0.763 | 0.223 | 0.852 | 0.121 | 0.994 | 0.787 | 0.887 | 0.945 | 0.863 | 0.354 | 0.380 | 0.620 | 0.763 | 0.223 | 0.852 | 0.121 | 0.810 | 0.274 | 0.526 | 0.203 |
| **47** | 0.614 | 0.278 | 0.538 | 0.112 | 0.852 | 0.374 | 0.807 | 0.120 | 0.988 | 0.766 | 0.919 | 0.926 | 0.687 | 0.521 | 0.618 | 0.628 | 0.852 | 0.374 | 0.807 | 0.120 | 0.761 | 0.436 | 0.700 | 0.202 |
| **48** | 0.730 | 0.204 | 0.406 | 0.019 | 0.855 | 0.274 | 0.832 | 0.020 | 0.993 | 0.772 | 0.873 | 0.927 | 0.834 | 0.445 | 0.442 | 0.496 | 0.855 | 0.274 | 0.832 | 0.020 | 0.844 | 0.339 | 0.578 | 0.038 |
| **Mean** | **0.626** | **0.2629** | **0.2961** | **0.1241** | **0.730** | **0.6403** | **0.6442** | **0.1637** | **0.891** | **0.6415** | **0.7641** | **0.7642** | **0.821** | **0.3187** | **0.3706** | **0.4400** | **0.730** | **0.6403** | **0.6442** | **0.1637** | **0.7673** | **0.3975** | **0.4407** | **0.2094** |
| **Std^a^** | **0.072** | **0.1381** | **0.1345** | **0.0940** | **0.090** | **0.2860** | **0.2144** | **0.1405** | **0.088** | **0.1639** | **0.1225** | **0.2276** | **0.074** | **0.1574** | **0.1619** | **0.2667** | **0.089** | **0.2860** | **0.2144** | **0.1405** | **0.0546** | **0.1772** | **0.1602** | **0.1406** |

**a. The standard deviation value**
