# Supplementary figures and images for: Panicle-SEG: a robust image segmentation method for rice panicles in the field based on deep learning and superpixel optimization
Source: Plant Methods. 2017 Nov 28;13:104. doi: 10.1186/s13007-017-0254-7 (PMC5704426; doi:10.1186/s13007-017-0254-7)

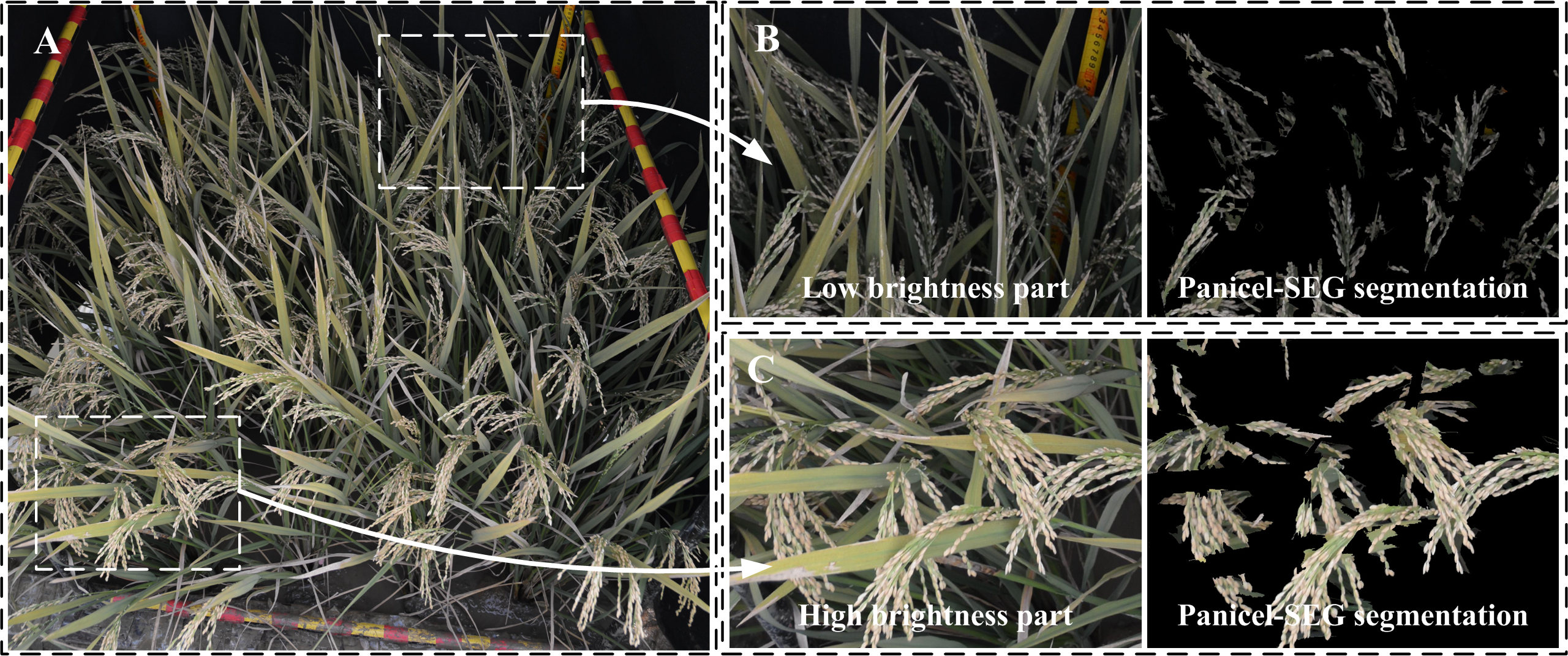

Supplement: Supplementary file 5 — Additional file 5: Figure S1. Different regions in the field plot image have different illumination condition. [file 13007_2017_254_MOESM5_ESM.tif]

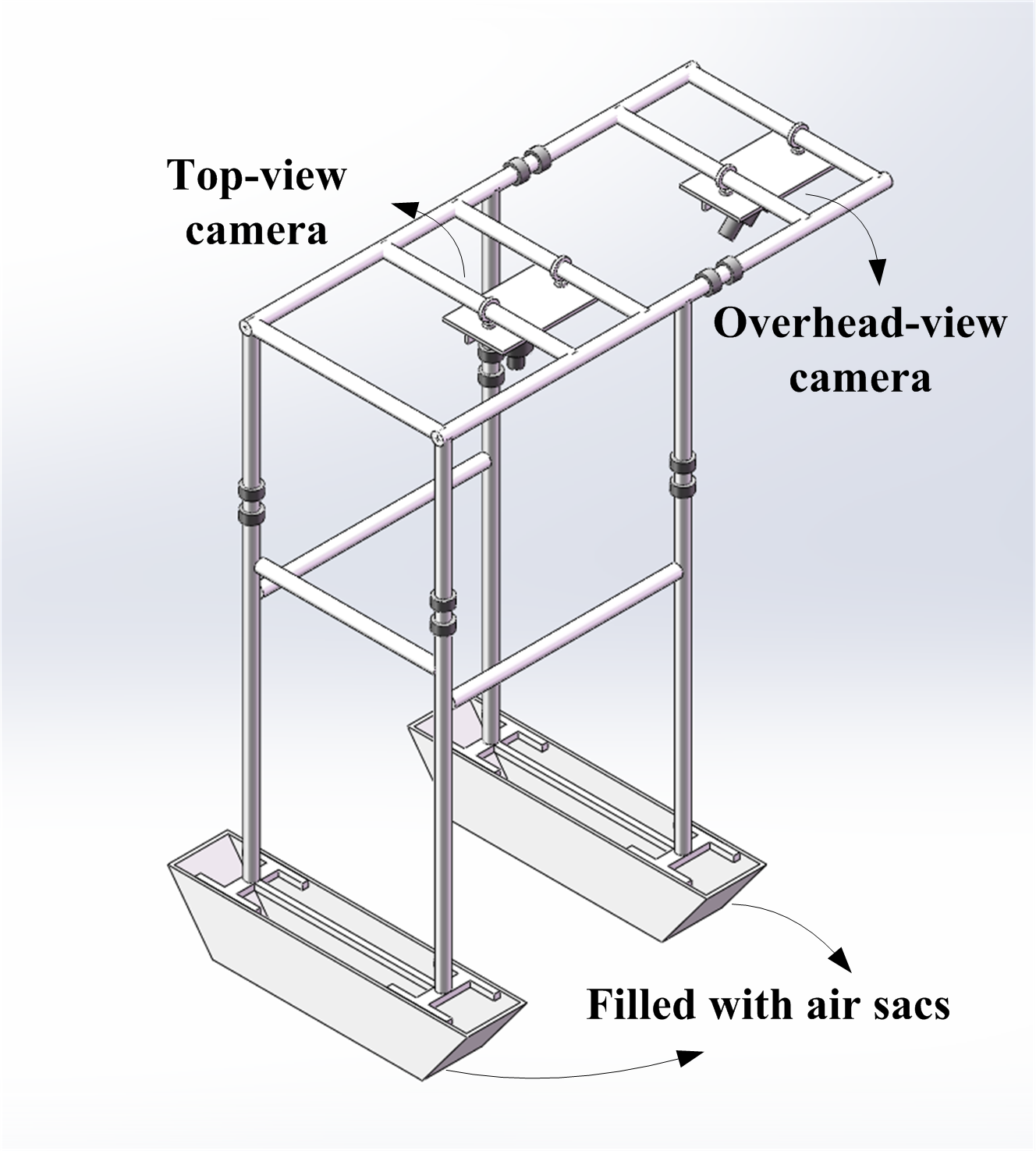

Supplement: Supplementary file 6 — Additional file 6: Figure S2. Imaging bracket with two cameras (top-view and overhead-view). [file 13007_2017_254_MOESM6_ESM.tif]

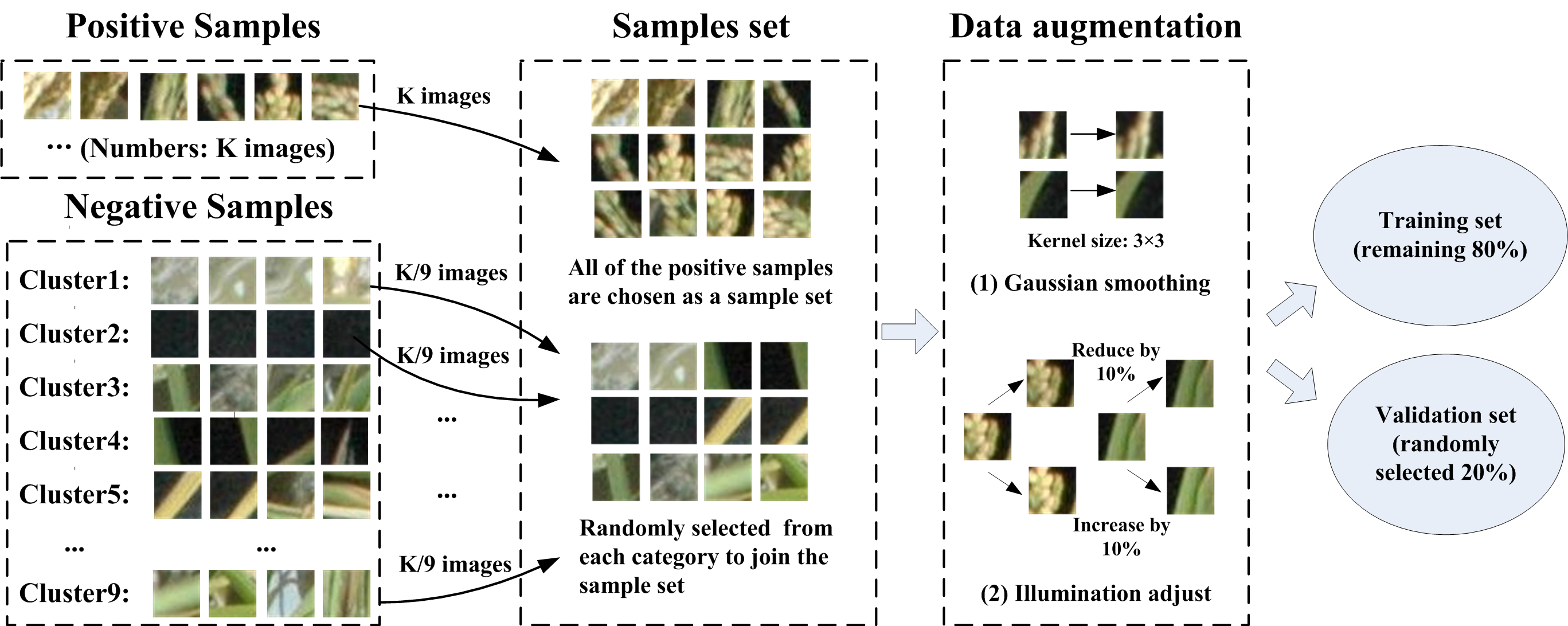

Supplement: Supplementary file 8 — Additional file 8. Figure S3. Data augmentation and sample set building. [file 13007_2017_254_MOESM8_ESM.tif]

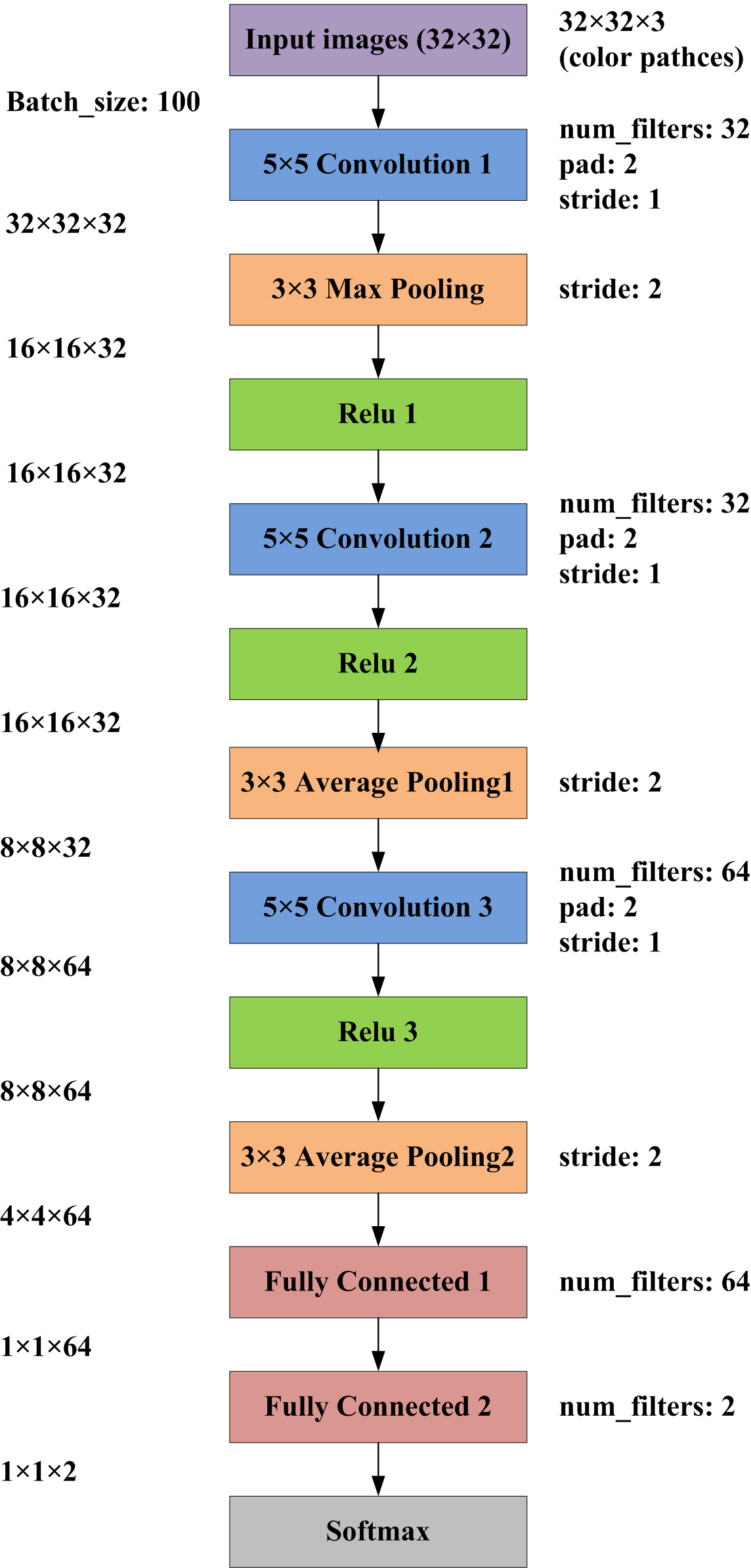

Supplement: Supplementary file 9 — Additional file 9: Figure S4. The CNN architectures applied in Panicle-SEG algorithm. [file 13007_2017_254_MOESM9_ESM.tif]
