# Supplementary material for: Panicle-SEG: a robust image segmentation method for rice panicles in the field based on deep learning and superpixel optimization
Source: Plant Methods. 2017 Nov 28;13:104. doi: 10.1186/s13007-017-0254-7 (PMC5704426; doi:10.1186/s13007-017-0254-7)
Supplement: Supplementary file 7 — Additional file 7: Appendix S2. Panicle-SEG technical documentation. [file 13007_2017_254_MOESM7_ESM.docx]

**Panicle-SEG software technical documentation**

**Plant Growth and Imaging** **bracket**

In this study, the experimental paddy field with a total area of 1200 m^2^ was located in Wuhan, Hubei province, China (30.5N, 114.3E). Rice (*O. sativa*) seeds were sown and germinated during the summer of 2016. The field plot farming method was explained as followed. Each field plot (90×90 cm^2^) had 20 rice plants of the same accession, which were planted in five rows and four columns. Considered the edge effect, a guard row of rice plants was planted on the boundary between two adjacent plots. In total, 71 rice accessions were used for training and testing processing in this work. For each plot, two images (top-view and overhead-view, respectively) were extracted. The imaging bracket (shown in Figure 1) was used to obtain rice plot images. Two cameras (Nikon D40 camera hosting a 23.7×15.6mm CCD matrix, 35mm focal length lens, 3008×2000 pixels and Nikon D7100 camera hosting a 23.5×15.6mm CMOS matrix, 17mm focal length lens, 4000×6000 pixels), including top-view and overhead-view camera were mounted at the top and the side of the imaging bracket, respectively. Wireless shutter was used to trigger the cameras to take images when the imaging bracket moving manually in the paddy field. The bottom of the imaging bracket was designed into a hollow structure, which was filled with air sacs to facilitate moving in paddy field.


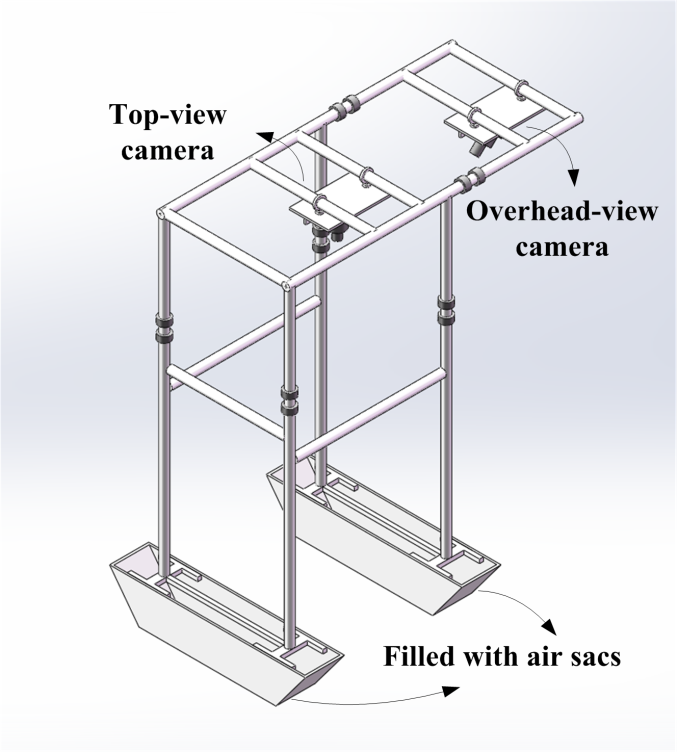


**Figure 1 Imaging bracket with two cameras (top-view and overhead-view).**

**Description the growth state of rice in the field environment**

The environments, including illumination, weather, and water reflection, vary greatly and make image analysis of rice panicle difficult. Not only that, the difference between rice varieties can also lead to panicle in shape, size, posture and color differences. Also, the reproductive stage will influence the color of rice panicle. From an image perspective, water reflection (Figure 2A), illumination unbalance (the illumination unbalance in the same plot, Figure 2B), cluttered background (yellowish rice leaves and serious overlapping, Figure 2C) are all pose challenges for panicle segmentation. Especially, the different rice varieties will cause the variance in panicle shape, size, posture and color (Figure 3). Furthermore, different weather condition is another challenge, which will cause the differences in illumination and panicle color (Figure 4). Also, the reproductive stage will influence the color of rice panicle (Figure 5). So, in this paper, all these conditions (the illumination, weather condition, rice accession, cluster background, reproductive stage) should be considered when building the training set. Moreover, in the training process, not only the top-view in the field is considered, but also the overhead-view in field environment (like Figure 5) and indoor pot-grown top-view/side-view images.


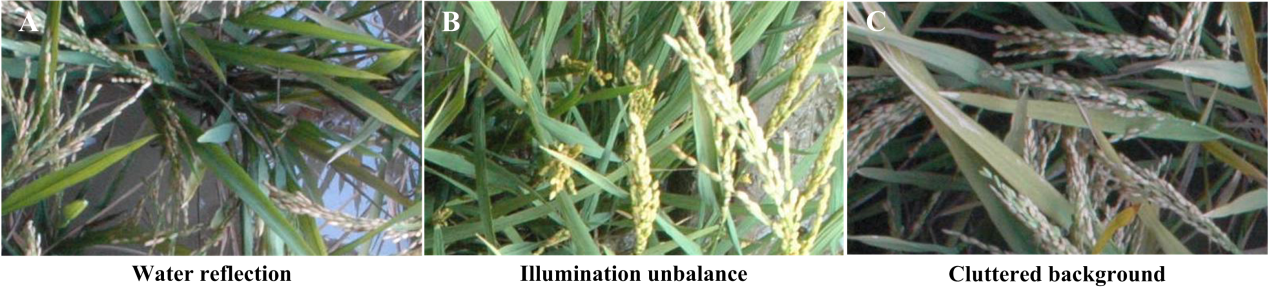
**Figure 2 Challenges in the field based rice panicle segmentation.** (A) Water reflection, (B) The illumination unbalance in the same plot, (C) Yellowish rice leaves and serious overlapping

**
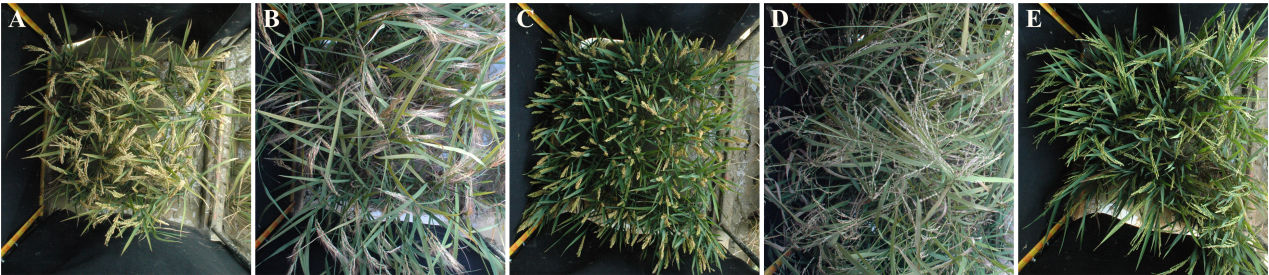
**

**Figure 3 Variance in shape, size and posture among panicles from different rice varieties.**

**
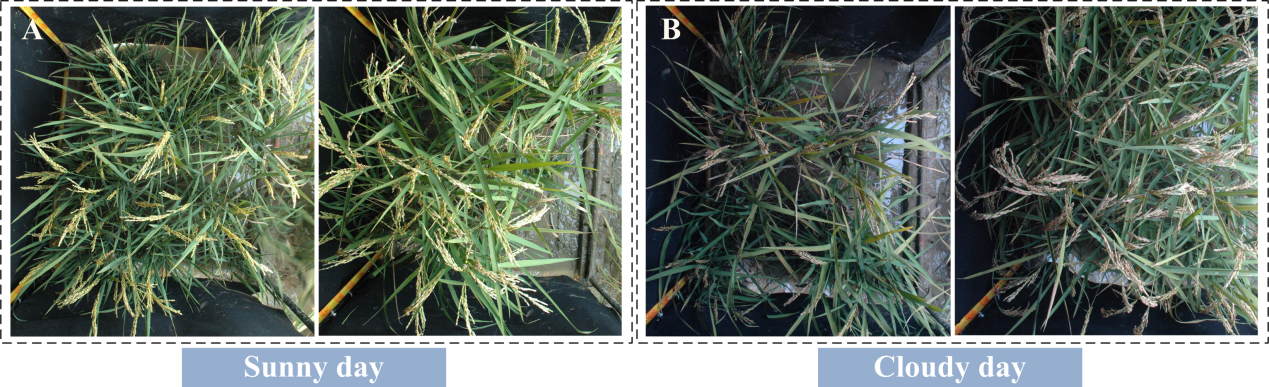
**

**Figure 4 Differences in illumination and panicle color caused by different weather conditions.** (A) Sunny day. (B) The weather is cloudy day.


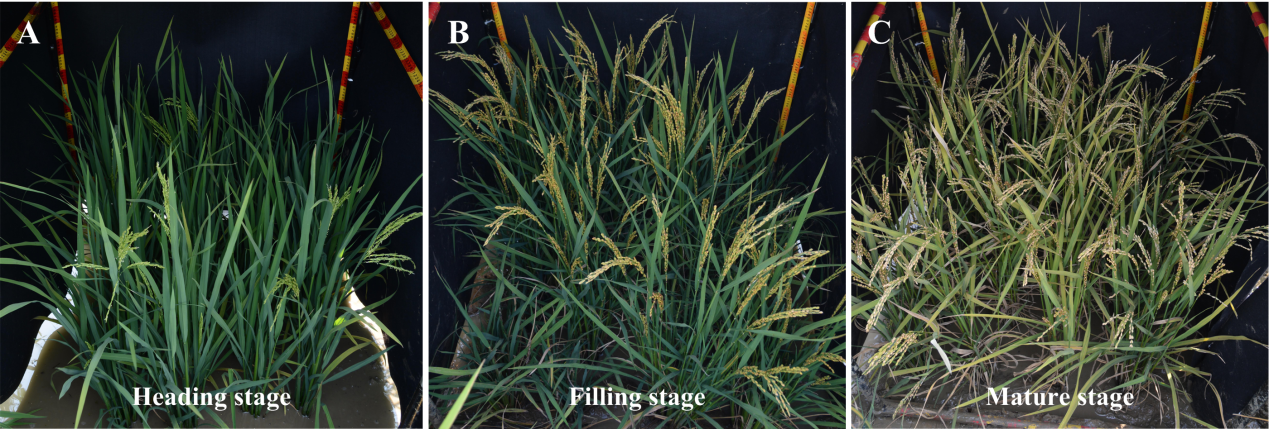


**Figure 5 Differences in reproductive stage from overhead-view.** (A) Heading stage, (B) Filling stage, (C) Mature stage

**Solution Selection and technology roadmap**

Our problem is actually to solve the problem of object (rice panicle) segmentation in complex background (field environment) and we expect our algorithm to be as robust as possible to suit most situations (different camera angle; different panicle size, panicle shape, and color; different weather, illumination, reproductive stage and so on). The classical segmentation method (FCN [1] network in deep learning) cannot be very good to ensure the edge-preserving of rice panicle. So, we start to consider other solutions. For reference to the thought of RCNN [2], we first build several candidate regions, which were generated using superpixel method. The superpixel method can exhibit state-of-the-art boundary adherence. Then use CNN to classify panicle or background. The detailed technology roadmap is shown below (Figure 6):


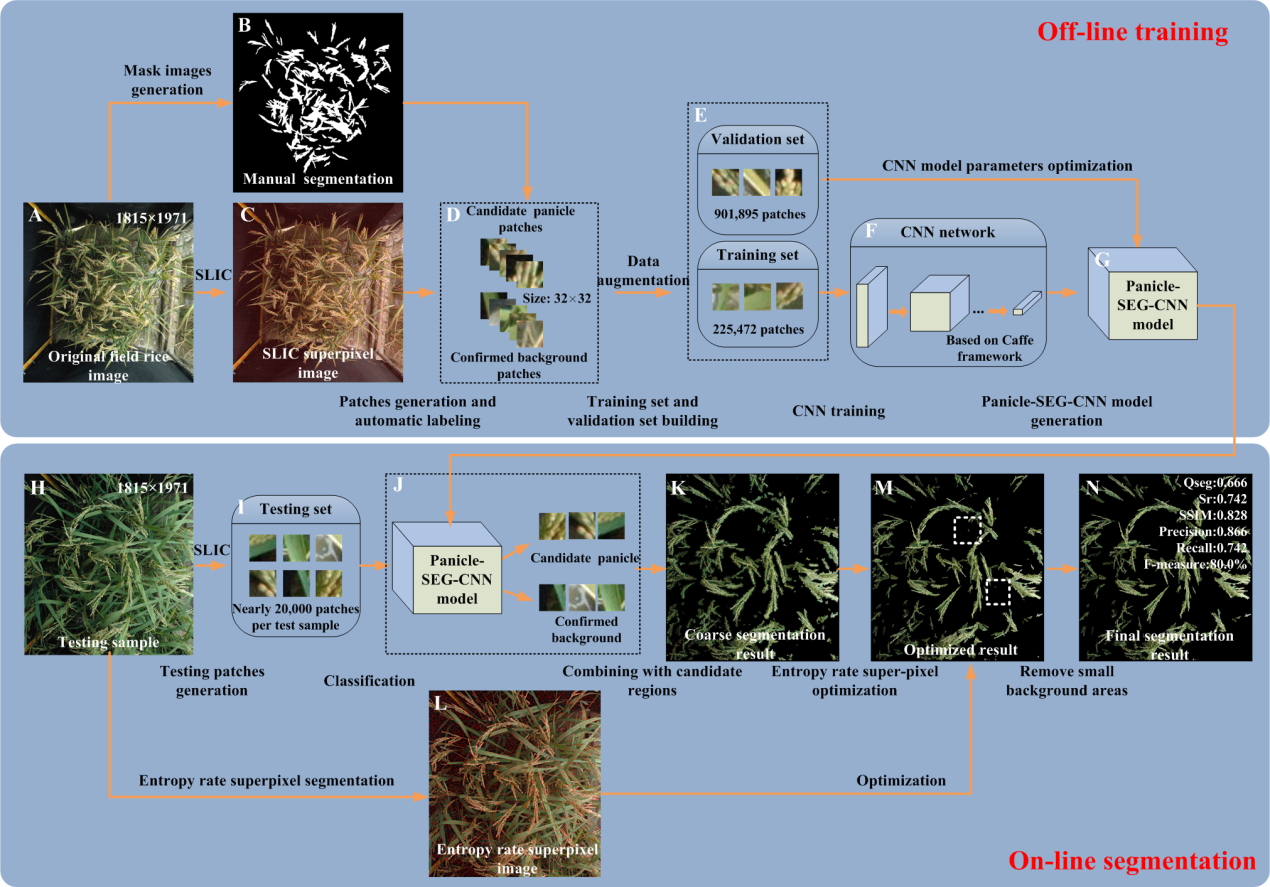


**Figure 6 The technical roadmap for Panicle-SEG algorithm**

After the image acquisition, the main flow diagram of our rice panicle segmentation algorithm (Panicle-SEG algorithm) including off-line training and on-line segmentation is shown in Figure 6. To make the algorithm robust, we have expanded our training samples, including 49 top-view field rice images, 30 overhead-head view field rice images, 302 pot-grown rice side-view images and 303 pot-grown rice top-view images. Here, the top-view field plot rice image (Figure 6A) with a resolution of 1815×1971 pixels was selected as an example to illustrate the technical route. The technical route mainly consists of two processes: off-line training and on-line segmentation. The off-line training contained 4 steps: (1) Generation of patches: images were manually segmented using Photoshop software to obtain the mask images (Figure 6B) for the following automatic labeling. Patches were generated using SLIC superpixel segmentation (Figure 6C); (2) Automatic labeling: the patches were automatically labeled into 2 categories: candidate panicle and confirmed background (Figure 6D); (3) Training set and validation set building: The patches were augmented and divided into the training set (901,895 patches) and validation set (225,472 patches) (Figure 6E); (4) CNN training and Panicle-SEG-CNN model generation (Figure 6F-G). Then, for a testing image (Figure 6H), the on-line segmentation included 3 steps: (1) Generation of patches: The testing patches were generated using SLIC superpixel segmentation (Figure 6I); (2) Coarse segmentation by using a pre-trained Panicle-SEG-CNN model: The pre-trained Panicle-SEG-CNN model generated in off-line training is utilized in testing patch classification, and the testing patches were categorized into candidate panicles and confirmed background (Figure 6J). Then the candidate panicle patches were merged into one image, called the coarse segmentation result (Figure 6K); (3) Entropy rate superpixel optimization: The coarse segmentation result was combined with the entropy rate superpixel image (Figure 6L) to obtain the optimized segmentation result (Figure 6M). The final segmentation result (Figure 6N) was obtained after removing small background region.

**Detailed methodological information**

**Part1: Off-line training**

**(1) Manual segmentation using PhotoShop**

After the original images were acquired, the ROI region was extracted to remove background (Figure 7A) in the training process. Then, images were manually segmented using Photoshop software to obtain the mask images. This task is required to ensure the edge integrity of the foreground panicle region. So, operators should be as careful as possible. The resolution of the cropped image (ROI region) is 1815×1971 pixels (Figure 7B). And the foreground gray value is set to 255 and background gray value is set to 0 (Figure 7C). Generally, manually segmenting an image with resolution of 1815×1971 pixels takes approximately 4-5 hours.

**
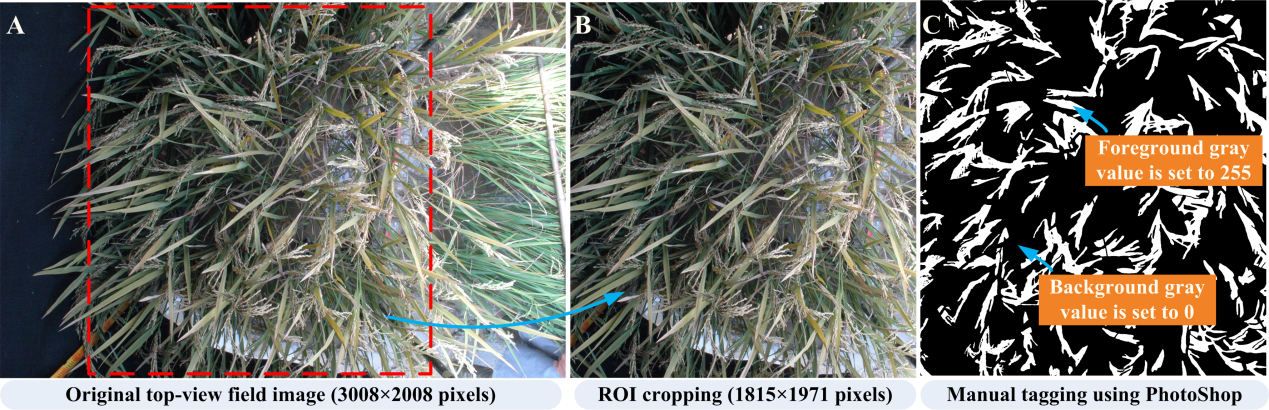
**

**Figure 7 Manual segmentation using PhotoShop and ROI region extraction**

**(2)** **Simple Linear Iterative Clustering (SLIC)** [3]

SLIC is an adaptation of k-means for superpixel generation, with two important distinctions: The distance measurement in the SLIC clustering algorithm considers both the color similarity and the Euclidean distance of pixel coordinates. So the central point of a cluster is composed of five-D vectors. This includes recording the pixels under the LAB color space and the XY coordinate of the pixel point. SLIC algorithm has two parameters: K and M. K is the number of superpixel, and M is superpixel compact degree. In our paper, the compact coefficient (M) is set to 10 and remains unchanged for all the samples (training set and testing set). In the training process, the number of superpixel (K) is set to 20,000 for the training images with a resolution of 1815×1971 pixels. For different size of input images in the testing process, we expect the area of each superpixel regions to be basically consistent. So, the fixed proportional relation (1815×1971/20000) was used to calculate the number of superpixel (K) for a new testing sample. The calculation formula is expressed in C + + language as:

where, image.rows and image.cols was the number of rows and columns in the testing sample.

The main steps for Simple Linear Iterative Clustering:

**Step 1:** Initialization. By sampling the pixel of the original image, the centers of the K Clusters (denoted as **C_K)** are initialized. Enter a parameter K for the number of superpixel. Then for an N-pixel image, each superpixel size is about N / K pixels. The distance between each two contiguous superpixel regions is:

$S\text{=}\sqrt{N/K}$ (1)

So, the initial cluster size is **S**, and the image is divided into **K** meshes. Each grid center is the initial cluster center.

Then, initialize each pixel point's label value to be **-1**, and the distance between each pixel point and the center of the cluster to be infinity.

**Step 2:** Moving the initialization of the cluster centers. Firstly, the minimum gradient direction was found in the 3×3 neighborhood centered on each cluster centers. Then, the cluster center was update to the point corresponding to the minimum gradient direction.

**Step 3:** Assignment step. The area of each superpixel is approximately the square of S squared. We can safely assume that pixels are within the 2S×2S range of the cluster center. This range is the search range for each cluster center. So, for each cluster center **C_K**, consider all pixel points in the **2S ×2S** neighborhood of the **center point**, and calculate the distance of each point to **C_K**.

 (2)

 (3)

$D\text{=}\sqrt{d_{c}^{2}+(\frac{d_{s}}{S})^{2}M^{2}}$ (4)

(Where, M is the superpixel compact degree)

If$D<distance(i)$, then assigns$distance(i)=D$, and assigns the label of the point as **K**.

**Step 4:** Update Cluster Center. To find all the points with label values of **K**, and obtain the average value as the new cluster center.

**Step 5:** Calculate residual error. Calculate the error by iterating Step 3 and Step 4 until $Error<threshold$

In practical applications, the parameter (K) settings in our rice panicle segmentation algorithm have a great influence on the final effect. Here, the number of SLIC superpixel regions is evaluated. Figure 8 show the discussion for the region number of SLIC superpixel segmentation. (A), (B), (C), (D) represents the number of SLIC superpixel regions is 1000, 5000, 10000 and 20000, respectively. As the number of SLIC superpixel region increases, the boundary adherence is enhanced. Compared with (A), (B) and (C), each SLIC superpixel region in (D) nearly contains pixels of the same category (panicle pixels or background). While, the bigger the number of SLIC superpixel regions, the smaller the area of those regions. Thus, to avoid the over segmentation and speed up the segmentation, the number of SLIC superpixel regions yet should not be too much. In practice, 20000 is a good choice for the testing field rice image with resolution of 1815×1971 pixels. Of course, the number of superpixel should be correspondingly adjusted with different input image resolution as above discussed.


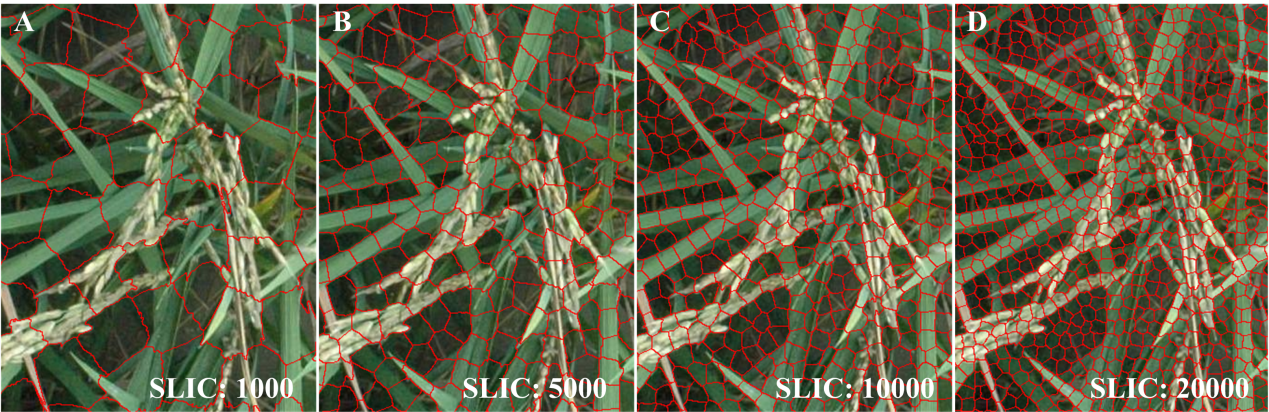


**Figure 8 The discussion for region number of SLIC superpixel segmentation algorithm**

**(3) Generation of patches**

In this study, the size of 32×32 pixels was applied to generate sample patches. There are three reasons for choosing 32×32 here. One reason is that although CNN can be applied to images of any reasonable size, but once trained at a certain size, this must remain consistent. So, the fixed size of sample patches should be used. Another reason is that in order to make the boundary of the SLIC cluster well fit to the edge of the panicle, the number of superpixel is always big. Accordingly, the area of SLIC superpixel region is relative small (approximately 14×14 pixels). 32×32 patch can contain the entire SLIC superpixel region and introduce appropriate surrounding information. Furthermore, the sample image's size of Cifar10 (a standard training dataset) is also 32×32 pixels. So, the architecture of Cifar10 can be adopted. In this study, the shape of these SLIC superpixel regions is irregular, and thus, they cannot be directly used as CNN input. Therefore, a small window called a patch, which is centered on the weighted center of the SLIC superpixel region, is given to the CNN as input. Actually, we did four different attempts before we made this choice. 1. The size of patch is 14×14 pixels, 2. The size of patch is 14×14 pixels, then, resize to 32×32 pixels (use bilinear interpolation method to resize), 3. Cropping 32×32 patch (it is centered on the weighted center of the SLIC superpixel region), with the gray value of surrounding pixels setting as 0 or 255, 4. The size of patch is larger than 32×32 pixels.

Attempt 1 and Attempt 2 only considered SLIC superpixel region, ignoring the surrounding region information. Results showed that 14×14 pixels patch was too small to provide sufficient and effective information for the following classification. The size of attempt 3 was 32×32 pixels, but setting gray value of the surrounding pixels as 0 or 255 still cannot provide sufficient information for classification. Attempt 4 was a viable solution. But, the size of patch should not be too large, or it would introduce too much background information. In practice, the size of 32×32 pixels was used after analysis.

In the concrete operation, the edge of the images should be specially considered to avoid the occurrence of cross-border phenomena in the generation of sample patches. The expression in C + + language is as follows:

All the sample patches are saved to the local disk and renamed for retrieval. The naming rule is: the corresponding original image name plus SLIC superpixel region label number. For example, the original image name is "4.jpg" and the SLIC superpixel region label number is 3158. Then, the new patch name is "4_3158.jpg". Here, the SLIC superpixel region label number can be obtained from the above SLIC operation and it will be used for the following steps.


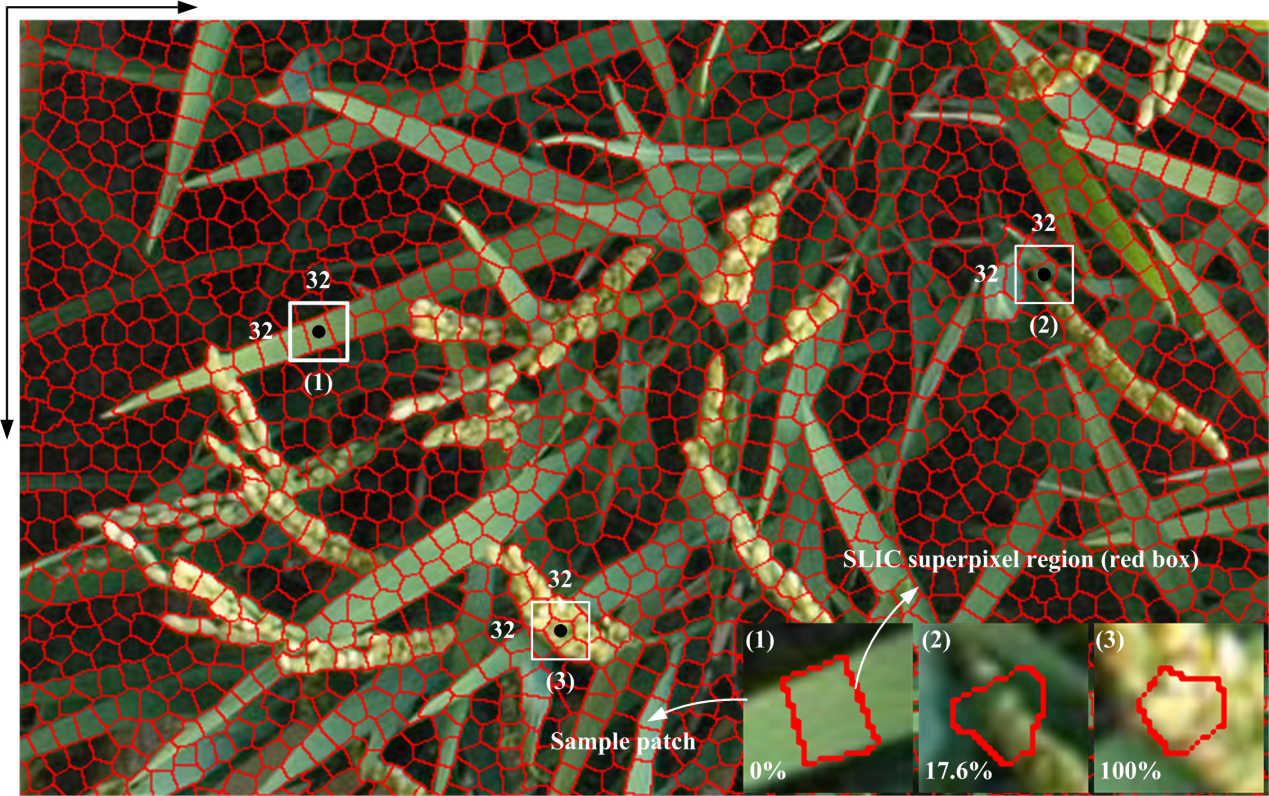


**Figure 9 Patch generation approaches based on SLIC.**

So, how does 32×32 patches get extracted? Firstly, SLIC superpixel regions were generated as discussed in the previous section, such as the red irregular polygons in Figure 9. The black point represents the weighted center of the SLIC superpixel region. The region centered on the black point and enclosed by a white box (32×32 pixels) is the sample patch on the corresponding SLIC superpixel region. The boxes (1), (2), and (3) in Figure 9 are the zoom-in versions of the white box training patches.

**(4) Automatic labeling**

CNN offline training is a supervised process, which means that the input patches and their corresponding labels are both needed. As the numbers of samples is enormous (training samples: 901,895 and validation samples: 225,472 in this paper), it is unrealistic to label each patch manually. Thus, a rapid and accurate labeling method was proposed in this work. Two images are needed. The first image is an original field rice image, and the second image is the corresponding mask image with a high degree of segmentation accuracy, which is obtained manually using PhotoShop software. The white part in the mask image represents the foreground area of the rice panicle (see in section: Manual segmentation using PhotoShop). Firstly, the sample patches were generated as discussed in the previous section. The percentages in each patch represent the ratio between the foreground panicle areas (the white part in the mask image) to the corresponding areas of the SLIC superpixel region (the percentages show in boxes (1), (2), and (3) in Figure 9). The sample patch is labeled as category 0 (confirmed background) if the percentage of the current patch is equal to zero; otherwise, it is labeled as category 1 (candidate panicle region). That is, the SLIC superpixel region in the sample patch that confirms the background has no foreground pixels at all. As long as the foreground pixel exists, the current sample patch is labeled as a candidate region for panicle. In fact, we've tried several ways to label sample patches before using this one. For example, we use the percentage as the basis for labeling distinction. Such as, we think of it as a four classification problem. The first label is the percentage accounted for over 90%. The second label is the percentage less than 90%, and greater than 20%. The percentage of the third label is less than 20%, and greater than 0%. The fourth label is equal to 0%. Of course, the percentage parameter of these four labels can be transformed. For the above situation, the expression in C + + language as:

The idea is to draw lessons from the label idea in Grabcut [4] segmentation. The four labels are confirmed foreground, candidate foreground, candidate background, and confirmed background, respectively. Compared with the two class classification, four class classifications is more complex. More importantly, if classified in this way, we need to do different things for all the four situations. Since later processing is also required, the categorization of labels is not necessarily so strict. So, two classifications (confirmed background candidate for panicle) method is proposed. The advantage of this labeling method is that it guarantees that all of the categories 0 samples do not contain panicle pixels, which can improve the classification accuracy of the CNN. However, the labeling method will cause a problem. As long as the patch has foreground panicle pixels, regardless of how many it has, the labeling method will still tag it to a candidate panicle patch, which means that the coarse segmentation result is likely to contain background pixels. Thus, the optimization processing based entropy rate superpixel is needed, which will be explained in detail later.

**(4) Training set and validation set building and data augmentation**

Given the patch labeling proposals, the next question is how to build a training set and validation set. There are some questions needed to consider when building sample set. The first one is the imbalance of positive and negative samples. For the progress of the sample patches generation, the number of category 0 (negative) samples is far larger than the number of category 1 (positive) samples. Thus, samples balance is necessary. In this study, all of the positive samples are chosen. For the negative samples, the Gaussian Mixture Model (GMM) [5] is applied for unsupervised clustering. The mean value and standard deviation of each training patches in RGB color space are extracted as the input vector of GMM (the expression in C + + language as below). In total, 9 categories are obtained, and equivalent samples are selected randomly from each category to join the sample set to keep the samples balance (Figure 10).

Considering the complication of the field-based environment, the next step is to augment the dataset. Thus, to simulate the illumination change, the intensity component of the sample patches in HSV color space is adjusted (default adjustment plus or minus 10%). The corresponding adjustment function in C + + language as:

At the same time, the wind influence and image defocus phenomenon is also common in the field-based imaging environment, which will cause image blur (The focus of the camera is not gathered by all the panicles. So, in this way, the image details are vague if your look at image details by enlarging local region). To simulate this phenomenon, Gaussian blur is adopted to simulate this situation. The size of smoothing Gaussian kernel is 3×3. The corresponding adjustment function in C + + language as:

These augmented images are all added to the sample set (Figure 10), from which twenty percent of the sample set is randomly selected as the validation set (225,472 patches), and the remaining eighty percent is selected as the training set (901,895 patches).


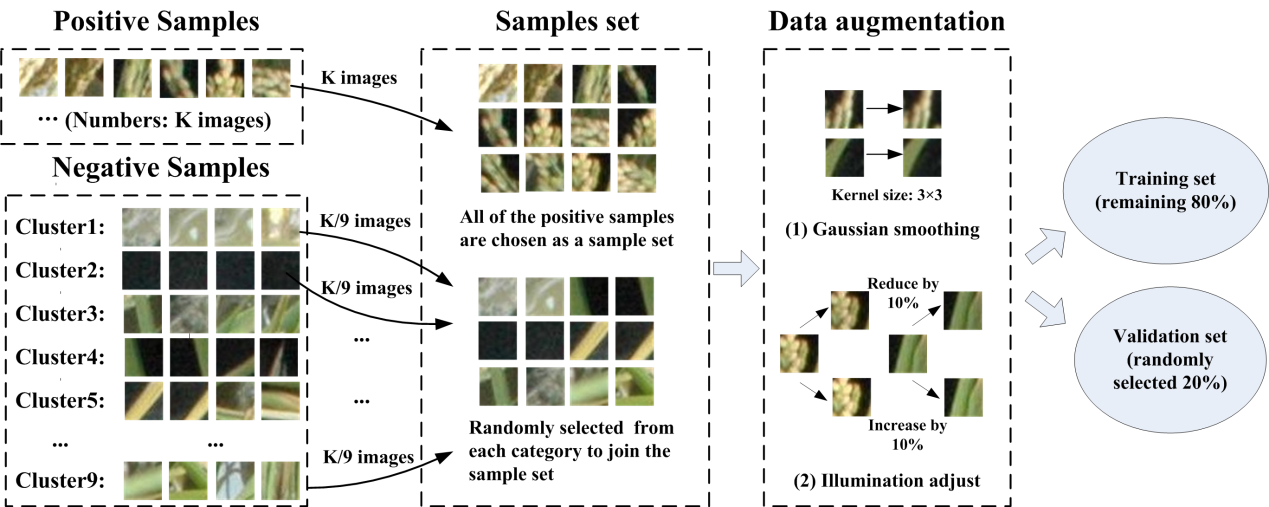


**Figure 10 Data augmentation and sample set building**

**(5) CNN training and Panicle-SEG-CNN model generation**

1. Why we choose CNN as a classifier?

For classification problems, the traditional machine learning algorithms, such as Support Vector Machine (SVM) and Artificial Neural Network (ANN), needs to acquire hand-crafted features. But, these hand-crafted features are not guaranteed to provide the subsequent learning algorithm with the optimal description of the data. Meanwhile, it is hard to extract satisfactory features for all complicated situations. Convolutional Neural Network (CNN) is very similar to ANN: they are made up of neurons that have learnable weights and biases. Except for the difference that the inputs of CNN are images, and that means the CNN has ability to learn features independently instead of extracting features manually. Furthermore, thanks to the structural optimization of CNN in recent years, it has been widely used in object recognition task, especially for the classification of complex environments (illumination changes, morphological differences, etc).

2. What is CNN?

A CNN is composed of several stacked layers of “neurons”. The neurons are small computing elements which take an input, pass it through a function and yield an output. Neurons are connected to other neurons in upper or lower layers with each neuron link having a certain weight. Information flows through these connections. The input on the first layer of neurons is the image to be processed. Output of the first layer neurons is simply the values of the features computed on the image. These values then go through the network, undergoing subsampling, non linear transformation and linear combination as they pass through the layers, to finally yield an output: the label of the image. We use four main types of layers to build CNN architectures in this paper: Convolutional Layer, Pooling Layer, Non-Linear Layer and Fully-Connected Layer (exactly as seen in regular Artificial Neural Networks). We will stack these layers to form a full CNN architecture (the architecture we used was shown in Figure 11). The Convolutional layer (CONV) is the core building block of a Convolutional Network that does most of the computational heavy lifting. CONV layer will compute the output of neurons that are connected to local regions in the input, each computing a dot product between their weights and a small region they are connected to in the input volume. Three hyper parameters control the size of the output volume: the depth, stride and zero-padding. Depth is related to the number of input filters. This may result in volume such as [32x32x32] if we decided to use 32 filters for the input image (32x32x3). The stride is set to 2 (or uncommonly 3 or more), which means that we jump 2 pixels at a time as we slide the filter around. This will produce smaller output volumes spatially. Sometimes, it will be convenient to pad the input volume with zeros around the border to allow us to control the spatial size of the output volumes (zero-padding). And it is common to periodically insert a Pooling layer in-between successive CONVs in a CNN architecture. The role of POOL layer is to perform a down sampling operation along the spatial dimensions (width, height), which will reduce the amount of parameters and computation in the network. Neural networks require non-linear functions between layers in order to capture the complex non-linearity of the classification tasks. In this work, the non-rectified linear unit (Relu) was applied between all CONV layers. RELU layer will apply an elementwise activation function, such as the MAX and AVERAGE. Fully-connected layer will compute the class scores, resulting in volume of size [1x1x2], where each of the 2 numbers corresponds to a class score (in this work: candidate for panicle and confirmed background). As with ordinary Neural Networks and as the name implies, each neuron in this layer will be connected to all the numbers in the previous layer.


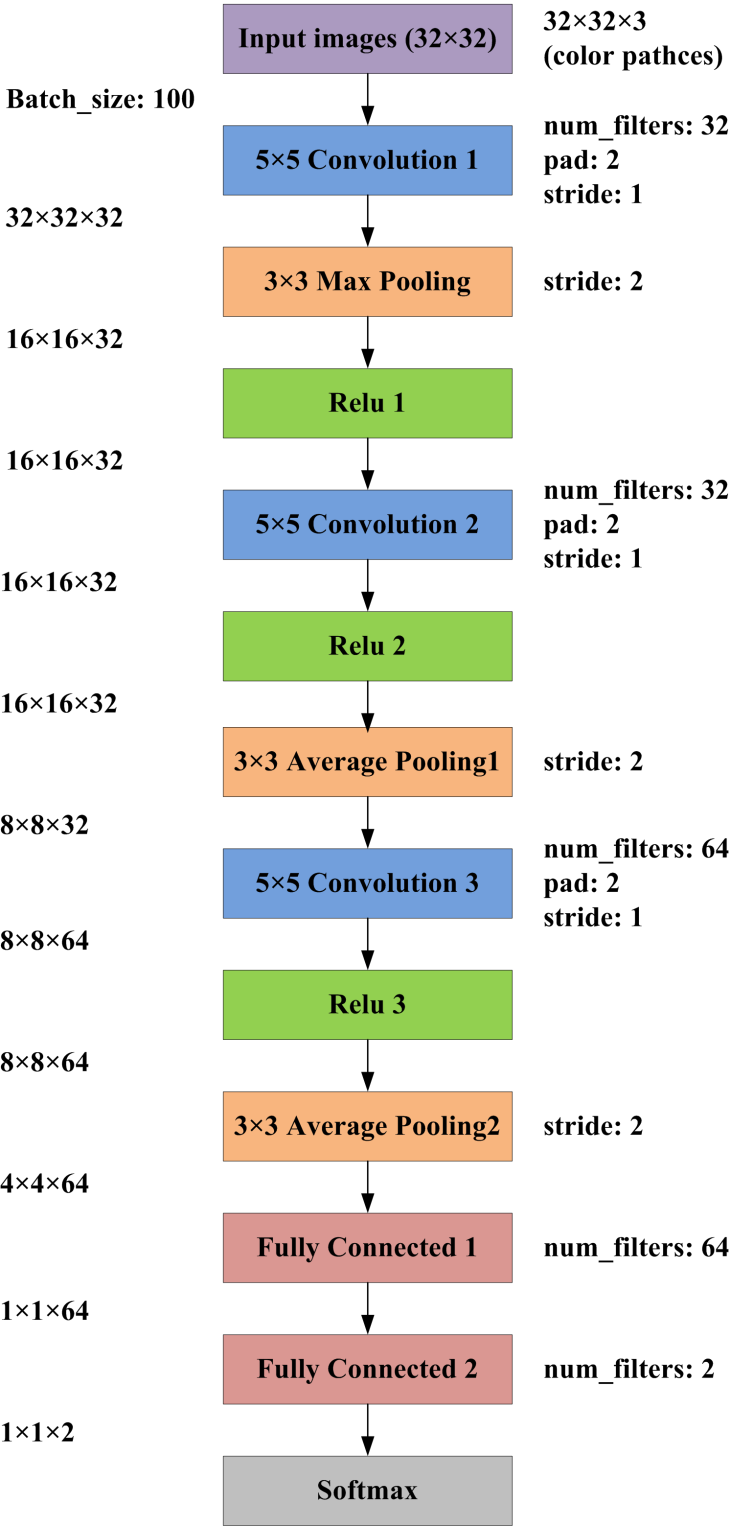


**Figure 11 The CNN architectures applied in Panicle-SEG algorithm**

The CNN workflow is shown as follows: During the training phase, features are computed for one image on the first layer, and then flowed through the layers. A label is predicted: some neurons will have probably weighted in for one class, and some for the other. The predicted label is compared to the actual label. If the prediction is correct, then all neurons in the pathways which lead to this prediction are enhanced, i.e. they get more weight in the decision process next time, and all neurons in the wrong pathways are reduced. As training progresses, the best neurons are "selected" for the decision process.

3. How to design CNN network conveniently? (Caffe framework)

As a simple metaphor, a set of deep learning frameworks is a building block, and each component is part of a model or algorithm, and you can design how to use blocks to stack blocks that fit your dataset. The appearance of the deep learning framework lowers the threshold of entry, and you don't need to start coding from a complex CNN network. Instead, you can use existing models, model parameters for your own training, and you can add your own layer to the existing model, or choose the classifier you want. The Caffe, as an open source deep learning framework was applied in this work [6]. The core of framework is developed in C++ and provides richly interfaces, like Python, Matlab and C++ command line mode. In Caffe, all the networks information and optimization profiles are described using Google Protocol Buffers. So, using the C++ command line interface, you can train a model almost without coding. The specific steps are as follows:

**Step1:** Caffe environment construction (windows10+vs2013+cuda7.5+cuDNN v4+caffe for my computer)

In a windows environment, some tools are used: Git, Anaconda2 and Nodepad++.

**Step2:** Convert original image data into Caffe available data format (LMDB/LEVELDB, we use LEVELDB format, Figure 12). In fact, Caffe also supports many other forms of input, such as ImageData, Hdf5data, but after experiments, these data consumption of a large number of IO operations will greatly increase training time. So, we still choose the LMDB/LEVELDB format.

Scripting create_leveldb.sh, then use Git to run the transformation (The coding as below). The name of the "sh" suffix is the Linux script file, which, under Windows system, should download Git in order to execute the file. Then click the right button for sh file, select Git-bash mode for running.

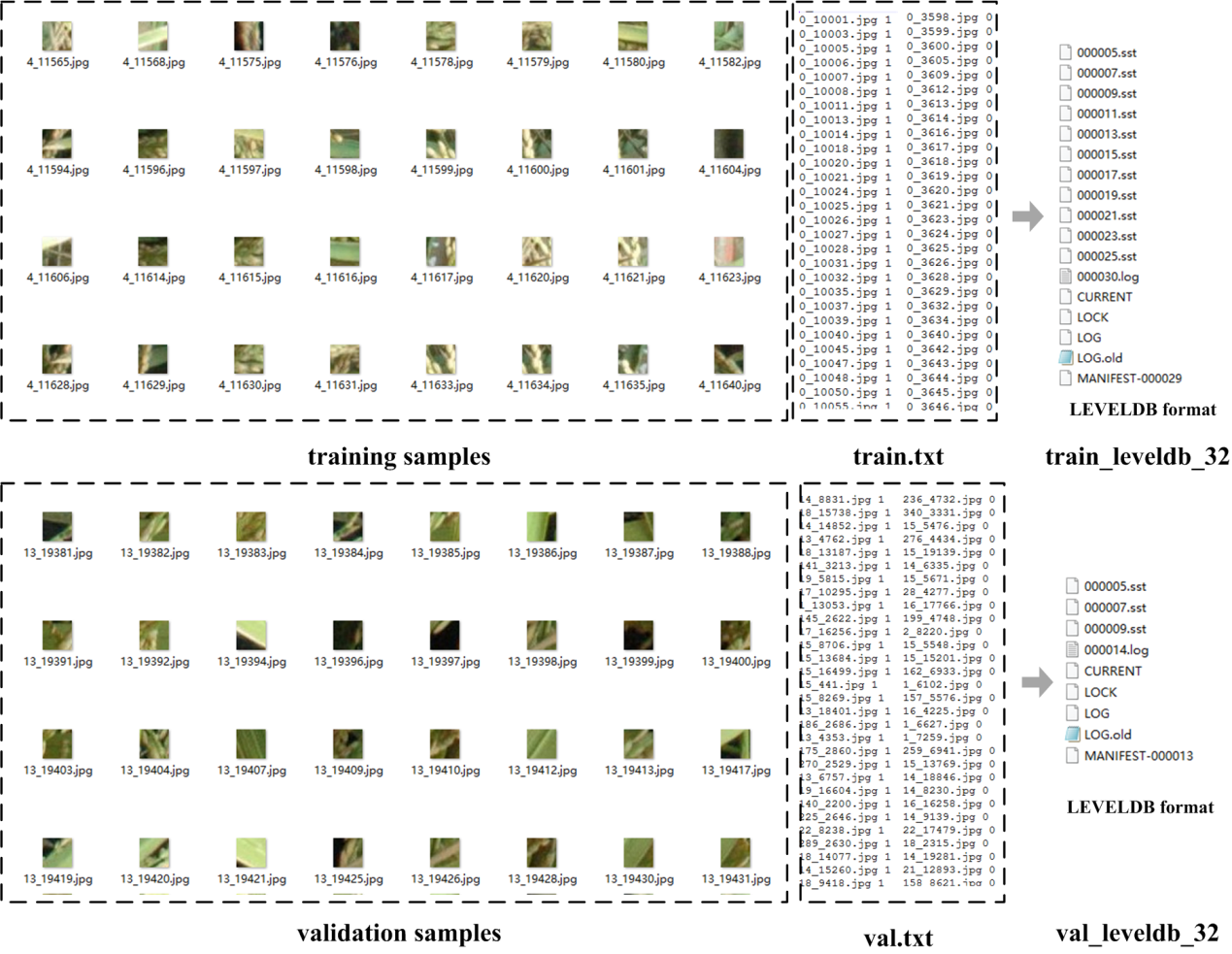


**Figure 12 Converting training sets and validation set images into LEVELDB format.**

**Step3:** Calculate the mean value of the training samples, and save the result file into a binary file format (mean_leveldb_32.binaryproto). Script compute_image_mean.sh, and then use Git to run (The codes as listed below).

**Step4:** In this step, we're going to start building our own network and write optimization files. In Caffe, all the networks information and optimization profiles are described using Google Protocol Buffers (end with "prototxt" suffix). So, we just need to write the Prototxt file without having to write the code ourselves. Here, "train_test_network.prototxt" is the network model and "solver.prototxt" is the optimization profiles.

(Data layer in train_test_network.prototxt)

Each layer is the contents of the curly brace behind the "layer". "type" represents the types of each layer, where "data" represents the data layer, with only two input data formats, namely LMDB and LEVELDB. The final data format is determined by "backend" in the "data param" section. "top" means the input. Since this is a supervised training process, the data and labels need to be known during the training process. "transform_param" section is used for the data pre-processing. The common data preprocessing operation is to subtract the mean value of the training samples, that is, each image (including training set, verification set and test set) must be subtracted by the mean image from each pixel before it is classified. So, "mean_file" is the address of the binary file for the mean image. Finally, the "data_param" section represents the data information. "source" is the address of input training data in LEVELDB format. "batch_size" is equal to 100 which mean that each time 100 training images were put into GPU memory to prevent the overflow of graphics memory due to the excessive number of training samples, which will improve training efficiency.

(CONV layer, POOL layer and RELU layer in train_test_network.prototxt)

(solver.prototxt)

The optimization profile (solver.prototxt) is shown above. Next, we interpret each line in detail:

**Line 1** net: "G:/train_model_new/Net2/ train_test_network.prototxt" (the address of network file)

**Line 2** test_iter: 2255

This is to be understood in conjunction with the batch_size in test layer. The total number of samples in the validation set in the Panicle-SEG data is 225,472. The one-time execution of all data is inefficient, so we divide the test data into batches to execute, and the number of samples in each batch is batch_size. In this paper, the batch_size was set as 100 for the validation set, so, we need to iterate 2,255 times to complete all 225,472 of the data. So test_iter is set to 2,255.

**Line 3** test_interval: 9020

In this paper, the batch_size was set as 100 for the training process, so, we need to iterate 9,020 times to complete all 901,895 samples in the training set. So, test interval was 9020, which meant training 9020 times before a test. Once all the training data has been performed, a test is done.

**Line 4-13** Settings for the learning rate. As long as the gradient descent method is used to solve optimization, there will be a learning rate, also known as step. "base_lr" is used to set the base learning rate, and in the iterative process, the learning rate can be adjusted. How to adjust, is decided by "lr_policy" to set (we use "multistep" method). In line 9-13, "stepvalue" represents changing the learning rate after "stepvalue" iterations per training session and the value of the "gamma" was used for updating learning rate (The new learning rate is base_lr by gamma). The "momentum" in line 5 means the optimization method adopts the SGD+Momentum and "weight_decay" in line 6 is a weight attenuation parameter (a parameter to prevent the fitting).

**Line 14** 100 times per training, displayed on screen once.

**Line 15** max_iter: 600000. The value reflects the maximum number of iterations. Small max_iter will cause convergence and low accuracy, while too large max_iter can cause concussion and time waste.

**Line 16-17** "snapshot". Save the training model and current state. "snapshot" was used to set how many times after the training to save. "snapshot_prefix" is the save path.

**Line 18** solver_mode: GPU

Set the run mode. The default is GPU, if you do not have GPU, then you need to change to CPU mode.

**Step5:** Scripting train.sh, then use Git to run (The codes are listed as below). Start training.

(train.sh)

Here, fine-tuning is applied by using format like "--model". Fine-tuning takes an already learned model, adapts the architecture, and resumes training from the already learned model weights. The Panicle-SEG patches are visually similar to the Cifar10 dataset, on which the model (cifar10_quick_iter_400000.caffemodel) was trained. Since that model works well for object category classification, we’d like to use this architecture for our style classifier. If we provide the weights argument to the Caffe train command, the pre-trained weights will be loaded into our model, matching layers by name. Because we are predicting 2 classes instead of a 10, we do need to change the last layer in the model. Additionally, we set stepsize in the solver to a lower value than if we were training from scratch. The start of the base learning rate is 0.001. Then, we decreased the learning rate by a factor of 0.3 for 20,000 iterations, 100,000 iterations, 160,000 iterations, 240,000 iterations and 320,000 iterations. At the same time, we set the start value of the momentum to 0.9, which remained unchanged. And the maximum number of iterations is set to 600,000. The final training model (Panicle-SEG-CNN) is saved to disk according to the Google Protocol Buffers, which can be called by using the Caffe C++ interface in the testing process.

Training log:


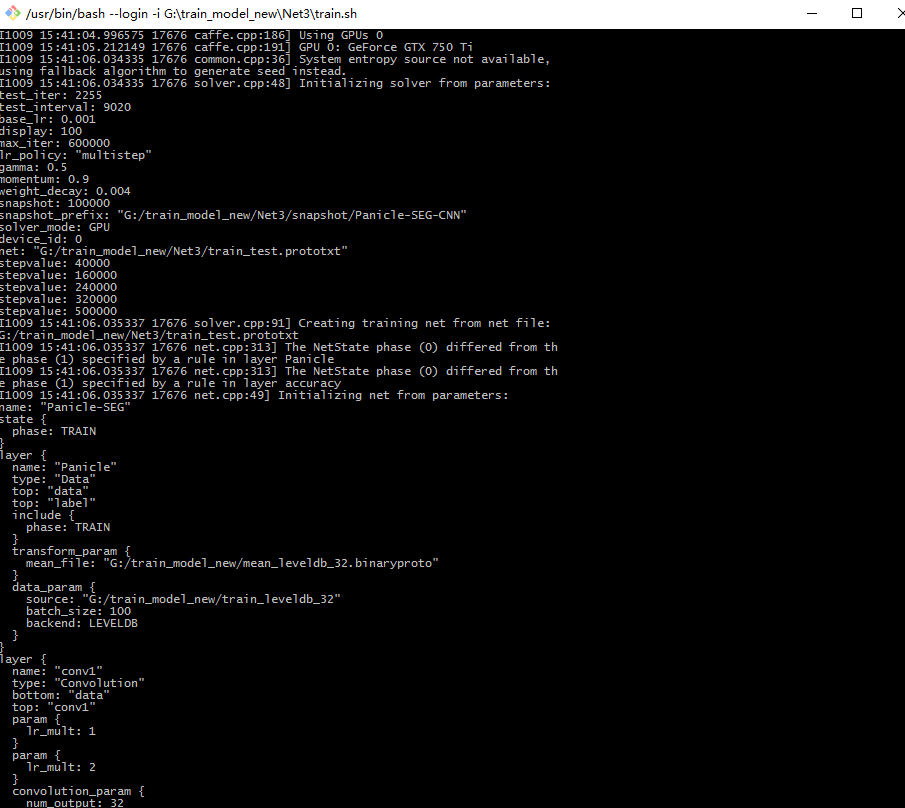


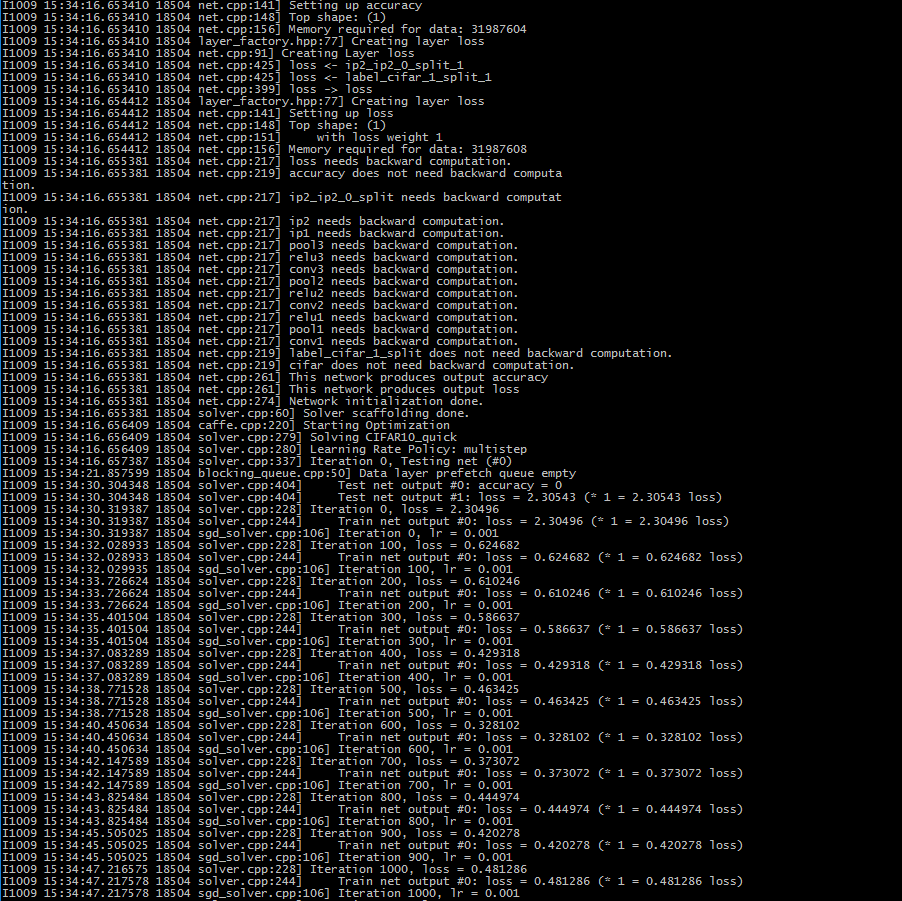


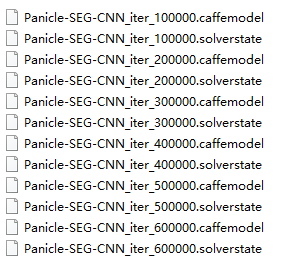


Then, we got the final Panicle-SEG-CNN training model (Panicle-SEG-CNN_iter_600000.caffemodel).

**Part2: On-line segmentation**

**(1) Coarse segmentation by using the pre-trained Panicle-SEG-CNN model**

To test field rice images with high resolution, the first operation is testing patches generation. The SLIC superpixel algorithm also applied here to generate testing patches. Then, we ran each test patch through the pre-trained model, obtaining the likelihood of each class. The SLIC superpixel region, which is corresponding in the positive testing patch, will be retained in the coarse segmentation result for candidate rice panicle area. In turn, the SLIC superpixel regions that are classified as negative testing patch will be removed in the coarse segmentation result for confirmed background. Just as previous discussed, the coarse segmentation result is likely to contain some background pixels. Meantime, the CNN classification can't ensure that all the testing patches are classified correctly, which will impact the effectiveness of coarse segmentation result (Figure 13A). So, the following optimization algorithm is needed.

The corresponding code for generating coarse segmentation result in C + + language as:

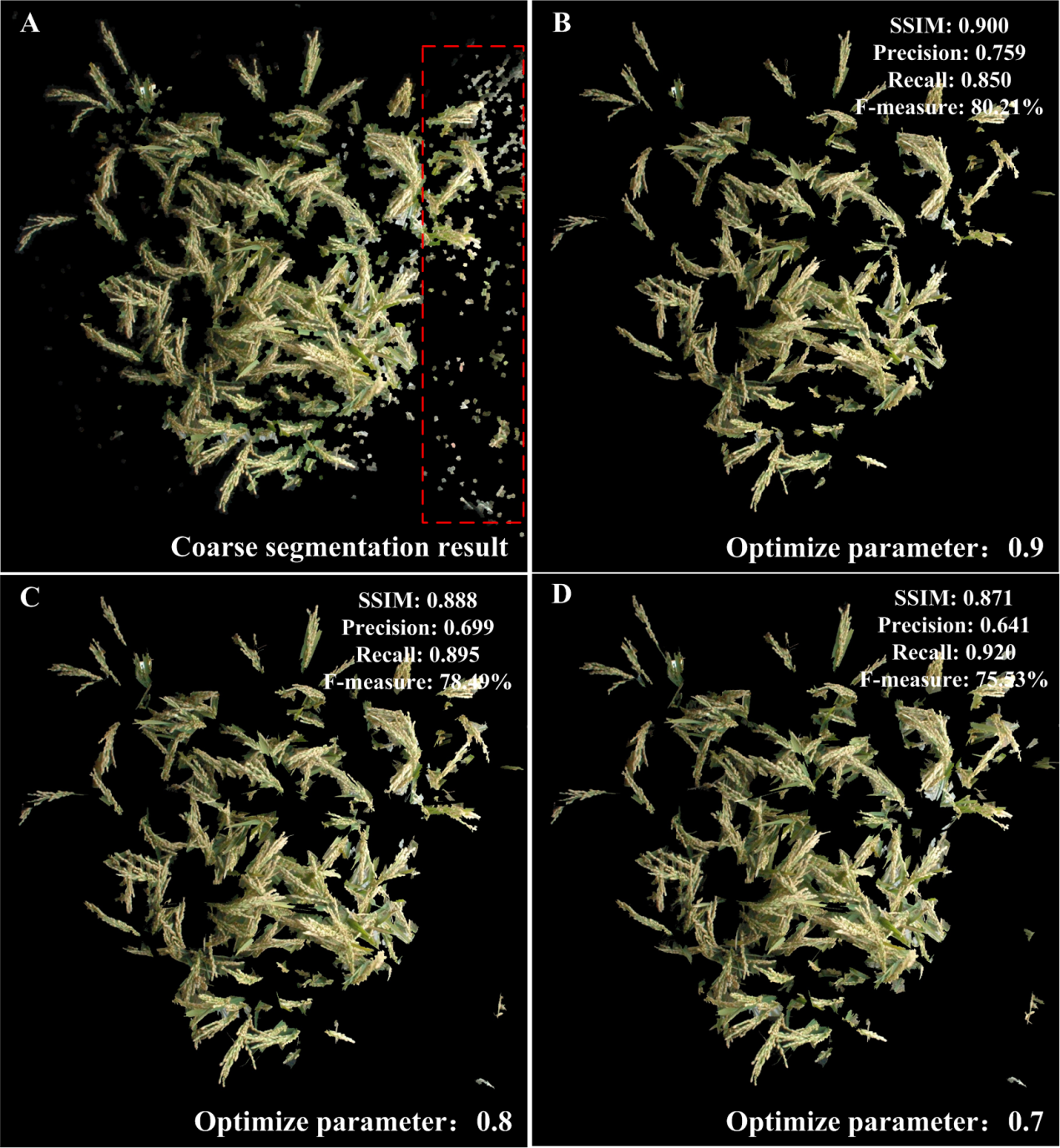


**Figure 13 Discussion for different optimize parameters.** Figure (A) shows the coarse segmentation result for a test top-view rice image. (B), (C), (D) represents the difference of optimize parameter form 0.9 to 0.7. In figure (A), the coarse segmentation result is likely to contain some background regions. Meantime, the CNN classification can't ensure all the testing patches being classified correctly, especially in the red dotted box. Obviously, the optimize parameter has great impact on final segmentation result. As the optimize parameter is decreasing, so is their completeness for rice panicle. For instance, the value of Recall is promoted from 0.85 to 0.92 with the optimize parameter from 0.9 to 0.7. In contrast, the precision will decrease significantly from 0.76 to 0.64, which means that larger impurity (background pixels) is brought to final segmentation result. Synthesizes these two indicators and SSIM, the optimize parameter at 0.9 or even larger, like 0.95, is good options for segmentation optimization.

**(2) Entropy rate superpixel** **optimization**

In Figure 13A, the coarse segmentation result is likely to contain some background regions. Meantime, the CNN classification can't ensure all the testing patches being classified correctly, especially in the red dotted box. So, optimization is necessary. Entropy rate superpixel segmentation [7] is another superpixel algorithm is used in this paper. The entropy rate superpixel algorithm with less number of superpixel regions can achieve approving effect for boundary adherence. Meantime, entropy rate superpixel image has larger background regions and relatively small foreground panicle regions, which provides new ideas for segmentation optimization.

The process for entropy rate superpixel optimization is as follows: Firstly, we assume that the coarse segmentation is correct. In this place, all the testing patches classified as candidate rice panicle area at coarse segmentation result were marked as grey value of 255 (panicle pixels) and the remaining testing patches marked as grey value of 0 (background pixels). Then, the number of panicle pixels and background pixels within each entropy rate superpixel region are calculated. After that, the category of entropy rate superpixel region will be determined according to the panicle pixel ratio, which was defined as the ratio between the number of panicle pixels and total pixel area for each entropy rate superpixel region. Here, a satisfactory result can be got by using the optimization parameter 0.9 in this paper. The entropy rate superpixel region will be considered as final rice panicle region if the panicle pixel ratio of the superpixel is above the optimization parameter 0.9. The final segmentation result will be joining together by combining the panicle entropy rate superpixel regions and removing small area regions (lower than 500 pixels).

The proposed entropy rate superpixel segmentation, as a high-efficiency and strong-feasibility optimization algorithm, owns its unique advantages in the following several aspects. In the first place, there are errors at the step of CNN classification in coarse segmentation result. Some background SLIC superpixel regions may be classified as candidate panicle regions. Commonly, relative to the large entropy rate superpixel background region, the area of error SLIC superpixel region accounts for a relatively small proportion, leading to a panicle pixel ratio smaller than the optimization parameter in entropy rate optimization algorithm. So, after the optimization, the segmentation noise caused by error classification can be significantly suppressed. Secondly, as the edge of SLIC superpixel regions is relatively well-regulated, the foreground pixels and background pixels may exist in the same SLIC superpixel region. For example, say these regions are classified as candidate panicle regions, leading to poor edge segmentation result in coarse result. In contrast, entropy rate superpixel segmentation has a stronger ability for edge preservation, which will keep the accuracy and the completeness of edge extraction after optimization algorithm. Furthermore, because of the errors in CNN classification, some candidate panicle regions may be classified as background, which will cause the gap of rice panicle region. This can be solved, to a certain extent, by adopting entropy rate generation algorithm. If the pixel area of the incorrectly classified rice panicle is relative small compared to the remaining part of rice panicle which is recognized correctly in the entropy rate superpixel region, then the gap has the potential to be filled up.

In optimization, there are three parameters that need to be predefined:

(1) The number of entropy rate superpixel regions

Also, Figure 14 (A), (B) and (C) represents the number of entropy rate superpixel region is 1000, 5000 and 10000, respectively. In (A), the algorithm has bad capability in boundary adherence, particularly in white dotted box, where the background pixels and panicle pixels may mix in the same superpixel region. But, when the region number gets enlarged (C), the area of background superpixel region is relatively small compared with figure (B). So, once the error for CNN classification happens, the area of error SLIC superpixel region accounts for a relatively large proportion, which can hardly achieve the requirements for final optimization. So, a relatively small entropy rate superpixel region, around 5000 (for image with resolution of 1815×1971 pixels), is a good option. For the testing samples with resolution of 1815×1971 pixels, the 5000 will stay the same for all the testing process. Like the number of SLIC superpixel region, for different size of input images in the testing process, the fixed proportional relation (1815×1971/5000) was used to calculate the number of entropy rate superpixel region for a new testing sample. The calculation formula is expressed in C + + language as:

where, image.rows and image.cols was the number of rows and columns in the testing sample.


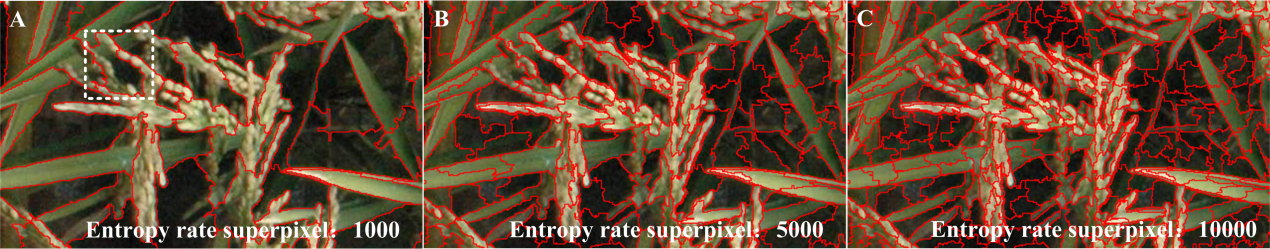
**Figure 14 The discussion for region number of entropy rate superpixel algorithm.** (A), (B), (C) represents the number of entropy rate superpixel region is 1000, 5000 and 10000, respectively. In (A), the algorithm has bad capability in edge-preserving, for instance in white dotted box. The background pixels and panicle pixels may mix in the same superpixel region. As the number of entropy rate superpixel region is increasing, so is their ability for edge-preserving. But, when the region number is too large, like figure (C), the area of background superpixel region is relatively small compared with figure (B). In this way, it can hardly achieve the optimization. So, the parameter 5000 for entropy rate superpixel optimization is a good option.

(2) Balancing parameter (lambda): 0.5 (revise the parameters if needed and the range of it is from 0 to 1)

In practical applications, the guidelines of parameter settings for Panicle-SEG algorithm have a great impact on the final segmentation effect. The balancing parameter (lambda) ranged from 0 to 1 is an important indicator in entropy rate superpixel (ERS) segmentation. Figure 15A-E shows the ERS segmentation images with different lambda value range from 0.1 to 0.9. Smaller lambda results in poor boundary adherence for rice panicle, while the areas of background region are large enough for optimization algorithm. With the increase of lambda, the entropy rate superpixel regions will be tinier, which will not good at segmentation optimization. So, the value of lambda cannot be too big or too small. And From the point of SSIM [8] and F-measure [9], the value from 0.5 to 0.9 has little effect on final segmentation performance. So, the lambda from 0.5 to 0.9 yields an optimum selecting among these metrics. In this study, 0.5 is used as default value for the testing set with resolution of 1815×1971 pixels. For other size of testing sample, changing the value of lambda may have better segmentation result.


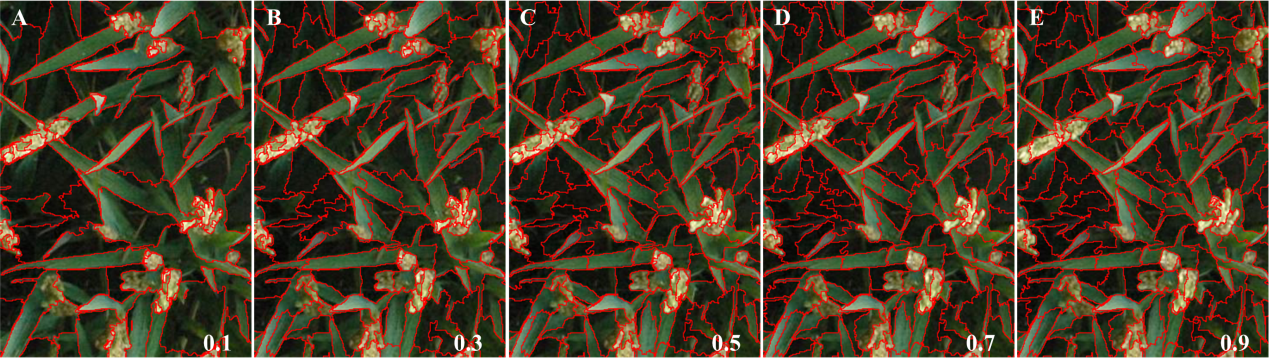


**Figure 15** **Influence of** **balancing parameter in entropy rate superpixel algorithm.** Clearly, the competitive segmentation results are achieved with a wide range of balancing parameter selection (A), (B), (C), (D) and (E). Smaller lambda results in poor edge-preserving for rice panicle, while the areas of background regions are large enough for optimization algorithm. But, with the increase of lambda, the entropy rate superpixel regions will be tinier, which will not be good for segmentation optimization. So, empirically, we found that lambda from 0.5 to 0.9 yields a good compromise among these metrics.

(3) Optimization parameter (0.9 in this paper)

Optimization parameter was defined as the ratio between the number of panicle pixels and total pixel area for each entropy rate superpixel region, and the Figure 13 shows the discussion for different optimization parameter form 0.7 to 0.9. In Figure 13A, the coarse segmentation result is likely to contain some background regions. Meantime, the CNN classification can't ensure all the testing patches being classified correctly, especially in the red dotted box. Obviously, the optimization parameter has great impact on final segmentation result. As the optimization parameter is decreasing, so is their completeness for rice panicle. For instance, the value of Recall is promoted from 0.85 to 0.92 with the optimize parameter from 0.9 to 0.7. In contrast, the precision will decrease significantly from 0.76 to 0.64, which means that larger impurity (background pixels) is brought to final segmentation result. Synthesizes these two indicators and SSIM, the optimization parameter at 0.9 or even larger, like 0.95, is good options for segmentation optimization. In this paper, 0.9 is applied. For other size of testing sample, changing the value of lambda may have better segmentation result. Then, how to change it is depends on whether your wants to segment more accurately or segment more completely. The corresponding code is expressed in C + + language as:

For the convenience of user, we have reserved the interface of parameter adjusting in the software, the experience coefficient and the lambda can be adjusted outside the software.

**Speed up the segmentation project**

In this study, the whole segmentation project is developed in C++ using the OpenCV library [10]. Also, the Caffe framework for CNN classification was encapsulated in the project by calling the C++ interface.

The OpenMP [11], as an application programming interface to supports multi-platform shared memory in C++, was applied to speed up the project. The performance of acceleration depends on CPU frequency and the number of CPU cores. OpenMP is an abbreviation for Open multiprocessing, which is not a simple library of functions, but a framework supported by many compilers, or a protocol, in short, without any configuration, you can use it in Visual Studio. Meantime, Caffe's integration with CUDA and cuDNN library accelerates Caffe processing models. CUDA is a parallel computing platform created by NVIDIA and the cuDNN library is developed for deep learning in GPU acceleration. The uses of high-performance GPU have changed the traditional opinions about acceleration and greatly shorten the processing time of segmentation algorithm. Here, the parallel computing using GPU is adopted in CNN classification. In this way, the time for rice panicle segmentation can achieve about 60 to 70 seconds per testing top-view field rice image with the resolution of 1815×1971 pixels.

In order to evaluate the segmentation time, we tested the different resolution field rice images (top-view and overhead-view) and indoor pot-grown rice images (top-view and side-view), respectively.

(1) In GPU mode, the test log as follow:

The overhead_view_field (3904×3693 pixels) about 8 minutes in GPU mode

The top_view_field (1815×1971 pixels) about 70 seconds in GPU mode

The side_view_indoor (750×1580 pixels) about 18 seconds in GPU mode

The top_view_indoor (462×398 pixels) about 2.2 seconds in GPU mode

(2) In CPU mode, the test log as follow:

The overhead_view_field (3904×3693 pixels) about 10.3 minutes in GPU mode

The top_view_field (1815×1971 pixels) about 90.4 seconds in GPU mode

The side_view_indoor (750×1580 pixels) about 23.6 seconds in GPU mode

The top_view_indoor (462×398 pixels) about 3.3 seconds in GPU mode

**Evaluation criteria, testing log and segmentation results**

**(1) Evaluation criteria**

To evaluate the performance of the segmentation, four indicators, including the Structural Similarity Index (SSIM) [8], Precision, Recall, and the F-measure [9] are adopted. Among them, the SSIM is applied to describe the degree of similarity between the segmentation images and the ground truth images. The SSIM model analyzes the structure of the image information from three aspects, including the brightness, structure similarity and contrast. The range of the SSIM is from 0 to 1, and the higher the value is, the more similar the two images are. Precision and Recall are the most basic indicators to reveal the final segmentation results. Precision illustrates the accuracy of the segmentation algorithm, and Recall represents the completeness of the segmented rice panicles. The computational formulas for Precision and Recall are provided in Equations 5-6. In practice, Precision and Recall interact with each other. When Precision is high, Recall will be low. Sometimes, we need to balance these two indicators. To accomplish this goal, the F-measure is proposed. The computational formula is shown in Equation 7. The higher the value of the F-measure is, the more perfect the rice panicle segmentation will be.

$Precision=\frac{TP}{TP+FP}$ (5)

$Recall=\frac{TP}{TP+FN}$ (6)

$F=\frac{2\times Precision\times Recall}{Precision+Recall}\times\text{100 (\%)}$ (7)

Where, TP, TN, FP, and FN represent the numbers of true positives, true negatives, false positives, and false negatives, respectively. True positives (TP) are when the predicted results and the corresponding ground truth are both rice panicle pixels. True negatives (TN) represent that the predicted results and the corresponding ground truth are both background pixels. False positives (FP) were determined as those pixels that were classified as rice panicle pixels, but the ground truth of those pixels are background. The False negatives (FN) are those pixels that belong to the ground truth, but they are not correctly discriminated. The corresponding code is expressed in C + + language as:

Not only that, the accuracy of the segmentation methods is also evaluated by the following performance measurements (Equation 8-9) [12-13]:

$Q\text{seg}=\frac{\sum_{i=0}^{n} \sum_{j=0}^{m} (\mathrm{A}(v)_{i,j}\cap\mathrm{B}(v)_{i,j})}{\sum_{i=0}^{n} \sum_{j=0}^{m} (\mathrm{A}(v)_{i,j}\cup\mathrm{B}(v)_{i,j})}$ (8)

$Sr=\frac{\sum_{i=0}^{n} \sum_{j=0}^{m} (\mathrm{A}(v)_{i,j}\cap\mathrm{B}(v)_{i,j})}{\sum_{i=0}^{n} \sum_{j=0}^{m} \mathrm{B}(v)_{i,j}}$ (9)

Where A is the set of the panicle pixels (v=255) or background pixels (v=0) identified by our Panicle-SEG, and B is a reference set of manually segmented panicle pixels (v=255) or background pixels (v=0). The value of m and n reflects the image row and column sizes and i, j are the pixel coordinate indices of the images. The range of Qseg and Sr is from 0 to 1. Namely, the higher the value (approach to 1), the more accurate the segmentation is. Conversely, the closer the value is to 0, the worse the consistency is. So, the different is that the value of Qseg represents the consistency of both panicle part and background part while the value of Sr represents the consistency of only panicle part. The corresponding code is expressed in C + + language as:

**(2) Testing log and segmentation results**

In order to evaluate the segmentation result of the Panicle-SEG algorithm, we expanded the testing set and made a total of 48 images, including 24 top-view field rice images, 12 overhead-view field rice images, 7 pot-grown side-view images and 5 pot-grown top-view images. The testing samples were made available online at: *http://plantphenomics.hzau.edu.cn/checkiflogin_en.action* (username: UserPP; password: 20170108pp). And, the value of the evaluation criterion for whole testing samples using Panicle-SEG algorithm is shown in the Table 1.

**Table 1. The evaluation criterion for 48 testing rice samples**

| **Number** | **Qseg** | **Sr** | **SSIM** | **Precision** | **Recall** | **F-measure** |
| --- | --- | --- | --- | --- | --- | --- |
| **1** | 0.563 | 0.655 | 0.853 | 0.800 | 0.655 | 0.721 |
| **2** | 0.589 | 0.706 | 0.821 | 0.780 | 0.706 | 0.741 |
| **3** | 0.666 | 0.742 | 0.828 | 0.866 | 0.742 | 0.800 |
| **4** | 0.489 | 0.661 | 0.904 | 0.652 | 0.661 | 0.657 |
| **5** | 0.540 | 0.641 | 0.855 | 0.775 | 0.641 | 0.702 |
| **6** | 0.587 | 0.726 | 0.859 | 0.754 | 0.726 | 0.740 |
| **7** | 0.611 | 0.707 | 0.847 | 0.819 | 0.707 | 0.759 |
| **8** | 0.589 | 0.721 | 0.838 | 0.763 | 0.721 | 0.741 |
| **9** | 0.601 | 0.694 | 0.860 | 0.817 | 0.694 | 0.751 |
| **10** | 0.696 | 0.751 | 0.783 | 0.905 | 0.751 | 0.821 |
| **11** | 0.485 | 0.559 | 0.879 | 0.785 | 0.559 | 0.653 |
| **12** | 0.618 | 0.687 | 0.844 | 0.860 | 0.687 | 0.764 |
| **13** | 0.595 | 0.706 | 0.861 | 0.791 | 0.706 | 0.746 |
| **14** | 0.631 | 0.743 | 0.882 | 0.808 | 0.743 | 0.774 |
| **15** | 0.580 | 0.640 | 0.756 | 0.862 | 0.640 | 0.734 |
| **16** | 0.489 | 0.535 | 0.819 | 0.850 | 0.535 | 0.657 |
| **17** | 0.551 | 0.587 | 0.820 | 0.901 | 0.587 | 0.711 |
| **18** | 0.604 | 0.645 | 0.807 | 0.905 | 0.645 | 0.753 |
| **19** | 0.676 | 0.746 | 0.887 | 0.877 | 0.746 | 0.806 |
| **20** | 0.693 | 0.748 | 0.849 | 0.903 | 0.748 | 0.819 |
| **21** | 0.555 | 0.612 | 0.936 | 0.855 | 0.612 | 0.713 |
| **22** | 0.675 | 0.756 | 0.855 | 0.863 | 0.756 | 0.806 |
| **23** | 0.526 | 0.681 | 0.925 | 0.699 | 0.681 | 0.690 |
| **24** | 0.482 | 0.548 | 0.899 | 0.800 | 0.548 | 0.651 |
| **25** | 0.644 | 0.874 | 0.858 | 0.735 | 0.837 | 0.783 |
| **26** | 0.635 | 0.814 | 0.864 | 0.742 | 0.814 | 0.777 |
| **27** | 0.640 | 0.731 | 0.894 | 0.836 | 0.731 | 0.780 |
| **28** | 0.712 | 0.873 | 0.779 | 0.795 | 0.873 | 0.832 |
| **29** | 0.648 | 0.763 | 0.520 | 0.811 | 0.763 | 0.786 |
| **30** | 0.657 | 0.876 | 0.943 | 0.724 | 0.876 | 0.793 |
| **31** | 0.626 | 0.775 | 0.896 | 0.765 | 0.775 | 0.770 |
| **32** | 0.643 | 0.720 | 0.889 | 0.857 | 0.720 | 0.782 |
| **33** | 0.601 | 0.696 | 0.976 | 0.814 | 0.696 | 0.750 |
| **34** | 0.662 | 0.758 | 0.970 | 0.840 | 0.758 | 0.797 |
| **35** | 0.690 | 0.809 | 0.934 | 0.825 | 0.809 | 0.817 |
| **36** | 0.675 | 0.803 | 0.966 | 0.809 | 0.803 | 0.806 |
| **37** | 0.583 | 0.641 | 0.990 | 0.864 | 0.641 | 0.736 |
| **38** | 0.713 | 0.796 | 0.989 | 0.872 | 0.796 | 0.832 |
| **39** | 0.729 | 0.821 | 0.986 | 0.866 | 0.821 | 0.843 |
| **40** | 0.633 | 0.673 | 0.986 | 0.914 | 0.673 | 0.775 |
| **41** | 0.635 | 0.647 | 0.986 | 0.971 | 0.647 | 0.777 |
| **42** | 0.723 | 0.750 | 0.979 | 0.952 | 0.750 | 0.839 |
| **43** | 0.840 | 0.878 | 0.986 | 0.950 | 0.878 | 0.913 |
| **44** | 0.619 | 0.797 | 0.963 | 0.735 | 0.797 | 0.765 |
| **45** | 0.603 | 0.894 | 0.992 | 0.650 | 0.894 | 0.752 |
| **46** | 0.681 | 0.763 | 0.994 | 0.863 | 0.763 | 0.810 |
| **47** | 0.614 | 0.852 | 0.988 | 0.687 | 0.852 | 0.761 |
| **48** | 0.730 | 0.855 | 0.993 | 0.834 | 0.855 | 0.844 |
| **Mean** | **0.626** | **0.730** | **0.891** | **0.821** | **0.730** | **0.767** |
| **Std^a^** | **0.072** | **0.090** | **0.088** | **0.074** | **0.089** | **0.055** |

a. The standard deviation value

Moreover, the Panicle-SEG can be expanded for different field environments, different camera angles, different reproductive stages, and even indoor rice images. The following are described separately in detail.

1. Different field environments (This is mainly measured by the differences in the morphological structure of the panicle, differences in illumination and differences in weather conditions).

Figure16 shows the differences in the morphological structure of the panicle. In Figure 16A, the upright panicles are partially hidden in the rice leaf blade. While, in Figure 16B, the bend growth panicles are basically exposed above the rice leaf blade and the panicles have many forked structures. And, the awn exists in the rice panicle in Figure 16C. So, the size, shape and pose are different. Using our algorithm, all of these situations can be well solved, and the consistency and precision of the segmentation result is also good.


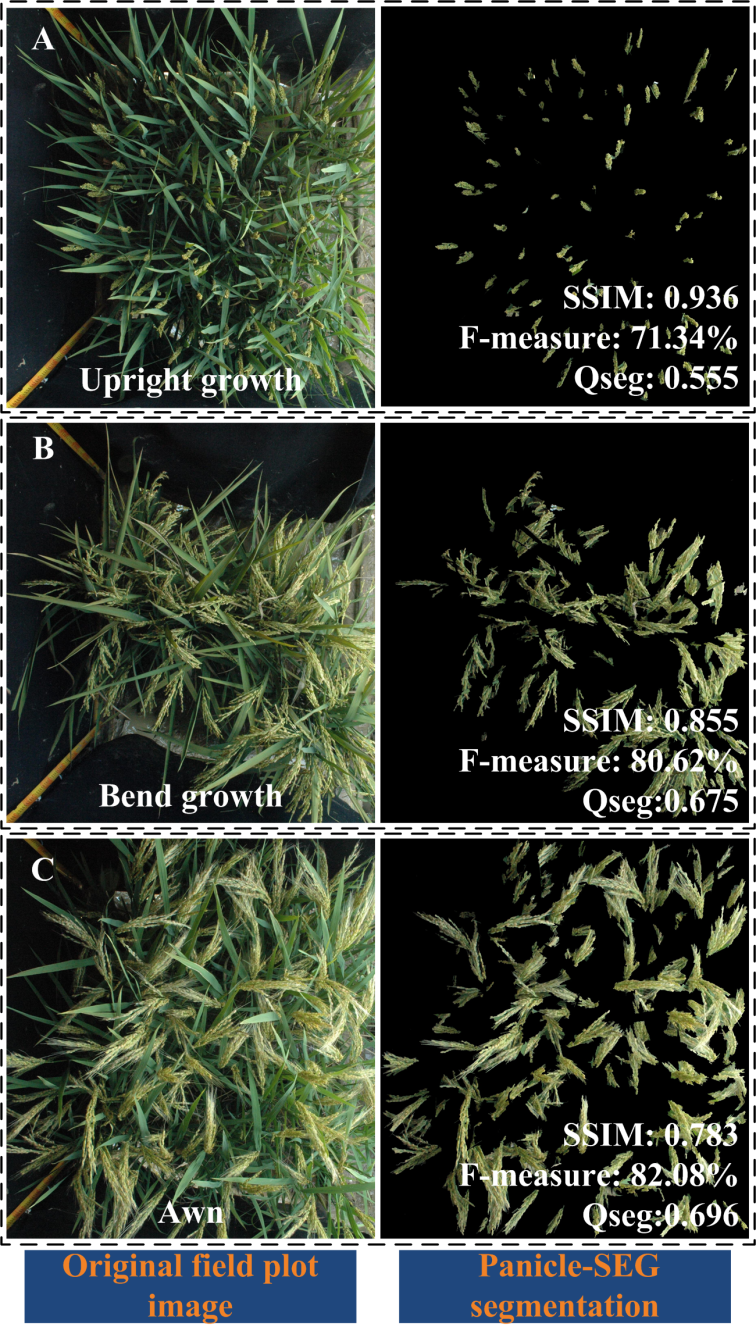


**Figure 16 The differences in the morphological structure of the panicle using Panicle-SEG algorithm.**

Figure 17 and Figure 18 depict the robustness of our Panicle-SEG algorithm for the different illumination condition. Figure 17 shows the differences in illumination from the view of whole image. The illumination in Figure 17A is significantly brighter than Figure 17B. The value of SSIM for the two segmented images can reach more than 0.89, which reflects that the segmented images and the ground truth images have a high degree of similarity. And, the average of Qseg is about 0.62. So, the proposed algorithm has better consistency of both panicle foreground part and background part even in different illumination condition. Furthermore, the difference in illumination is not only in different field plot images, but also in different areas of the same image (Figure 18). Figure 18A is the original overhead-view field rice image in testing samples. Figure 18B and Figure 18C show the different illumination condition in different areas of the image. It can be seen that the brightness of Figure 18B is darker, and the panicle area is not obvious. In this way, the proposed Panicle-SEG algorithm can still solve this problem well.

**
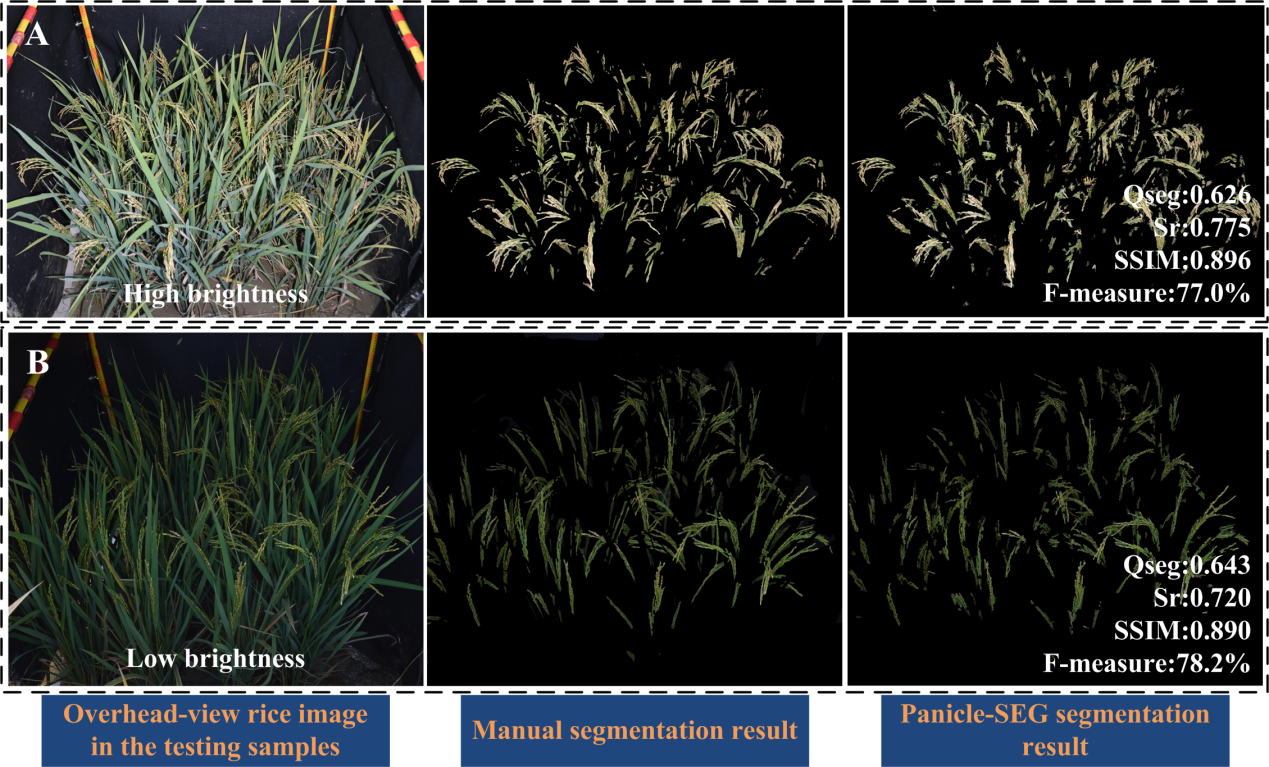
**

**Figure 17 The differences in illumination by using Panicle-SEG algorithm.**

**
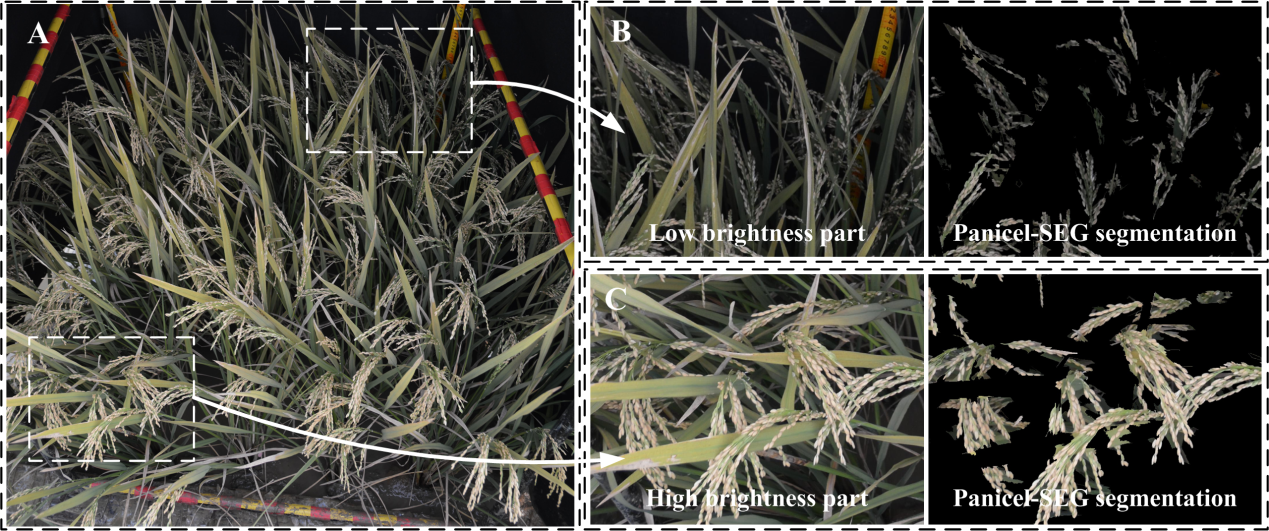
**

**Figure 18 Different regions in the field plot image have different illumination condition.**

Weather condition is another problem to influence the color and illumination of the panicle. Figure 19 shows the differences in weather conditions by using Panicle-SEG algorithm. In sunny day (Figure 19A), the image's illumination is bright. But, in cloudy weather (Figure 19B), affected by the weather, the light is not very sufficient, so the entire image's illumination is dim, while the color of panicle is gray. In this way, the proposed Panicle-SEG algorithm can still solve this problem well.

**
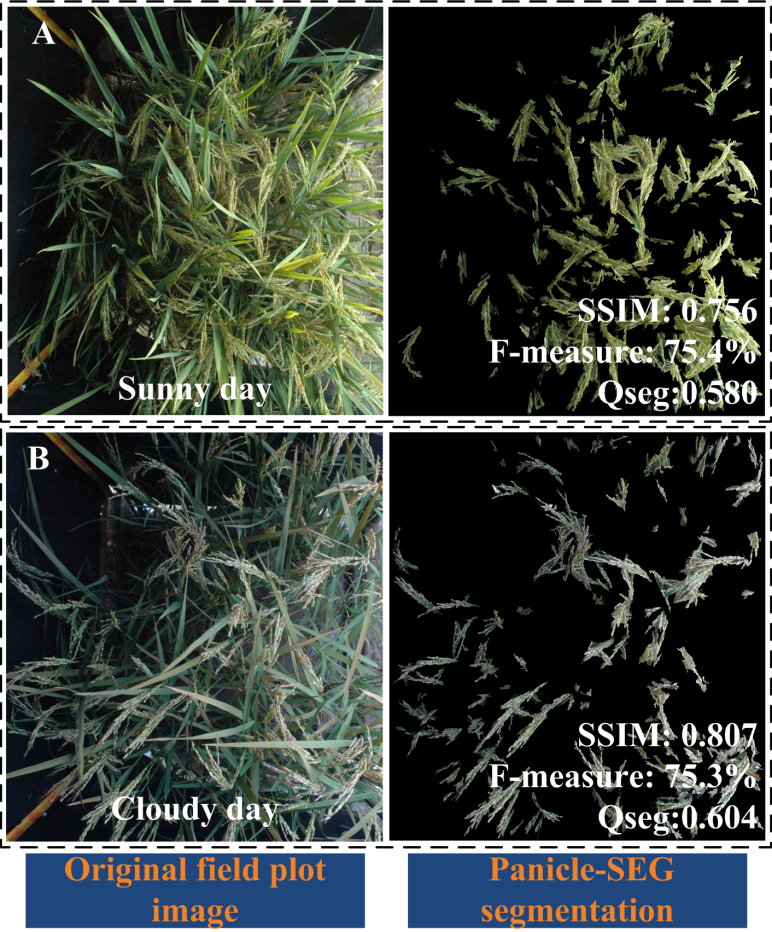
**

**Figure 19 The differences in weather conditions by using Panicle-SEG algorithm.**

2. Different camera angles and different reproductive stages

In the field environment, the overhead-view is also commonly used for field rice image acquisition. Figure 20A-C show three overhead-view field rice images with different reproductive stages (heading stage, filling stage and mature stage). The second column in Figure 20A-C represents the manual segmentation results using PhotoShop software. And the last column reflects the corresponding Panicle-SEG segmentation result. For heading stage in Figure 20A, the Qseg, Sr, SSIM, Precision, Recall, and the F-measure (%) can achieve 0.662, 0.758, 0.970, 0.840, 0.758, and 79.7%, respectively. For filling stage in Figure 20B, the Qseg, Sr, SSIM, Precision, Recall, and the F-measure (%) are 0.657, 0.876, 0.943, 0.724, 0.876 and 79.3%, respectively. And for mature stage in Figure 20C, the Qseg, Sr, SSIM, Precision, Recall, and the F-measure (%) are 0.712, 0.873, 0.779, 0.795, 0.873 and 83.2%, respectively. In this way, the proposed Panicle-SEG segmentation algorithm can not only adapt to different camera angles, but also has good segmentation result for different growth periods.


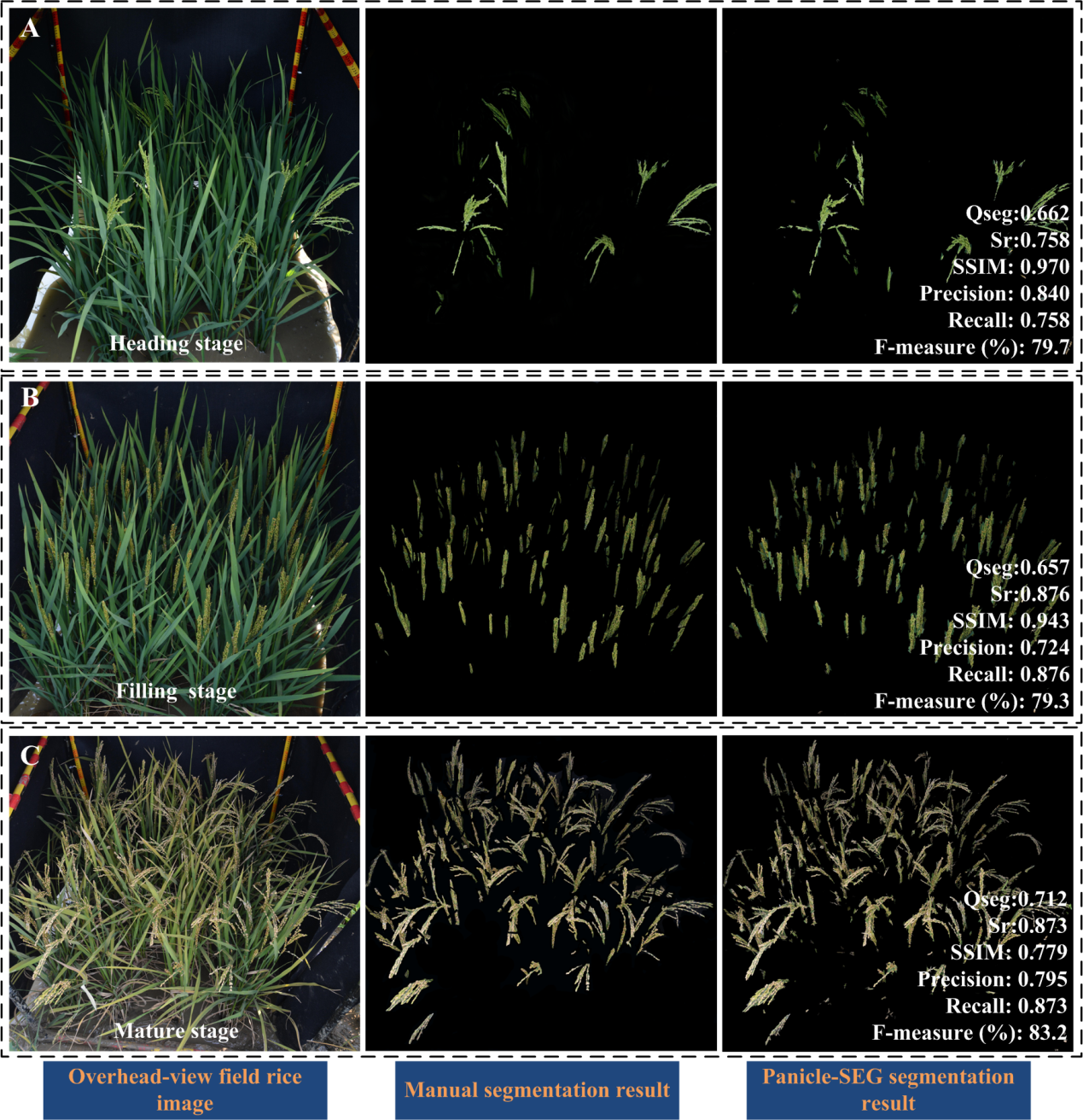


**Figure 20 Panicle segmentation results under an overhead view in the field environment.**

(3) Indoor rice images can also be segmented.

As a highly-robust panicle segmentation algorithm, the indoor potted rice images acquired by the RGB camera from top-view and side-view are also suitable for panicle segmentation using Panicle-SEG. The original indoor rice images, manually segmented images, and Panicle-SEG segmented results are shown in the left column, center column, and right column of Figure 21, respectively. As illustrated in Figure 21, the F-measure (%) is above 84%, the Qseg is above 0.7 and the SSIM values are all above 0.99. Thus, the proposed Panicle-SEG algorithm also has strong flexibility for indoor top-view rice images (Figure 21A), indoor side-view rice images with green leaves (Figure 21B), and indoor side-view rice images with yellow leaves (Figure 21C).


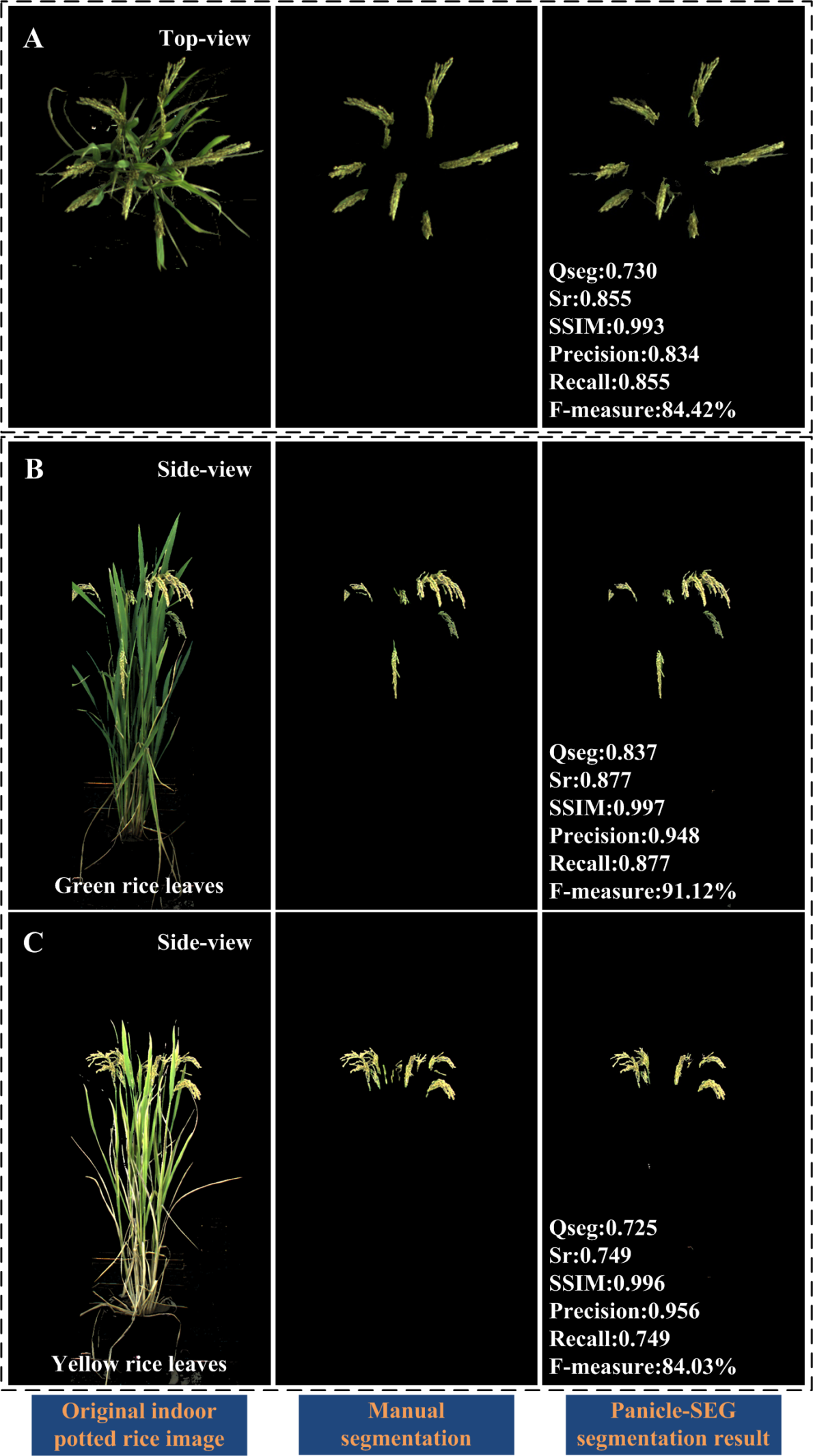


**Figure 21 Panicle segmentation results for indoor pot-grown rice images.**

**Software** **implementation procedure**

The proposed project name is "Panicle-SEG", which is designed for rice panicle segmentation. Since there is no interface, the whole project needs to run in command line mode. And the software has been tested on 64-bit Windows 7 system and 64-bit Windows 10 system in the CPU and GPU mode. If you want to download the testing samples and installable file, you can click the web link below:

*http://plantphenomics.hzau.edu.cn/checkiflogin_en.action* (username: UserPP; password: 20170108pp)

How to use the software can refer to the **Additional File 1: Video S1**. And the detailed software implementation procedure as follows: (1) Download the new version of Panicle-SEG installable file, you can choose CPU or GPU mode. (2) Install the "setup_Panicle-SEG_cpu.exe" if you choose the CPU mode or "setup_Panicle-SEG_gpu.exe" file if you choose the GPU mode. (3) Open the command line in your computer and enter to the current file path. (4) Open the "Readme.txt" and revise the parameters if needed. The detailed explanations for the parameters were shown below. (5) Copy the revised content in "Readme.txt" and paste to the command line. (6) Waiting for rice panicle segmentation (the cost time depends on your computer performance). (6) The segmentation result is saved in the "segmentation_results" file. In step (4), an example format for Panicle-SEG software in command line:

***Panicle-SEG images_test/top_view_field.jpg 4 deploy1.prototxt cifar10_quick_iter_600000.caffemodel mean_leveldb_32.binaryproto label_filename.txt 0.5 0.9 500 segmentation_results/segmentation_top.jpg***

The detailed explanation for command line parameters as follows:

(1) Executable file name: ***Panicle-SEG*** (retain the default settings).

(2) Input rice image path and name: ***images_test/***.jpg*** (Defaults to current path if not specified).

(3) The number of CPU cores: ***4*** (revise the parameters if needed).

(4) The default CNN configuration files: (retain the default settings):

***deploy1.prototxt, cifar10_quick_iter_400000.caffemodel, mean_leveldb_32.binaryproto, label_filename.txt***

(5) Balancing parameter (also called lambda): ***0.5*** (revise the parameters if needed and the range of it is from 0 to 1). The detailed description of the balancing parameter can refer to Additional File 7: Appendix S2.

(6) Optimization coefficient: ***0.9*** (revise the parameters if needed and the range of it is from 0 to 1). The detailed description of the balancing parameter can refer to Additional File 7: Appendix S2.

(7) The area threshold for removing small background regions: ***500*** pixel^2^ (revise the parameters if needed)

(8) The save path of the final segmentation result: ***segmentation_results/***.jpg*** (Defaults to current path if not specified)

**Reference**

[1] Long J, Shelhamer E, Darrell T. Fully convolutional networks for semantic segmentation. IEEE Conference on Computer Vision and Pattern Recognition. IEEE Computer Society, 2015:3431-3440.

[2] Girshick R, Donahue J, Darrell T, et al. Rich feature hierarchies for accurate object detection and semantic segmentation. Proceedings of the IEEE conference on computer vision and pattern recognition. 2014: 580-587.

[3] Achanta R, Shaji A, Smith K, et al. SLIC superpixels compared to state-of-the-art superpixel methods. IEEE Transactions on Pattern Analysis & Machine Intelligence, 2012, 34(11):2274.

[4] Rother C, Kolmogorov V, Blake A. "GrabCut": interactive foreground extraction using iterated graph cuts. ACM SIGGRAPH. ACM, 2004:309-314.

[5] Zivkovic Z. Improved Adaptive Gaussian Mixture Model for Background Subtraction. International Conference on Pattern Recognition. IEEE Computer Society, 2004:28-31.

[6] Jia Y, Shelhamer E, Donahue J, et al. Caffe:Convolutional Architecture for Fast Feature Embedding. 2014:675-678.

[7] Liu M Y, Tuzel O, Ramalingam S, et al. Entropy rate superpixel segmentation. Computer Vision and Pattern Recognition. IEEE, 2011:2097-2104.

[8] Wang Z, Bovik A C, Sheikh H R, et al. Image quality assessment: from error visibility to structural similarity. IEEE Transactions on Image Processing A Publication of the IEEE Signal Processing Society, 2004, 13(4):600.

[9] Powers D M W. Evaluation: From Precision, Recall and F-Factor to ROC, Informedness, Markedness & Correlation. Journal of Machine Learning Technologies, 2011, 2:2229-3981.

[10] Brahmbhatt S. Practical OpenCV. Apress, 2013.

[11] Chandra R, Dagum L, Kohr D, et al. Parallel programming in OpenMP. Morgan Kaufmann Publishers, 2001.

[12] Guo W, Rage U K, Ninomiya S. Illumination invariant segmentation of vegetation for time series wheat images based on decision tree model. Computers & Electronics in Agriculture, 2013, 96(6):58-66.

[13] Georgee M, Joãocamargo N. Verification of color vegetation indices for automated crop imaging applications. Computers & Electronics in Agriculture, 2008, 63(2):282-293.
